# *In Vitro* Culture Expansion Shifts the Immune Phenotype of Human Adipose-Derived Mesenchymal Stem Cells

**DOI:** 10.3389/fimmu.2021.621744

**Published:** 2021-03-10

**Authors:** Richard Jeske, Xuegang Yuan, Qin Fu, Bruce A. Bunnell, Timothy M. Logan, Yan Li

**Affiliations:** ^1^ Department of Chemical & Biomedical Engineering, FAMU-FSU College of Engineering, Florida State University, Tallahassee, FL, United States; ^2^ The National High Magnetic Field Laboratory, Florida State University, Tallahassee, FL, United States; ^3^ Department of Microbiology, Immunology, and Genetics, University of North Texas Health Science Center, Fort Worth, TX, United States; ^4^ Department of Chemistry and Biochemistry, Florida State University, Tallahassee, FL, United States; ^5^ Institute of Molecular Biophysics, Florida State University, Tallahassee, FL, United States

**Keywords:** adipose-derived mesenchymal stem cells, replicative senescence, NAD redox cycle, immune phenotype, transcriptomics, proteomics

## Abstract

Human mesenchymal stem or stromal cells (hMSCs) are known for their potential in regenerative medicine due to their differentiation abilities, secretion of trophic factors, and regulation of immune responses in damaged tissues. Due to the limited quantity of hMSCs typically isolated from bone marrow, other tissue sources, such as adipose tissue-derived mesenchymal stem cells (hASCs), are considered a promising alternative. However, differences have been observed for hASCs in the context of metabolic characteristics and response to *in vitro* culture stress compared to bone marrow derived hMSCs (BM-hMSCs). In particular, the relationship between metabolic homeostasis and stem cell functions, especially the immune phenotype and immunomodulation of hASCs, remains unknown. This study thoroughly assessed the changes in metabolism, redox cycles, and immune phenotype of hASCs during *in vitro* expansion. In contrast to BM-hMSCs, hASCs did not respond to culture stress significantly during expansion as limited cellular senescence was observed. Notably, hASCs exhibited the increased secretion of pro-inflammatory cytokines and the decreased secretion of anti-inflammatory cytokines after extended culture expansion. The NAD+/NADH redox cycle and other metabolic characteristics associated with aging were relatively stable, indicating that hASC functional decline may be regulated through an alternative mechanism rather than NAD+/Sirtuin aging pathways as observed in BM-hMSCs. Furthermore, transcriptome analysis by mRNA-sequencing revealed the upregulation of genes for pro-inflammatory cytokines/chemokines and the downregulation of genes for anti-inflammatory cytokines for hASCs at high passage. Proteomics analysis indicated key pathways (e.g., tRNA charging, EIF2 signaling, protein ubiquitination pathway) that may be associated with the immune phenotype shift of hASCs. Together, this study advances our understanding of the metabolism and senescence of hASCs and may offer vital insights for the biomanufacturing of hASCs for clinical use.

## Introduction

Human mesenchymal stromal or stem cells (hMSCs) are multipotent cells found in nearly all postnatal tissues and are located in or near perivascular niches (1, 2). Despite their presence in nearly all adult tissues, bone marrow-derived hMSCs (BM-hMSCs) are the most commonly studied and widely applied in clinical trials for various diseases (3, 4). Beyond multipotency, hMSCs are known for the induction of endogenous repair through immunomodulation *via* direct cell-cell contact and secretome (5–7). However, a significant barrier for hMSC biomanufacturing for clinical use is to provide sufficient cell numbers for use in patients either *via in vivo* isolation or *in vitro* expansion. Though as the most widely acknowledged source, human bone marrow contains low amount of hMSCs (~0.001–0.01%) (8), and it is impractical to directly apply hMSCs from bone marrow without *in vitro* culture expansion. Moreover, the extensive passaging of BM-hMSCs results in significant alterations on cellular behavior termed as replicative senescence, which compromises hMSCs’ therapeutic potentials (5, 9–12). For example, 1-year survival rates in graft *vs* host disease were found to drop from 75% (in patients who received BM-hMSCs from passages 1–2) to 21% (in patients who received passages 3–4 cells) (13). Due to the expansion limitation, hMSCs from other sources, such as adipose tissue-derived (hASCs) and dental pulp hMSCs, have attracted increasing scientific attention as alternatives for hMSC-based therapy. hASCs are highly abundant in adult human tissues and can be isolated using minimally invasive strategies (14, 15). Studies have demonstrated that adipose tissue could yield roughly 500-fold more hMSCs than bone marrow based on fibroblastoid-like colony selection (16). Based on minimal criteria established by the International Society for Cell & Gene Therapy (ISCT), hASCs in most studies exhibit similar morphology, multipotency and surface markers compared to BM-hMSCs (16, 17), and have been applied to preclinical studies as an alternative tissue source (18, 19).

Although hASCs share similar characteristics with bone marrow-derived hMSCs, some meaningful biological differences have been reported (17, 20, 21). For instance, hASCs exhibit higher adipogenic differentiation ability and immunomodulatory capacity indicated by higher secretion of some cytokines such as IL-6 and TGF-β1 (22). Moreover, hASCs exhibit significantly higher proliferative capacity and less senescent behaviors within the same time-frame under similar culture conditions compared to BM-hMSCs (17, 23). Thus, hASCs have the potential to be expanded through more passages to generate sufficient cell numbers. However, studies have found that the therapeutic efficacy of hASCs is inconsistent, especially for immune-regulatory properties. For instance, hASCs from diabetic donors exhibited impaired anti-inflammatory potential and upregulated production of pro-inflammatory factors (24). Meanwhile, some studies claimed the immediate loss of T cell regulation when hASCs were isolated and cultured *in vitro* (25). These studies indicate that isolation and *in vitro* culture of hASCs may impact their cellular behavior and immune phenotype although proliferation capacity of hASCs is sufficient. Therefore, it is critical to define quality assurance criteria to ensure therapeutic potential of expanded hASCs. Besides cellular phenotype and differentiation potential, immune phenotype and senescence markers should all be thoroughly investigated (26, 27).

Previous studies have revealed that the increase of heterogeneity, metabolic reconfiguration, and loss of cellular homeostasis could be potential reasons for BM-hMSC senescence during culture expansion (6, 11, 28–31). BM-hMSCs exhibit metabolic plasticity that can adapt themselves to artificial environments in order to maintain stem cell functions, specifically by reconfiguring energy production from glycolysis towards oxidative phosphorylation (OXPHOS), which contributes to the loss of mitochondrial fitness and replicative senescence (5, 11, 29, 30). Moreover, metabolism and stem cell fate are inherently intertwined to regulate immunomodulation in BM-hMSCs (5, 30, 32). For instance, BM-hMSCs establish glycolytic phenotype for immunosuppression *via* the indoleamine 2,3-dioxygenase (IDO)-prostaglandin E2 (PGE2) pathways after polarization by interferon (IFN)-γ. Pre-conditioning of BM-hMSCs in aggregation culture can reconfigure the metabolism and improve the production of anti-inflammatory factors (29, 33, 34). In addition, hMSC senescence is associated with the metabolic state in an artificial environment: the glycolytic phenotype facilitates the quiescence of BM-hMSCs and thus maintains their multipotency *in vivo*, while re-coupling with tricarboxylic acid cycle under *in vitro* culture conditions induces senescence (5, 28). Recently, the NAD+/NADH redox cycle and corresponding enzyme Sirtuins have been proposed to connect aging/senescence to cellular homeostasis (35–39). Our previous study revealed that the culture-induced alterations of NAD^+^/NADH redox balance contribute to metabolic alteration, mitochondrial dysfunction, loss of autophagy, and impaired stem cell functions in BM-hMSCs with replicative senescence (11). Other studies have documented similar findings for the role of NAD+ biosynthesis and metabolisms in age-related functional decline of stem cells (40, 41). However, the NAD+/NADH redox cycle, metabolic profiles, mitochondrial fitness, and corresponding metabolism have not been well investigated in hASCs during *in vitro* expansion.

In this study, the cellular behaviors of hASCs during *in vitro* culture expansion were fully characterized. The senescence, as well as senescence-associated metabolism and the NAD^+^/NADH redox cycle differed between hASCs and BM-hMSCs under similar culture conditions. Moreover, characterizations of hASCs at different passages indicated significant alterations of the cells’ immune phenotype from anti-inflammatory to pro-inflammatory after long-term culture, as well as the altered ability of inducing macrophage polarization. Transcriptomics and proteomics analysis of hASCs at different passages revealed changes in the cytokine/chemokine profiles during *in vitro* aging and potential pathways that regulate the shift in hASC immunophenotype. Our study provides novel information on the characteristics of hASCs during *in vitro* expansion and may offer guidance for large-scale production of hASCs in biomanufacturing and clinical applications.

## Materials and Methods

### hASC Cultures

Frozen hASCs at passage 1 were acquired from the Tulane Center for Stem Cell Research and Regenerative Medicine. The hASCs were isolated from the subcutaneous abdominal adipose tissue from three de-identified healthy donors that were younger than 45 years old with a body mass index lower than 25 (**Supplementary Table S1**) (31, 42). The isolated cells were characterized for their MSC properties through colony-forming unit (CFU) assays as well as tri-lineage differentiation potential (osteogenic, adipogenic, and chondrogenic differentiation) *in vitro*. The hASCs (1 × 10^6^ cells/ml/vial) were frozen in media containing α-MEM, 2 mM L-glutamine, 30% fetal bovine serum (FBS), and 10% dimethyl sulfoxide and were thawed and cultured following the method described in our previous publications (30, 43). Briefly, hASCs were seeded at a density of 1,500 cells/cm^2^ in 150 mm diameter Petri Dishes (Corning, Corning, NY, USA) in a standard 5% CO_2_ incubator. Cells were cultured in complete culture medium (CCM) containing αMEM (Life Technologies, Carlsbad, CA, USA) with 10% FBS (Atlanta Biologicals, Lawrenceville, GA, USA), sodium bicarbonate (1X, ThermoFisher Scientific), and 1% Penicillin/Streptomycin (ThermoFisher Scientific) undergoing media changes every 2–3 days. Cells were grown to 80% confluence and then harvested by incubation with 0.25% trypsin/ethylenediaminetetraacetic acid (EDTA) (Invitrogen, Grand Island, NY, USA) for no greater than 7 min. Harvested cells were sub-cultured up to passage 15 for characterizations. For low passage of hASCs, P4-P5 cells were used. For high passage of hASCs, P12-P14 cells were used in the experiments.

### Cell Number, CFU-F, SA-β-Gal Activity, and Glucose/Lactate Measurements

Cell number was determined by Quant-iT™ PicoGreen kit (Invitrogen, Grand Island, NY, USA). Briefly, cells were harvested, lysed over-night using proteinase K (VWR, Radnor, PA, USA), and stained with Picogreen to allow quantitation of cellular DNA. Fluorescence signals were read using a Fluror Count (PerkinElmer, Boston, MA, USA). The population doubling time (mean PD time) was determined through culture in each passage:

$$\text{MeanPDtime}=\frac{t}{\log_{2}n}$$

where *t* is culture time, *n* is the cell number fold increase during culture time t.

For CFU-F assay, hASCs were harvested and re-plated at the density of 15 cells/cm^2^ on 60 cm^2^ culture dish and cultured for another 14 days in CCM. Cells were then stained with 20% crystal violet solution in methanol for 15 min at room temperature (RT) and gently washed with phosphate-buffered saline (PBS) three times. The number of individual colonies were counted manually. Cellular senescence was evaluated by SA-β-Gal activity assay kit (Sigma, St. Louis, MO, USA) as described in manufacturer’s instructions. The intensity was normalized to cell number. Fresh and spent CCM were collected to determine glucose consumption and lactate production by YSI 2950 Biochemistry Select Analyzer (Yellow Spring, OH, USA).

### Intracellular NAD^+^ and NADH Quantification and ATP Measurements

Intracellular NAD^+^ and NADH were measured with NAD^+^/NADH Quantification Colorimetric Kit (BioVision, Milpitas, CA, USA) according to manufacturer’s instructions with some modifications. Briefly, approximate 0.8 million cells were collected and directly lysed in 200 µl lysis buffer from the assay kit. The volume of reagents in each step was scaling down by 50% and the results were calculated by the freshly prepared standard curve (NADH standards provided by the assay kit). Final NAD^+^ and NADH concentrations were then normalized to the total cell number in each group.

ATP measurements: hASCs were centrifuged, re-suspended in deionized water, and heated immediately in boiling water for 15 min. The mixture was centrifuged, and ATP-containing supernatant was collected. Upon measurement, 10 µl of ATP solution was mixed with 100 µl of the luciferin-luciferase reagent (Sigma-Aldrich), and the bioluminescent signal was measured using an Orion Microplate Luminometer (Bad Wildbad, Deutschland). To determine the glycolytic ATP ratio, cells were cultured with or without glycolysis inhibitor 2-Deoxy-D-glucose (2-DG, 5 mM) for 48 h and then the ATP was measured. The ratio of glycolytic ATP was calculated by the delta value of total ATP and 2-DG treated ATP normalized to total ATP.

### Measurement of IDO Activity

For IDO enzymatic activity, including both IDO1 and IDO2 (both convert Tryptophan to Kynurenine) was assessed by measuring Kynurenine level in cell culture supernatant. A 400 μl of supernatant from hASC culture (either stimulated by IFN-γ at 40 ng/ml or left unstimulated for 48 h until sample collection) was clarified by mixing with trichloroacetic acid (200 μl, 30% by weight; Sigma Aldrich, St. Louis, MO, USA) by vortex, followed by centrifugation at 8,000×g for 5 min. An equal volume of Ehrlich reagent (2% p-dimethylaminobenzaldehyde in glacial acetic acid) was added to the clarified supernatant, and optical density at 490 nm was measured. Rapamycin (Rapa, 100 nM) or mitoquinone (MitoQ, 1 µM) were used to treat cells together with IFN-γ stimulation.

### Real-Time Reverse Transcriptase-Polymerase Chain Reactions (RT-PCR)

Total RNA was isolated using the RNeasy Plus kit (Qiagen) following vendor’s instructions. Reverse transcription was carried out using 2 μg of total RNA, anchored oligo-dT primers (Operon), and Superscript III (Invitrogen). Primers for specific target genes were designed using the software Oligo Explorer 1.2 (Genelink) (**Supplementary Table S2**). β-actin was used as an endogenous control for normalization. RT-PCR reactions were performed on an ABI7500 instrument (Applied Biosystems), using SYBR Green PCR Master Mix. The amplification reactions were performed and the quality and primer specificity were verified. Fold variations in gene expressions were quantified using the comparative Ct method: 2^-Δ(CtTreatment -CtControl)^, which is based on the comparison of the target gene (normalized to β-actin) among different conditions.

### Flow Cytometry for Immune-Phenotype, Cell Cycle, and Autophagy

Cells were harvested with 0.25% trypsin-EDTA solution, washed in PBS, and then fixed in 4% paraformaldehyde (PFA) at RT for 15 min. Cells were then permeabilized in 0.2% triton X-100 for 10 min at RT. Non-specific binding sites were blocked with 1% bovine serum albumin and 10% FBS in PBS for 15 min at RT. After washing, cells were incubated with specific primary antibodies for human Sirt-1, Sirt-3, NAMPT, CD38, and CD73 (Santa Cruz Biotechnology, Dallas, TX, USA) at RT for 2 h, followed by incubation with FITC-conjugated secondary antibody (Molecular Probe). Labeled samples were acquired using BD FACSCanto II flow cytometer (Becton Dickinson) along with isotype control. The results were analyzed using FlowJo software.

For cell cycle analysis, suspended cells were fixed with 70% cold ethanol for 30 min at 4°C and then washed with PBS. RNase A (100 µg/ml, VWR, Radnor, PA, USA) was added to cell suspension and incubated at 35°C for 15 min. Then the samples were incubated with 400 µl 50 µg/ml of propidium iodide (VWR) solution at RT in the dark for 1 h. Cell cycle was then determined by flow cytometry.

For autophagy measurements, cell suspension was incubated with 20 µM Cyto-ID Green (Enzo Life Sciences, Farmingdale, NY, USA), a fluorescent dye that selectively labels accumulated autophagic vacuoles, at 37°C for 30 min, and analyzed by flow cytometry and calculated according to the manufacturer’s instructions.

### Mitochondrial Mass and Membrane Potential (MMP), and Reactive Oxygen Species (ROS)

For mitochondrial mass and MMP measurement, trypsinized hMSCs were washed in warm Hank’s Balanced Salt Solution (HBSS). The cell suspension was incubated with MitoTracker green FM or tetramethylrhodamine, methyl ester (Molecular Probe, Eugene, OR, USA) at 37°C for mass and MMP staining, respectively. Cells were then washed with HBSS, and analyzed by flow cytometry (BD Biosciences, San Jose, CA, USA).

For ROS measurement, cell suspension was incubated with 25 µM carboxy-H2DCFDA (Molecular Probe) at 37°C for 30 min and total ROS was determined using flow cytometry. For mitochondrial ROS measurement, cell suspension was incubated with 5 µM MitoSOX Red (Molecular Probe) at 37°C for 10 min and analyzed using flow cytometry.

### Western Blot Assay

Cells were lysed in radio-immunoprecipitation assay (RIPA) buffer (150 mM sodium chloride, 1.0% Trition X-100, 0.5% sodium deoxycholate, 0.1% sodium dodecyl sulfate, 50 mM Tris, pH 8, 2 µg/ml Aprotinin, 5 µg/ml Leupeptin, 5 µg/ml Antipain, 1 mM PMSF protease inhibitor), and homogenized by sonification using a Sonic Dismembrator 100 (Fisher Scientific, Hampton, NJ, USA). Samples were then digested for 20 min on ice, and spun down at 14,000 rpm for 20 min. The supernatant was collected and a Bradford assay was carried out to determine the protein concentration. Protein lysate concentration was normalized, and 20 µg of each sample was denatured at 95°C in 2× Laemmli Sample buffer. Proteins were separated by 15% BIS-Tris-SDS gels and transferred onto a nitrocellulose membrane (Bio-rad, Hercules, CA, USA). For the detection of non-phosphorylated proteins, the membranes were blocked for 30 min in 3% skim milk (w/v) in Tris-buffered saline (10 mM Tris-HCl, pH 7.5, and 150 mM NaCl) with 0.1% Tween 20 (v/v) (TBST), or in 3% bovine serum albumin in TBST. Membranes were incubated overnight in the presence of the primary antibody diluted in the corresponding blocking buffer at 4°C. Afterward, the membranes were washed four times for 10 min each with TBST and then incubated with an IR secondary (LI-COR, Lincoln, NE, USA) at 1:10,000 for 180 min at room temperature. Blots were washed another four times for 10 min each with TBST and processed using the LI-COR Odyssey (LI-COR). Images were analyzed using ImageJ software for band density, and the band density of proteins of interest was normalized to the band density of endogenous control α-tubulin.

### Characterization of Immunomodulatory Properties of hASCs

**(1) Macrophage differentiation in coculture with hASCs:** THP-1 cells were obtained from the American Type Culture Collection (ATTC) and cultured in ATCC-formulated RPMI-1640 until confluency. THP-1 cells were passaged into six-well plates at a concentration of 1 million cells/well. THP-1 cells were then differentiated into macrophages (M0) by a 48-h treatment of phorbol 12-myristate 13-acetate (PMA, 20 nM, Sigma) in ATCC-formulated RPMI-1640. M0 macrophages were then polarized into the M1 phenotype with 24-h treatment of lipopolysaccharide (LPS) (100 ng/ml, Sigma) and IFN-γ (50 ng/ml, Peprotech) in ATCC-formulated RPMI-1640 or the M2 phenotype with a 24-h treatment of IL-4 (10 ng/ml, Peprotech) in ATCC-formulated RPMI-1640. Co-culture was performed by allowing hASCs (hASC : THP-1 is at 1:10 ratio) to stabilize on the transwell insert for 6 h (in CCM) and then the insert was moved to the six-well plate containing polarized macrophages for another 48 h with either M1 stimulation or M2 stimulation.

**(2) The enzyme-linked immunosorbent assay (ELISA):** Secreted cytokines, i.e., CXCL10, IL-1β, IL-6, PGE2, IL-10, and hepatocyte growth factor (HGF), in cell culture supernatants was quantified using an ELISA Parameter Assay Kit (R&D Systems, Minneapolis, MN, USA) according to manufacturer’s instructions. The secreted cytokines were determined by subtracting cytokine concentrations in culture media controls and normalized to cell number.

### mRNA Extraction and mRNA-Seq cDNA Library Preparation

RNA was extracted from hASCs at passages 4, 8, and 12 (four biological replicates for each group) using the miRNeasy minikit (Qiagen). mRNA was isolated from the total RNA using an NEBNext Poly(A) mRNA Magnetic Isolation Module (New England Biolabs). cDNA libraries were generated from the isolated mRNA using an NEBNext Ultra RNA library prep kit for Illumina (New England Biolabs) and a unique six nucleotide index primer (NEBNext multiplex oligos for Illumina) was incorporated into each sample. The library construction was done according to the NEB manuals, modified for use with a Beckman Biomek 4000 at the Florida State University Biological Sciences core lab. The unique index (barcode) was added to each library to multiplex the six libraries in one lane of the sequencing run. The multiplexed sample was quantified with qPCR (Kapa Biosystems) specific for Illumina sequencing primers and the average fragment size was determined with a Bioanalyzer high sensitivity DNA chip (Agilent Technologies). Then 12 pM of the pooled sample was sequenced, with single end, 100 base reads on an Illumina HiSeq 2500 located in the Translational Science Laboratory at the College of Medicine, Florida State University. The pooled data were demultiplexed into individual sample data and adapter primer sequences were removed (44).

### mRNA-Sequencing Data Analysis

Initial quality control analysis of each sequenced library was performed using fastQC software (http://www.bioinformatics.babraham.ac.uk/projects/fastqc). The sequencing reads were further analyzed using RNA-Seq Alignment version 1.1.1 (Illumina BaseSpace application). The reads were aligned with Tophat 2 (45) to the human genome (genome release GRCh38) using default parameters and counts for each gene were generated. This workflow uses Cufflinks to generate FPKM (fragments per kilobase per million reads) normalized values (46). These normalized values account for differences in sequencing depth and the length of the gene. FPKM values were used to generate the heatmaps using Morpheus (Broad Institute; https://clue.io/morpheus). DESeq2 was used to determine statistically significant differentially expressed genes (a False Discovery Rate, FDR, of <0.05 was used) (47). The genes that were upregulated and downregulated among passage 4, passage 8, and passage 12 groups were further assessed for GO, KEGG pathway, and phenotype pathway analysis using Webgestalt (48, 49). The set of genes considered expressed in our dataset was used as the reference set to obtain significantly enriched pathways. Significant enrichment was determined in Webgestalt using the hypergeometric test and the Benjamini-Hochberg FDR method (50) for multiple testing adjustment.

### Proteomics Analysis

Cells were harvested at 80% confluency and hMSC pellets were resuspended in protein extracting buffer containing protease inhibitor. The samples were ultra-sonicated for 2 min on ice and the extracted protein concentration was determined by Bradford assay (Bio-Rad, Hercules, CA, USA). The proteins were then digested by modified Filter Aided Sample Prep (FASP) method 1. Briefly, 100 µg protein was vacuum-dried and resuspended in 8 M urea solution to a final volume of 200 µl, then 10 mM dithiothreitol (DTT) and 50 mM iodoacetamide (IAA) were added for reduction and alkylation respectively. Samples were transferred to a 10 kDa filter and centrifuged with 14,000 g for 30 min. After washing with 200 µl of 8 M urea and 200 µl of ammonium bicarbonate, the extracts were centrifuged at 14,000 g for 30 min. Then 2 µg trypsin was added for digestion at 37°C overnight. After that, peptides were collected and vacuum-dried.

An externally calibrated Thermo Q Exactive HF (high-resolution electrospray tandem mass spectrometer, MS, Thermo Scientific) was used in conjunction with Dionex UltiMate3000 RSLCnano System. The solution of 1 µg peptides in 0.1% formic acid was injected into a 50 μl loop and loaded onto the trap column (Thermo µ-Precolumn 5 mm, with nanoViper tubing 30 µm i.d. × 10 cm). The flow rate was set to 300 nl/min for separation on the analytical column (Acclaim pepmap RSLC 75 μM × 15 cm nanoviper). Mobile phase A was composed of 99.9% H_2_O (EMD Omni Solvent) with 0.1% formic acid and mobile phase B was composed of 99.9% acetonitrile with 0.1% formic acid. A 120 min-stepped gradient from 3 to 45% of phase B was performed. The LC eluent was directly nano-sprayed into Q Exactive HF MS. During the chromatographic separation, the Q Exactive HF was operated in a data-dependent mode and under direct control of the Thermo Excalibur 3.1.66 (Thermo Scientific). The MS data were acquired at 20 data-dependent collisional-induced-dissociation (CID) MS/MS scans per full scan (350 to 1700 m/z). The spray voltage for Thermo Scientific™ LTQ was 2.0 kV and the capillary temperature was set at 200°C. A survey full scan (m/z = 350–1,700) and the five most intense ions were selected for a zoom scan to determine the charge state, after which MS/MS was triggered in Pulsed-Q Dissociation mode (PQD) with minimum signal required (1,000), isolation width 2.0, normalized collision energy 27.0. All measurements were performed at room temperature. Raw files were analyzed by Maxquant 1.6 followed protein identification and relative comparison in Scaffold 4.4. Gene ontology (GO) annotation was carried out by WebGestalt while canonical pathway, diseases, and functions analysis was performed by Ingenuity Pathway Analysis (IPA) (Qiagen).

### Lineage-Specific Differentiations

**Osteogenic differentiation:** hMSCs were grown to confluence before CCM was switched to osteogenic differentiation medium containing high glucose DMEM (Gibco, Grand Island, NY, USA), 10% FBS, 1% penicillin/streptomycin, 100 mM dexamethasone, 10 mM sodium-β-glycerophosphate, and 0.05 mM ascorbic acid-2-phosphate. The media were changed every 2 days and the differentiation was maintained for 14 days. Cells were then collected for RT-PCR. The differentiation can be visualized by Von Kossa staining under microscope.

**Adipogenic differentiation:** hMSCs were grown to confluence before CCM was switched to adipogenic differentiation medium containing high glucose DMEM, 10% FBS, 1% penicillin/streptomycin, 0.2 mM indomethacin, 0.5 mM isobutyl-1-methyl xanthine, 1 µM dexamethasone, 10 µg/ml insulin, and 44 mM sodium bicarbonate. Medium was changed every 2 days and the differentiation was maintained for 14 days. Cells were then collected for RT-PCR. The differentiation can be visualized by Oil Red O staining under microscope.

### Statistical Analysis

Unless otherwise noted, all experiments were performed at least three times with triplicate biological samples (n = 3). The data from the representative experiments are reported. For mRNA-sequencing, four biological samples for each group were used (total 12 libraries) to ensure the statistical significance. For comparison of low and high passage cells, the cells from the same donor were compared to eliminate the donor variations. Experimental results are expressed as means ± standard deviation (SD) of the samples. Statistical comparisons were performed by one-way ANOVA and Tukey’s *post hoc* test for multiple comparisons, and significance was accepted at *p* < 0.05.

## Results

### Stem Cell Phenotype of hASCs During *In Vitro* Culture Expansion

**Figure 1:**
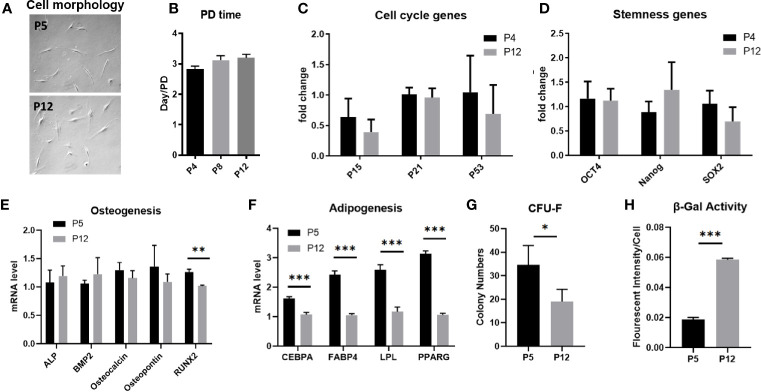
Stem cell properties of hASCs during *in vitro* culture expansion. **(A)** Representative images of hASC morphology during culture expansion. Scale bar: 250 µm. **(B)** The population doubling time of hASCs at different passages. **(C)** mRNA levels of cell cycle markers of hASCs at different passages. mRNA levels of **(D)** stemness genes and genes for **(E)** osteogenic differentiation and **(F)** adipogenic differentiation of hASCs at different passages. **(G)** Colony-forming ability (CFU) and **(H)** β-gal activity of hASCs at P5 and P12. *indicates *p* < 0.05; **p < 0.01; ***p < 0.001.

hASCs underwent limited cellular senescence following *in vitro* expansion from passages 4–5 (P4–P5) up to P12–P14 and exhibited morphological changes and alterations to specific functional properties. hASCs at early passage maintain a spindle-shaped morphology. However, throughout extended culture, hASC morphology flattens and the cells become elongated, losing their spindle shape (**Figure 1A**). hASCs subjected to extended *in vitro* culture had moderately increased population doubling times (although there was no statistical significance), maintaining a ratio of a population doubling roughly every 3 days (**Figure 1B**). However, the accumulative cell doublings for hASCs of aged donors in our previous publication revealed the prolonged doubling time (42). Similarly, mRNA levels encoding for *p53* and cyclin-dependent kinase inhibitor proteins (*p15* and *p21*) responsible for regulating cell cycle and cellular senescence show no difference between P4 and P12 cells (**Figure 1C** and **Supplementary Figure S1**). mRNA levels for genes that regulate stemness (*OCT4, Nanog*, and *SOX2*) showed no statistically significant differences between the two groups (**Figure 1D**). Following osteogenic differentiation of hASCs for 2 weeks, mRNA levels of osteogenic markers *ALP*, *BMP2*, *Osteocalcin*, and *Osteopontin* all showed no change in expression between P5 and P12 cells. However, Runx2, which is one of the most important transcription factors in early osteogenic differentiation was downregulated in P12 cells, indicating a loss in differentiation potential at the early stage of differentiation (**Figure 1E**). On the other hand, adipogenic differentiation of P12 hASCs all showed decreased mRNA levels for genes responsible for early differentiation regulation and late-stage maturation of adipocytes (*C/EBPα, FABP4, LPL*, and *PPARγ*) compared to the P5 group (**Figure 1F**). The representative images of hASC differentiation into adipocytes and osteoblasts were shown by Oil Red O staining and Von Kossa staining respectively (**Supplementary Figure S2**) and in our previous publication (42). hASCs’ functionality at high passages was also tested for CFU-F, which showed colony-forming ability decreased from about 35 colonies for P5 cells to less than 20 colonies for P12 cells (**Figure 1G** **)**. The increased SA-β-Gal activity indicates the increased levels of senescence in the P12 group compared to the P5 group (**Figure 1H**). Taken together, hASCs exhibited limited hallmarks of cellular senescence and maintained their proliferation rate under passage 15 during *in vitro* expansion. Thus, further characterizations (e.g., metabolism and immune phenotype) need to be considered for hASCs.

### Metabolism, NAD+/NADH Redox Cycle, and Sirt Activity During *In Vitro* Expansion

**Figure 2:**
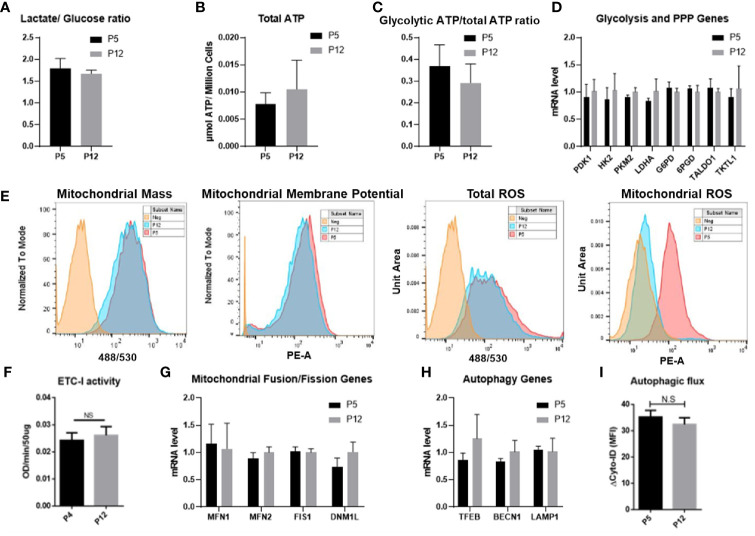
Metabolic characteristics of hASCs during *in vitro* culture expansion. Metabolic activity and mitochondrial fitness is relatively stable during *in vitro* culture expansion of hASCs. **(A)** Glucose consumption and lactate production is stable during culture expansion of hASCs. **(B)** Total ATP and **(C)** the ratio of glycolytic ATP to total ATP is well maintained during culture expansion of hASCs. The ratio of glycolytic ATP was calculated by the delta value of total ATP and 2-DG treated ATP normalized to total ATP. **(D)** Gene expression of critical enzymes involved in glycolysis and pentose phosphate pathway (PPP) of hASCs at different passages. **(E)** Mitochondrial fitness determined by flow cytometry: mass, Mitochondrial Membrane Potential (MMP), total reactive oxygen species (ROS), and mitochondrial ROS for P5 and P12 cells. **(F)** Electron transport complex-I (ETC-I) activity of hASCs at P4 and P12. **(G)** mRNA levels of genes involved in mitochondrial fusion and fission dynamics. **(H)** mRNA levels of genes related to autophagy. **(I)** Autophagic flux of hASCs at P5 and P12 *via* flow cytometry. Statistical analysis was performed and no statistical significance was observed.

Metabolic activity and mitochondrial fitness in senescent BM-hMSCs exhibited distinct characteristics and thus were evaluated for low passage and high passage hASCs during *in vitro* expansion. The mole ratio of consumed glucose and produced lactate was around 1.6–1.7 for both groups, indicating that hASCs are not exhibiting a shift in metabolic phenotype (**Figure 2A**). This is further confirmed by the comparable total ATP and glycolytic ATP ratios between the two groups (**Figures 2B, C**). The mRNA levels corresponding to critical enzymes in glycolysis and the pentose phosphate pathway (PPP) were not affected as well (**Figure 2D**). For the P5 and P12 hASCs, total ROS, mitochondrial mass, and membrane potential (i.e., MMP) remained consistent. Interestingly, mitochondrial ROS (mtROS) decreased in the P12 group compared to the P5 group (**Figure 2E**). Similarly, ETC-1 activity and mRNA levels of genes responsible for the regulation of mitochondrial fusion (*MFN1* and *MFN2*) and fission (*FIS1* and *DNM1L*) showed no statistically significant change (**Figures 2F, G**). hASCs through *in vitro* expansion also retain tight control over the degradation of damaged internal components, as shown by similar mRNA expression for genes related to lysosomal biogenesis and the regulation of autophagy (i.e., *TFEB*, *BECN1*, and *LAMP1*) (**Figure 2H**). The autophagic flux of hASCs at P4 and P12 determined *via* flow cytometry also showed comparable expression (**Figure 2I**).

**Figure 3:**
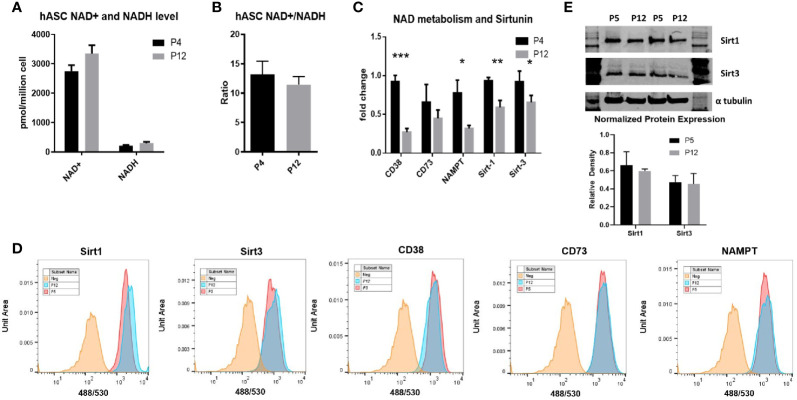
NAD+/NADH redox cycle and Sirt-1 activity in hASCs during *in vitro* culture expansion. **(A)** The levels of NAD+ and NADH, **(B)** the ratio of NAD+/NADH, and **(C)** mRNA expression of *CD38*, *CD73*, *NAMPT*, *Sirt-1*, and *Sirt-3* for P4 and P12 hASCs. **(D)** Flow cytometry histograms depicting little change in protein expression of Sirt1, Sirt3, CD38, CD73, and NAMPT. **(E)** Western Blot images and quantifications for the Sirt-1 and Sirt-3 enzymes normalized to α-tubulin. Data are shown in mean and standard deviation. *indicates *p* < 0.05; **p < 0.01; ***p < 0.001.

The NAD^+^/NADH redox cycle was investigated through extended *in vitro* culture of hASCs. Total moles of NAD^+^ and NADH normalized to cell numbers were similar between P4 and P12 groups, as was the ratio of NAD^+^/NADH between the low passage and high passage groups (**Figures 3A, B** **)**. However, the mRNA levels of main enzymes responsible for NAD+ depletion (*CD38*, *CD73*, *Sirt1*, and *Sirt3*), as well as the rate-limiting enzyme in the NAD^+^ salvage pathway (*NAMPT*), show a sharp decline in expression in the P12 hASCs compared to P4 cells (**Figure 3C**). Interestingly, the decreased mRNA expression does not correspond to the decreased levels of protein expression. The levels of protein expression of CD38, CD73, Sirt1, Sirt3, and NAMPT remained similar between P5 and P12 cells analyzed *via* flow cytometry (**Figure 3D**). Similar Sirt1 and Sirt3 protein expression was also confirmed *via* Western Blot (**Figure 3E**). Together, energy metabolism and NAD-Sirt pathways that associated with cellular senescence remained stable in hASCs during *in vitro* expansion.

### Immunomodulation Abilities of hASCs During *In Vitro* Expansion

**Figure 4:**
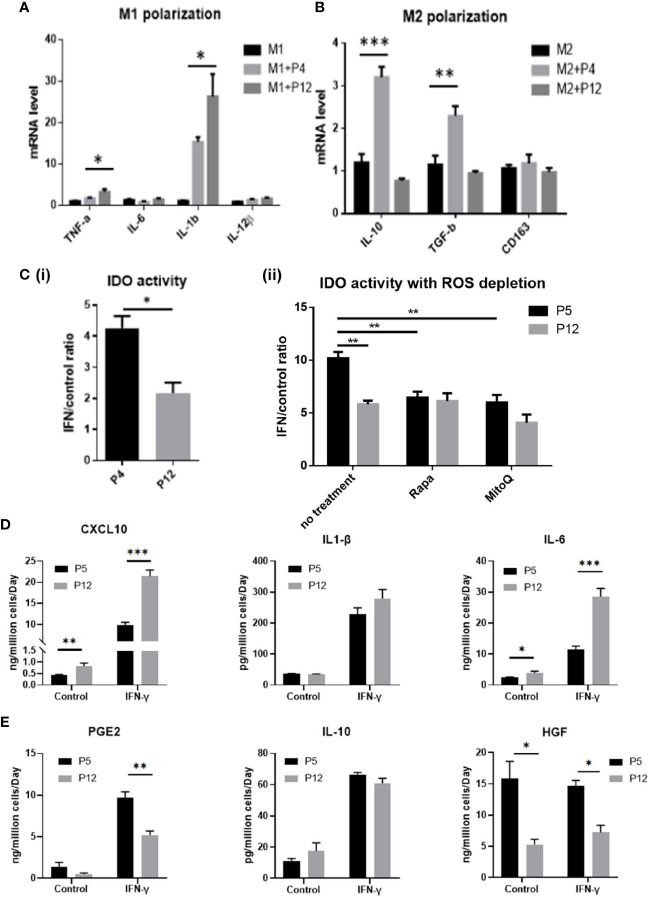
Immunomodulation ability of hASCs during *in vitro* culture expansion. Immunomodulatory potentials of hASCs was significantly changed during culture expansion. **(A)** mRNA levels for the genes involved in M1 macrophage polarization and **(B)** M2 macrophage polarization of macrophage co-cultured with hASCs from different passages. **(C)** (i) Indoleamine 2,3-dioxygenase (IDO) activity from basal level and interferon-gamma (IFN-γ) priming of hASCs at P4 and P12. (ii) IDO activity with reactive oxygen species (ROS) depletion. Rapamycin (Rapa, 100 nM) or mitoquinone (MitoQ, 1 µM) were used to treat hASCs together with IFN-γ stimulation. Secretion of **(D)** Pro-inflammatory cytokines (CXCL10, IL-1β, and IL-6); and **(E)** anti-inflammatory cytokines (PGE2, IL-10, and HGF) of hASCs at different passages (determined by ELISA assay normalized to cell number). *indicates *p* < 0.05; **p < 0.01; ***p < 0.001.

Although the metabolic phenotype of hASCs was shown to be stable through extended culture, stem cell functions with clinical relevance could be altered during *in vitro* expansion and this study focuses on the properties of hASCs that regulate macrophage phenotype and the IDO-PGE2 pathways that can modulate T cell responses (**Figure 4**). Prior to co-culture with hASCs, the M1/M2 macrophages were compared with M0 macrophages for mRNA expression of M1/M2 markers. The results indicate that the polarization is successful (**Supplementary Figure S3**). As the secretome of hASCs and macrophages cannot be separated in co-culture system, the mRNA levels of pro-inflammatory and anti-inflammatory cytokines of macrophages were determined. The P5 hASCs were shown to have minimal effect on the mRNA levels of M1 macrophages for pro-inflammatory cytokines, *TNF-α*, *IL-6*, and *IL-12β* (exception for *IL-1β*) when compared to the no co-culture control (**Figure 4A**). However, P12 hASCs increased the mRNA levels of *TNF-α* and *IL-1β* to a more pronounced degree than the P5 cells. By contrast, the P5 hASCs were shown to enhance mRNA levels of M2 macrophages for anti-inflammatory cytokines, *IL-10*, and *TGF-β*, while not affecting the expression of *CD163* (**Figure 4B**). Antithetically, P12 hASCs diminished the mRNA level for *IL-10* and had no effect on *TGF-β* and *CD163*. This indicates that the potential of hASCs to inhibit innate immune response is diminished by using P12 cells. Furthermore, the ratio of IDO activity for hASCs licensed with IFN-γ over the control decreased by two-fold for the P12 hASCs compared to the P5 group (**Figure 4Ci**). Furthermore, inhibition of mTOR *via* Rapamycin or depletion of mtROS *via* MitoQ in P5 hASCs significantly decreased the response to IFN-γ stimulation, though the influence on P12 hASCs is minimal (**Figure 4Cii**).

Analysis of spent media revealed that pro-inflammatory cytokines CXCL10 and IL-6 were secreted at a higher level for the P12 group before and after licensing with IFN-γ (IL-1β showed a non-statistically significant increase in the IFN-γ treated group) (**Figure 4D**). Contrarily, anti-inflammatory cytokines PGE2 (51) and HGF (52, 53) were secreted at higher levels for the P5 group when treated with IFN-γ (except for the IL-10) (**Figure 4E**). Fold change in cytokine secretion in response to IFN-γ was calculated to reveal immunomodulation potential of hASCs through extended culture (**Supplementary Figure S4**). Pro-inflammatory cytokines CXCL10 and IL-6 showed increase in fold change in P12 cells compared to P5 cells. Fold changes in anti-inflammatory cytokines gave mixed results with IL-10 decreasing, PGE2 comparable, and HGF increasing.

Relative mRNA expression of cell signaling genes (*PIK3CA*, *AKT*, *PTEN*, *mTOR*, and *NFKB*) for P12 *vs.* P4 hASCs were determined (**Supplementary Figure S5**). Except for *AKT*, the analyzed genes were higher in P4 cells compared to P12 cells, which may indicate that the signaling events related to phosphoinositide 3-kinases (PIK3), the mammalian target of rapamycin (mTOR), and nuclear factor-κB (NF-kB) pathways correlate to the increased pro-inflammatory phenotype in high passage (P12) hASCs.

### Global Alterations to the Transcriptome of hASCs During *In Vitro* Expansion

**Figure 5:**
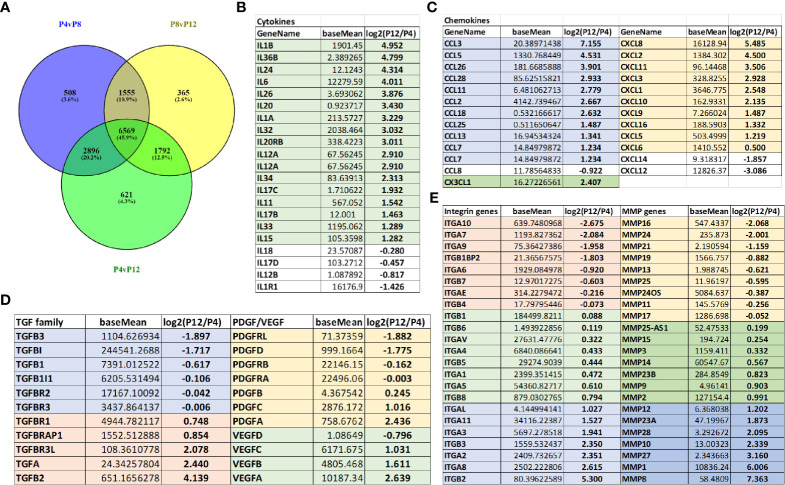
Global alterations of transcriptome in hASCs during *in vitro* culture expansion. **(A)** Venn diagram depicting variation and overlap of differentially expressed genes (DEGs) in P4, P8, and P12 hASCs from mRNA-sequencing transcriptome analysis. **(B)** Interleukin mRNA expression (shown in fold change) for P12 vs. P4 hASCs, showing upregulation at P12. **(C)** Chemokine mRNA expression (shown in fold change) for P12 vs. P4 hASCs, showing upregulation at P12. **(D)** Growth factor mRNA expression (TGF, PDGF, and VEGF) for P12 vs. P4 hASCs. **(E)** Integrin and matrix metalloproteinase (MMP) mRNA expression for P12 vs. P4 hASCs. The numbers are the Log2 values of ratios of P12 cells to P4 cells. Negative values indicate that the genes are present in higher amounts in the P4 group, while positive values indicate that the genes are present in higher amounts in the P12 group. The number 1 indicates two-fold increase.

Transcriptome analysis of P4, P8, and P12 hASCs was performed using next-generation sequencing of mRNA-Seq. The Venn diagram shows differentially expressed genes (DEGs) for P4 *vs.* P8, P4 *vs.* P12, and P8 *vs.* P12 comparisons (**Figure 5A**). For example, for P4 *vs.* P12 DEGs, among the total of 11,878 DEGs, 6,569 (45.9%) DEGs are commonly shared with P4 *vs.* P8 and P8 *vs.* P12; 2,896 DEGs (20.2%) are shared with P4 *vs.* P8 groups; 1,792 DEGs are shared with P8 *vs.* P12, and 621 DEGs (4.3%) are identified only for P4 *vs.* P12. Principle component analysis (PCA) showed a significant separation among P4, P8, and P12 conditions, and the four replicates exhibit a tight cluster for each group **(** **Supplementary Figure S6A**, **Supplementary Tables S1** and **S2**). Transcriptome analysis reveals that Interleukins are mainly upregulated in high passage P12 hASCs, and the top five candidates (*IL1- β*, *IL-36 β*, *IL-24*, *IL-6*, and *IL-26*) are linked to a pro-inflammatory response (**Figure 5B**). Similarly, chemokines upregulated in P12 hASCs are mainly associated with a pro-inflammatory response (e.g., *CCL3*, *CCL5*, *CCL26*, *CXCL2*, *CXCL8*) (**Figure 5C**). In particular, mRNA-sequencing data for genes encoding pro-inflammatory cytokines (CXCL10, IL-1β, and IL-6) showed upregulation for the cells of high passage numbers while anti-inflammatory cytokine (PGE2, IL-10, and HGF) genes all showed downregulation (**Table 1**). Importantly, p16ink4a is a checking point in senescence (54) and encoded by gene *CDKN2A* (55). The DEG value of *CDKN2A* is 1.100 (upregulated in P12 group) for P12 *vs.* P4, and 0.676 (upregulated in P8 group) for P8 *vs.* P4, from mRNA-sequencing data **Supplementary Table 1** and **2**). For *CDKN2B* (encoding p15), the DEG value is 0.856 and 1.554 respectively.

**Table 1:** mRNA-sequencing data for genes encoding pro-inflammatory cytokines (CXCL10, IL-1β, and IL-6) and anti-inflammatory cytokines (PGE2, IL-10, and HGF) of hASCs. (corresponding to **Figures 4D, E**).

Pro-inflammatory	Log (P12/P4)	Log (P8/P4)
IL1B	4.952	4.783
IL6	4.011	3.786
CXCL10	2.135	1.202
Anti-inflammatory	Log (P12/P4)	Log (P8/P4)
PTGER2	−0.550	−2.485
IL10	−2.197	−2.253
HGF	−1.732	−1.012

The numbers are the Log2 values of ratios of P12 or P8 cells to P4 cells. Negative values indicate that the genes are present in higher amounts in the P4 group, while positive values indicate that the genes are present in higher amounts in the P12 or P8 group. The number 1 indicates two-fold increase.

Since the target applications of the *in vitro* expanded hASCs is the treatment of neurological disorders such as stroke, Alzheimer’s disease, or multiple sclerosis, the genes related to brain pericytes, astrocytes, and microglia phenotype were examined (**Supplementary Figures S6B–E**) (44, 56, 57). The more primitive phenotype for brain pericytes and astrocytes was shown in P4 cells compared to P12 cells, and the microglia phenotype was more activated (i.e., pro-inflammatory) in P12 cells. Transforming growth factors (TGF), platelet-derived growth factors (PDGF), and vascular endothelial growth factors (VEGF) show differential responses with respect to extended *in vitro* culture (**Figure 5D**); in particular, VEGF-A was upregulated in the P12 group. Similarly, integrins and matrix metallopeptidases (MMPs) show a differential response with respect to *in vitro* culture expansion (**Figure 5E**). The genes for laminins, fibronectin, vitronectin, and chondroitin sulfate proteoglycans (CSPGs) were differentially expressed (**Supplementary Figure S6F**). Together, these changes may correspond to more pro-inflammatory phenotype of high passage hASCs. The genes related to CDKs were shown in **Supplementary Figure S7**. The CDK1 and CDK2 were upregulated in the P4 groups.

The genes related to the human leukocyte antigens (HLAs) and insulin-like growth factors (IGFs) were predominantly upregulated in P12 groups (**Supplementary Figures S8A, B** **).** The genes related to the bone morphogenetic protein (BMP) family and TNF family were differentially expressed (**Supplementary Figures S8C, D**). The genes in the Sirt family were similar for P4 and P12 cells (**Supplementary Figure S8E**). The genes for the HOXB family were differentially expressed (**Supplementary Figure S8F**), as well as different types of collagens (**Supplementary Figure S9A**). The genes related to metabolism were similar for P4 and P12 cells (**Supplementary Figure S9B**), which have been shown differently for 2D and 3D culture systems (58). Distinct CD markers upregulated in P4 cells or P12 cells were also identified for potential surface characterization of the cells (**Supplementary Figures S9C, D** **).** A similar analysis was performed for P4 *vs.* P8 hASCs, and in general, the aging trends were similar compared to P4 *vs.* P12 cell analysis (**Supplementary Figures S10–12** **).** The distinct DEGs for P4 *vs.* P8 and for P4 *vs.* P12 were shown in the heatmaps (**Supplementary Figures S13, S14** **).** Taken together, the data on the immunomodulatory properties of hASCs demonstrates a shift away from an anti-inflammatory phenotype to a pro-inflammatory phenotype during *in vitro* expansion, and the shift can be observed at P8 *vs.* P4 and persists in P12 cells.

### Global Alterations to the Proteome of hASCs During *In Vitro* Expansion

**Figure 6:**
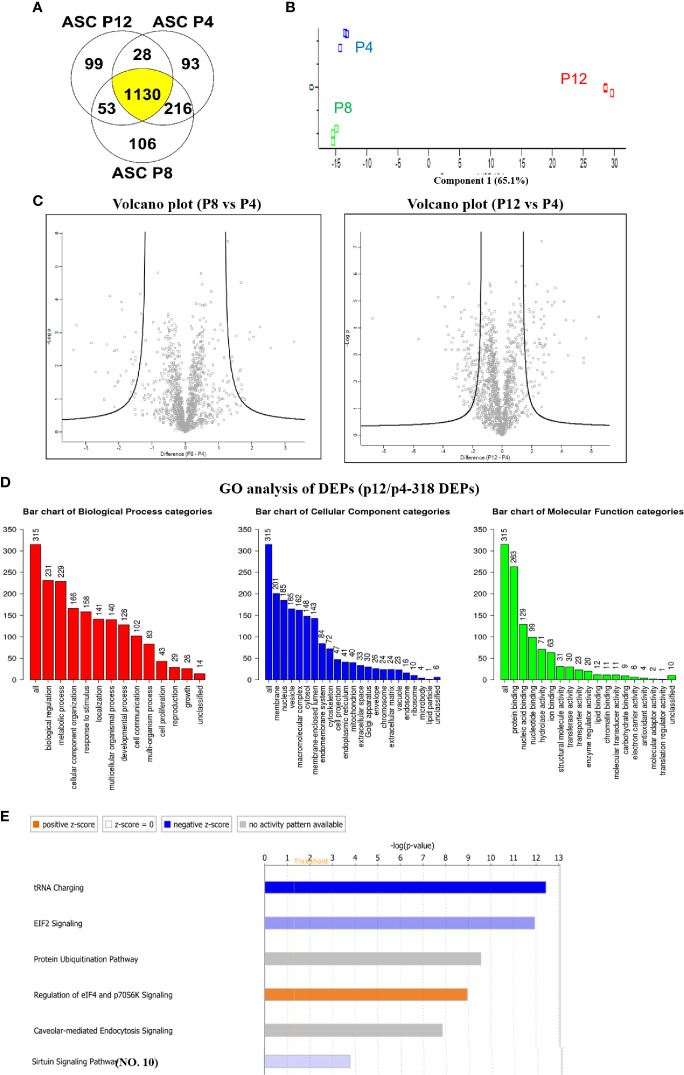
Global alterations of proteome in hASCs during *in vitro* culture expansion. **(A)** Venn diagram depicting variation and overlap of differentially expressed proteins (DEPs) in P4, P8, and P12 hASCs from proteomics analysis. **(B)** Principle component analysis (PCA) showing P4, P8, and P12 protein expression as three distinct groups. **(C)** Volcano plots depicting greater variation in protein fold change between the P4 *vs.* P12 group than the P4 *vs.* P8 group. **(D)** Gene ontology (GO) analysis for DEPs between P4 and P12 hASCs for biological processes, cellular components, and molecular functions. **(E)** Ingenuity Pathway Analysis (IPA) depicts signaling pathways with most significant evidence for alterations by *in vitro* passaging.

Proteomics analysis was performed for hASCs at P4, P8, and P12 following the workflow established in our lab **(** **Supplementary Figure S15** **)** (11, 31, 59). A total of 1,467, 1,505, and 1,310 different proteins were identified for P4, P8, and P12 groups, respectively. A total of 1,130 proteins (75.1%) overlapped among P4, P8, and P12 groups (**Figure 6A**). Next, the Benjamini-Hochberg procedure was utilized to define the cutoff components in principle component analysis (PCA), which in turn showed a significant separation among P4, P8, and P12 groups (**Figure 6B**). The triplicate samples were clustered together. Volcano plots of the proteomics data revealed the proteins with more than a 10-fold change for P8 *vs.* P4 and P12 *vs.* P4 cells (**Figure 6C**).

A total of 90 proteins were found differentially expressed between P8 and P4 groups, including 44 differentially expressed proteins (DEPs) that were upregulated and 46 DEPs that were downregulated (**Supplementary Figure S16** and **Supplementary Table S3**). GO annotation analysis demonstrated that metabolic process in the biological process categories, protein binding in molecular component categories, and protein binding in molecular function categories were the top categories correlating to the most DEPs. The pathway enrichment analysis illustrated that the Sirtuin signaling pathway, epithelial adherens junction signaling, fatty acid β-oxidation III, hepatic fibrosis/hepatic stellate cell activation, and interferon signaling were the top five pathways **(** **Supplementary Figure S17** and **Supplementary Table S4** **)**. Molecular function analysis by IPA showed the DEPs that were associated with enriched functions: cellular development, cellular growth and proliferation, cell morphology, cellular assembly and organization, cell death and survival are the top five categories. The upstream analysis showed the upstream regulator of these DEPs.

A total of 318 proteins were found differentially expressed between P12 and P4 groups, including 86 DEPs that were upregulated and 232 DEPs that were downregulated (**Supplementary Tables S5, S6** **)**. GO annotation analysis demonstrated that biological regulation in biological process categories, membrane in molecular component categories, and protein binding in molecular function categories were the top categories that most DEPs were correlated to (**Figure 6D**). The pathway enrichment analysis illustrates that tRNA charging, Eukaryotic Initiation Factor 2 (EIF2) signaling (31), protein ubiquitination pathway, regulation of eIF4 and p7056K signaling, and Caveolar-mediated endocytosis signaling were the top five identified pathways **(** **Figure 6E**).

Twenty-eight DEPs were overlapped with P8/P4 and P12/P4 comparisons, and 19 DEPs have the same changing trend between P8/P4 and P12/P4 comparisons (**Supplementary Table S7** **)**. GO annotation illustrated that metabolic process, membrane and protein binding were the top one item that most DEPs were correlated to **(** **Supplementary Figure S18** **)**. The pathway enrichment analysis showed that epithelial adherens junction signaling, actin cytoskeleton signaling, xanthine and xanthosine salvage, guanine and guanosine salvage I, and adenine and adenosine salvage I were the top five pathways. Function analysis by IPA demonstrates that the DEPs were associated with the key functions for cell morphology, cellular assembly and organization, carbohydrate metabolism, small molecule biochemistry, and post-translational modification.

## Discussion

Immunomodulation ability of hMSCs has been proposed as the major mechanism for endogenous tissue repair and regulation of immune response by coordinating the host immune system in disease progression. Recent studies have addressed the dual polarization of hMSCs *via* metabolic reconfigurations similar to T-cell polarization and the promising immunosuppressive effects of hMSCs in pre-clinical disease models (5, 30, 60, 61). hASCs are a promising candidate to replace BM-hMSCs for clinical usage as high cell yields can be obtained from adipose tissue with less-invasive liposuction procedures (62). hASCs also exhibit attractive therapeutic properties, such as multipotency and immunomodulation (3, 17, 20), and can be expanded for additional passages compared to BM-hMSCs (17, 63). Nonetheless, the therapeutic potentials of hASCs at high passage are in debate as no universal culture standards have been adapted, which could account for the inconsistent outcomes in different disease models (4, 5). In particular, the cellular characteristics of hASCs, such as metabolism, proteostasis, as well as functional changes during *in vitro* culture, have not been thoroughly investigated. This study revealed that hASCs exhibited limited hallmarks of senescence and maintained metabolic and redox stability during *in vitro* expansion while their immune phenotype shifts toward pro-inflammatory after rapid replication.

*In vitro* culture expansion increases hMSC’s heterogeneity and results in cellular senescence and the decline in primitive phenotype (28, 64). Indeed, the *in vivo* niches provide low oxygen to support the glycolytic phenotype and maintain stemness of hMSCs (65). Upon *in vitro* culture, BM-hMSCs reconfigure central energy metabolism and switch their metabolism towards OXPHOS to promote proliferation in the oxygen and nutrient-enriched environment. Generally, extensive OXPHOS leads to the exhaustion of mitochondria and accumulation of ROS, which can account for the replicative senescence (66, 67). BM-hMSCs have been shown to acquire culture-induced senescence with decreased proliferation rate, altered metabolism, and impaired mitochondrial functions (9, 11, 12). However, hASCs only exhibit certain characteristics of cellular senescence under the similar time-frame and culture conditions to BM-MSCs, as shown in this study. Our results showed that morphology, doubling times, expression of cell cycle, and stemness genes remained comparable for P4 and P12 hASCs. But the hASCs exhibited the declined CFU activity, the increased β-gal activity, and the upregulation of p16ink46a encoding gene (54). The active proliferation of hASCs at high passages may falsely lead to application of the cells in preclinical/clinical studies and compromise the therapeutic potentials as stem cell functions may be altered.

To further understand the mechanism behind the cellular senescence of hASCs, this study analyzed central metabolism, mitochondrial fitness, and the NAD+/NADH redox cycle associated with senescence demonstrated in our previous study for BM-hMSCs (11). As speculated, hASCs differ from BM-hMSCs by maintaining a similar metabolic phenotype at high passages in terms of glycolysis/OXPHOS status as well as mitochondrial fitness when compared to cells of low passages. Autophagy/mitophagy that control cellular homeostasis is also relatively active in hASCs after extensive expansion. Our previous study has identified that the NAD redox cycle-Sirt axis plays an essential role in regulating senescence/aging-associated functional decline in BM-hMSCs, indicated by the decreased NAD+ level and Sirt1/3 expression in high passage cells (11). However, for hASCs, the NAD/NADH redox cycle and the corresponding enzyme Sirt-1 remain unchanged after *in vitro* culture. Studies have tied the NAD redox cycle and Sirtuin enzyme family to *in vivo* aging and stem cell homeostasis (35–39). For hASCs, it is postulated that the cells hold more potential to maintain metabolic and redox homeostasis, thus are more resistant to culture stress compared to BM-hMSCs. The apparent proliferation ability may provide a “false hope” for the feasibility of clinical usage after large scale expansion of hASCs. However, the lack of metabolic reconfiguration in terms of glycolysis and mitochondrial fitness, together with stable proliferation and stemness, suggest an alternative mechanism modulating replicative senescence of hASCs during culture expansion.

As one of the essential characteristics of hMSCs in disease recovery and tissue engineering applications, the immunomodulatory potential and immune phenotype of culture-expanded cells should be evaluated prior to clinical application. Similar to T cells, the activation of BM-hMSC immunomodulation is attributed to the reconfiguration of central energy metabolism between glycolysis and OXPHOS to satisfy the high demand for biosynthesis of the immune-associated secretome (68). Although major carbon-associated metabolism pathways (glycolysis/OXPHOS, NAD+/NADH, autophagy/mitophagy, etc.) were maintained, the immune phenotype of hASCs significantly shifts from anti-inflammatory to pro-inflammatory after culture expansion. Furthermore, our results suggest immunomodulation abilities may be altered as high passage hASCs exhibit decreased fold secretion of IL-10 and increased fold secretion of CXCL10 and IL-6 when induced with IFN-γ to mimic an inflammatory environment. Comparison of RT-PCR results (e.g., Sirt, glycolytic genes) and the ELISA results (e.g., IL-10, HGF) with transcriptome analysis shows consistent trends in general. This overall shift in immune phenotype and potential impairment of immunomodulation indicates that further consideration is required for using *in vitro* expanded hASCs as therapeutic products, and the mechanism that regulates the immune phenotype may contribute to the cellular senescence of hASCs after prolonged *in vitro* expansion.

Besides metabolism regulation, hASCs’ immunomodulation could be controlled by other cellular components or signaling pathways. For instance, previous studies have demonstrated the regulatory role of mtROS in the activation of BM-hMSCs under IFN-γ priming (30). Our study utilized MitoQ to neutralize mitochondrial ROS which led to a reduction in IDO activity (69). mtROS is critical for hMSC immunomodulation *via* IDO-PGE2 pathway, which plays a critical role in glucose transportation (30). Though glycolysis/OXPHOS remains unchanged in hASCs during culture expansion, the immunomodulation/immune phenotype could be potentially regulated by mtROS, as P12 hASCs contained low mtROS as shown in this study. In addition, treatment with Rapamycin to inhibit mTOR signaling showed a similar effect corresponding to reductions in IDO activity in high passage hASCs. Correspondingly, our results showed a dramatic decrease in mtROS while no difference in total ROS at P12 hASCs compared to P5 cells. As mTOR activity leads to the downstream production of mtROS (70–72), our postulation is that the mTOR signaling pathway may still modulate the loss of immunomodulation potential observed at high passages of hASCs.

Moreover, global transcriptome and proteome analyses reveal several potential growth factor-regulated pathways (e.g., BMP, PDGFA, VEGFA, and EIF2) that regulate the immune phenotype in hASCs during culture expansion. In particular, our transcriptome data strongly supports the immune phenotype shift of hASCs during culture expansion, with the similar trends for P12 and P8 cells in comparison to P4 transcriptome, revealing insights into the growth factor regulations. These growth factors (i.e., BMPs, PDGFA, and VEGFA) have been reported to promote the proliferation and survival of multi-potential hMSCs (73). In addition, our previous study reveals that the EIF2 pathway regulation is common for BM-hMSCs and hASCs but not for somatic dermal fibroblasts (31). Moreover, differential expression of matrix proteins (collagens, laminins, fibronectin, and vitronectin), matrix remodeling proteins (MMPs), and integrins was also observed, indicating that additional functional analysis is required to reveal if the observed changes are causative or correlative.

There is evidence that other amino acid metabolic pathways and intermediate metabolites could regulate the phenotype of immune cells. For instance, succinate was found to accumulate in M1 polarized macrophages (74). Glutaminolysis is vital for both macrophage and T cell function besides aerobic glycolysis (75, 76). Re-routing of citrate and isocitrate into fatty acid plays an important role in dendritic cell activation (77). Similarly, rather than glycolysis and mitochondrial activity, other metabolism/metabolites could contribute to the altered immune phenotype of hASCs during *in vitro* expansion, as the metabolic process pathways stand out as the top altered biological processes from our proteomics analysis. However, further investigations are required to draw any conclusion.

Ultimately, the *in vitro* expansion system should be designed to slow down or reverse the cellular senescence process of hASCs. Our previous studies reveal that various methods can maintain the primitive phenotype of hMSCs and slow down the replicative senescence, including hypoxia (65, 78), 3D aggregate culture (6, 31, 34, 79), metabolic supplements (11, 29), and low-density culture (28). Together, the mechanism underpinning hASC immunomodulation under *in vitro* culture expansion should be further investigated to provide vital information for biomanufacturing of hASC-based therapeutic products.

## Conclusions

This study indicates that human adipose-derived MSCs exhibit limited senescence under *in vitro* expansion compared to human bone marrow MSCs. Moreover, metabolic reconfiguration and the NAD+-Sirt pathway are well maintained, supporting the limited replicative senescence in hASCs through extended *in vitro* expansion. However, the immune phenotype is significantly altered, indicated by the decreased secretion of anti-inflammatory cytokines and the increased secretion of pro-inflammatory cytokines during *in vitro* expansion. High passage hASCs also upregulate M1 macrophage polarization and inhibit M2 polarization. Transcriptome analysis also reveals the upregulation of pro-inflammatory genes and the shifts toward the pro-inflammatory phenotype after long-term expansion. This study suggests the importance of priming hASCs during biomanufacturing to maintain the desired therapeutic quality in hMSC-based therapy.

## Data Availability Statement

The datasets presented in this study can be found in online repositories. The names of the repository/repositories and accession number(s) can be found in the article/**Supplementary Material**.

## Author Contributions

RJ, XY, and YL conceived the experiments and wrote the manuscript. RJ and XY conducted the majority of the experiments. QF performed sample preparation and data analysis for proteomics results. TL and BB helped experimental design and reviewed the manuscript. RJ, XY, and YL analyzed results. All authors contributed to the article and approved the submitted version.

## Funding

This work was supported by National Science Foundation Award (CBET #1743426) and partially supported by the National Institutes of Health (NIH; R01NS102395). The content is solely the responsibility of the authors and does not necessarily represent the official views of the NIH.

## Conflict of Interest

The authors declare that the research was conducted in the absence of any commercial or financial relationships that could be construed as a potential conflict of interest.
