# Supplementary material for: In Vitro Culture Expansion Shifts the Immune Phenotype of Human Adipose-Derived Mesenchymal Stem Cells
Source: Front Immunol. 2021 Mar 10;12:621744. doi: 10.3389/fimmu.2021.621744 (PMC7988085; doi:10.3389/fimmu.2021.621744)
Supplement: Supplementary file 1 [file DataSheet_1.pdf]

Supplementary Materials

In Vitro Culture Expansion Shifts the Immune Phenotype of Human Adipose-derived Mesenchymal Stem Cells

Richard Jeske# [1], Xuegang Yuan#[1,2,*], Qin Fu[1,a], Bruce A. Bunnell[3], Timothy M. Logan[4], Yan Li[1,5,*]

1. Department of Chemical & Biomedical Engineering, FAMU-FSU College of Engineering, Florida State University, Tallahassee, FL, United States
2. The National High Magnetic Field Laboratory, Florida State University, Tallahassee, FL, United States
3. Department of Microbiology, Immunology, and Genetics, University of North Texas Health Science Center, Fort Worth, TX, United States
4. Department of Chemistry and Biochemistry, Florida State University, Tallahassee, FL, United States
5. Institute of Molecular Biophysics, Florida State University, Tallahassee, FL, United States

Supplementary Figure S1. Flow cytometry analysis of cell cycle distribution of hASCs at P5 and P12.

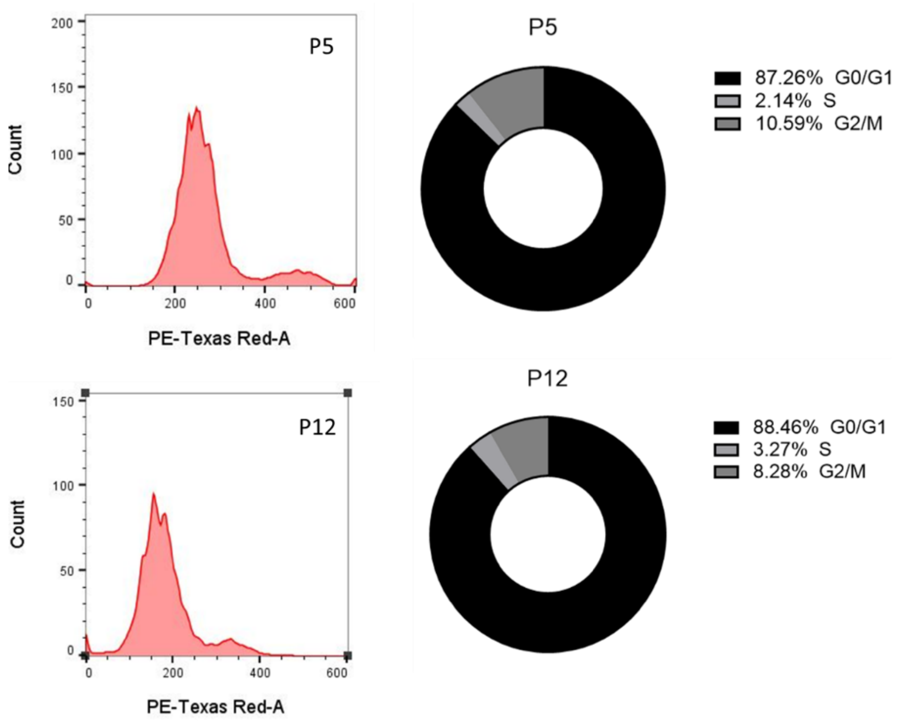

Supplementary Figure S2. Representative images for adipogenic differentiation and osteogenic differentiation. (A) Oil red O staining for adipogenic differentiation; and (B) Von Kossa staining for osteogenic differentiation. Scale bar: 100 μm.

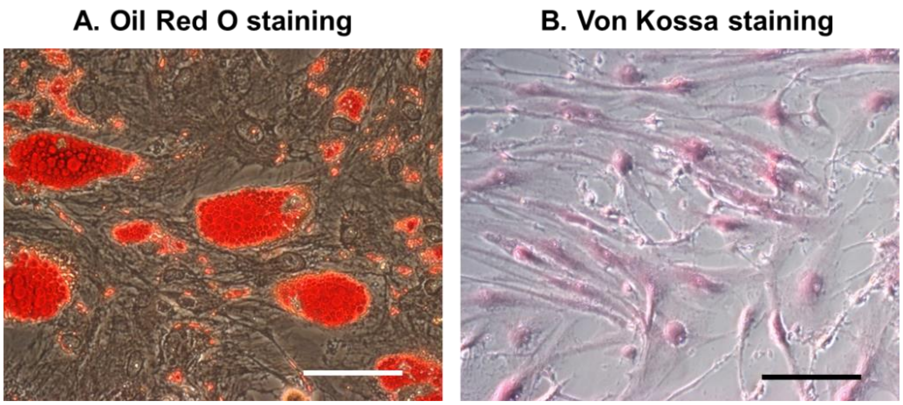

Supplementary Figure S3. Comparison of M1/M2 macrophages with M0 macrophages to confirm the successful polarization. The relative mRNA levels of M1 and M2 markers were determined by RT-PCR.

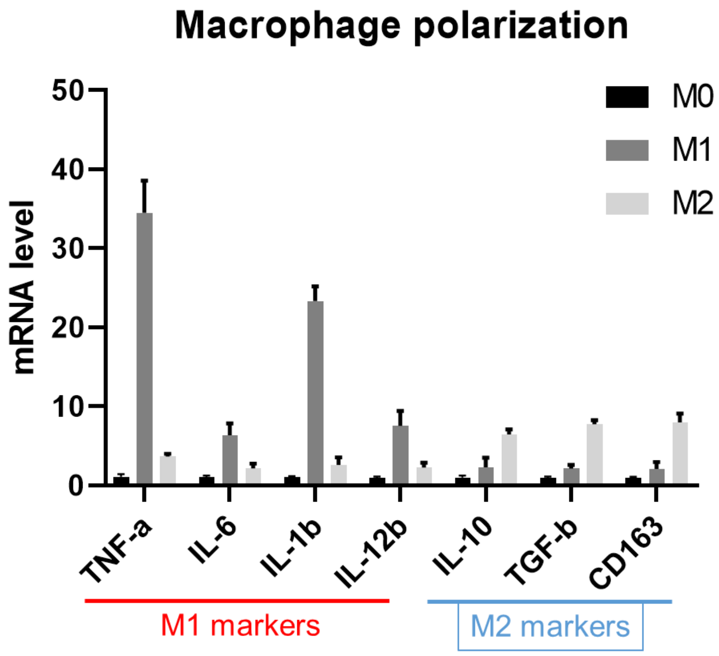

Supplementary Figure S4. The fold change in cytokine secretion when treated with IFN-γ compared to the control (without IFN-γ treatment) (corresponding to Figure 4D and 4E).

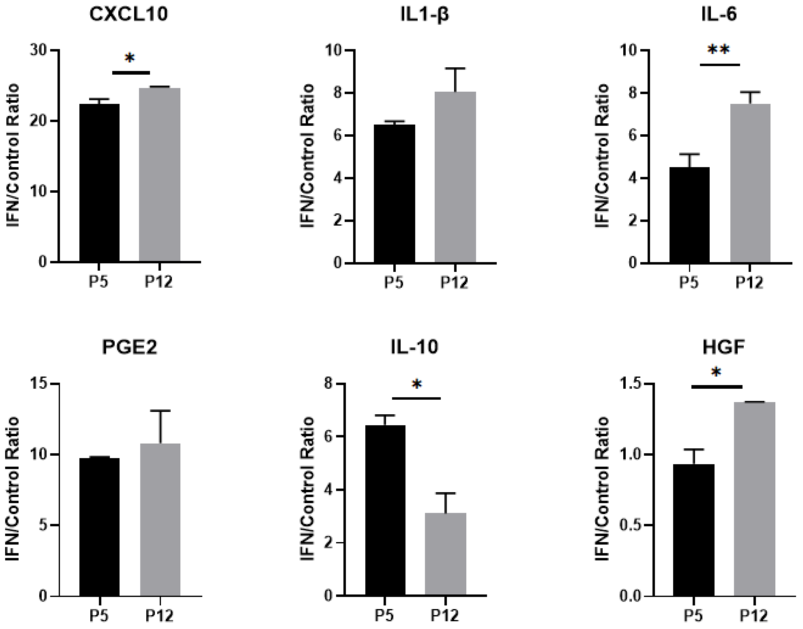

Supplementary Figure S5. Relative mRNA expression of cell signaling genes for P12 vs. P4 hASCs. * indicates $p<0.05$.

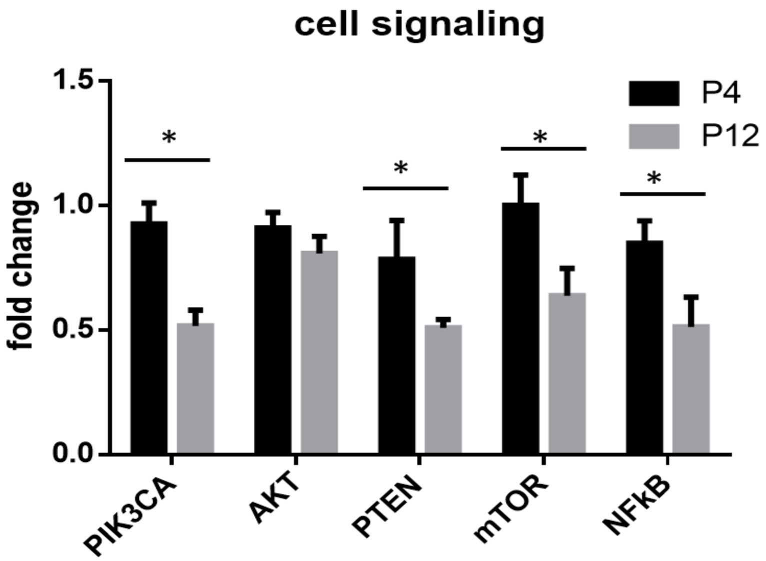

Supplementary Figure S6. Additional results from transcriptome analysis for P4 vs. P12 differentially expressed genes (DEGs). (A) Principal component analysis (PCA) demonstrated the separation of hMSCs at P4, P8, and P12 as categorized with the genes identified in transcriptomics. (B) Pericyte-related genes for P4 vs. P12 group. (C) Astrocyte-related genes for P4 vs. P12 group. (D) Microglia-related genes (highly expressed in P4 group or similarly expressed for P4 vs. P12 group). (E) Microglia-related genes (significantly upregulated in P12 group) for P4 vs. P12 group. (F). Extracellular matrix (ECM)-related genes differentially expressed in P4 and P12 groups. The numbers are the Log2 values of ratios of P12 cells to P4 cells. Negative values indicate that the genes are present in higher amounts in the P4 group, while positive values indicate that the genes are present in higher amounts in the P12 group. The number 1 indicates two-fold increase.

A

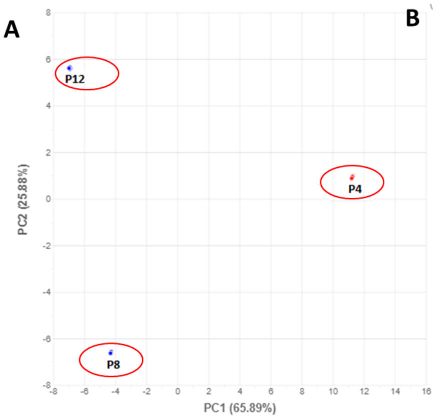

B

Pericyte genes	baseMean	log2(P12/P4)
ENG (CD105)	38723.145	**-1.241**
ABCC9	1561.067	**-0.895**
TBX18	1716.458697	**-0.882**
FOXF2	929.2241212	**-0.875**
THY1 (CD90)	40450.069	**-0.561**
KCNJ8	882.153	**-0.503**
ANPEP (CD13)	26186.308	**-0.470**
ZIC1	700.062	**-0.433**
PDGFRB	22146.14871	**-0.162**
TAGLN	3.102	**-0.122**
MCAM (CD146)	1242.046	**0.120**
FOXC1	977.156	**0.161**
VTN	138.6162746	**0.168**
ACTA2	23344.235	**0.346**
CNN1 (Calponin	4748.632	**0.373**
NT5E (CD73)	26038.684	**0.738**

C

Astrocyte gene	baseMean	log2(P12/P4)
S100B	0.811308246	**-3.046**
GFAP	12.90551414	**-1.974**
KCNJ10	2.332570528	**-1.753**
TNC	43632.6892	**-1.363**
NFIA	1339.162	**-0.888**
NFIB	2193.258	**-0.824**
NFIX	25634.959	**-0.714**
CD44	33709.79159	**-0.389**
SOX9	530.2990605	**-0.388**
VIM	267859.151	**-0.312**
RYR3	27.037	**0.116**
WIF1	0.256	**0.191**
SLC1A2	45.65872059	**0.228**
SLC1A3	348.615	**0.275**
GJA1	14403.97849	**1.153**
HEPACAM	0.661	**2.948**
ALDH1L1	1.414	**3.198**

D

Microglia genes	baseMean	log2(P12/P4)	Microglia genes	baseMean	log2(P12/P4)
ITM2A	48.865	**-4.371**	ENTPD1	682.088	**0.028**
TYROBP	1.074	**-3.440**	CD200R1	1.477	**0.325**
SPI1	1.092	**-1.592**	RGS10	666.143	**0.330**
OLFML3	10478.583	**-1.566**	GPR34	2.012	**0.436**
PROS1	956.090	**-1.495**	RUNX1	6437.255	**0.467**
TMEM119	9546.277	**-1.234**	ITM2B	19809.288	**0.476**
SELPLG	181.319	**-0.942**	SIRPA (CD172a)	8163.333	**0.574**
MERTK	4.970	**-0.935**	AIF1	0.139	**0.917**
CD74 (HLA-DR)	16.147	**-0.786**	P2RY14	6.258	**-5.993**
GAS6-AS1	8.779	**-0.782**	P2RY11	1.242	**-3.660**
CSF1R	26.666	**-0.703**	P2RY4	1.529	**-3.103**
PTPRC	8.483	**-0.648**	P2RY6	171.702	**-1.375**
ITGAM	0.356	**-0.512**	P2RY12	5.943	**-0.940**
CD68	5385.799	**-0.432**	P2RY2	14.502	**-0.392**
TLR3	263.816	**-0.145**	P2RY1	1.439	**3.210**

E

Microglia genes	baseMean	log2(P12/P4)
CSF1	10355.638	**0.989**
TLR4	1983.246	**1.169**
CTSD	25569.058	**1.328**
SIGLEC11	0.265	**1.639**
CX3CR1	3.259	**1.843**
CD274	128.116	**1.869**
IL34	83.639	**2.313**
GAS6	55906.156	**2.402**
CD14	136.674	**2.622**
TLR2	17.550	**3.770**
CSF3	89.908	**3.882**
CD200	130.574	**4.206**
CD163	53.477	**5.990**
CSF2	121.965	**10.471**
SIGLEC15	51.082	**0.439**
SIGLEC16	2.810	**1.003**
SIGLEC10	0.909	**1.542**
SIGLEC9	1.311	**3.066**

F

ECM gene	baseMean	log2(P12/P4)
LAMA2	8741.126955	**-0.931**
LAMC1-AS1	1.221379206	**-0.444**
LAMB4	1.688337388	**-0.404**
LAMA4	20597.24486	**-0.393**
LAMB1	23003.98907	**-0.333**
LAMB2P1	3.668089791	**-0.110**
LAMC1	107128.6094	**0.181**
LAMB2	42504.57215	**0.186**
LAMC3	0.74983529	**0.208**
LAMA1	1016.707871	**1.055**
LAMC2	1479.088708	**1.177**
LAMA5	2910.295926	**1.414**
LAMA3	187.7129631	**1.731**
LAMB3	792.1194837	**1.937**
FN1	3152597.472	**0.996**
VTN	138.6162746	**0.168**
CSPG4	4631.65873	**-1.215**
CSPG5	2.243898211	**0.494**

Supplementary Figure S7. The differential expression of CDK related genes. (A) P12 vs P4 comparison. (B) P8 vs. P4 comparison. The numbers are the Log2 values of ratios of P12 or P8 cells to P4 cells. Negative values indicate that the genes are present in higher amounts in the P4 group, while positive values indicate that the genes are present in higher amounts in the P12 or P8 group. The number 1 indicates two-fold increase.

A

CDKs	baseMean	log2(P12/P4)
**CDK1**	952.4750226	**-1.616**
**CDK2**	1525.840086	**-0.707**
**CDK5RAP3**	1885.393652	**-0.635**
**CDK5R2**	1.325657851	**-0.542**
**CDK15**	498.9790924	**-0.489**
**CDK11B**	2548.143836	**-0.416**
**CDK16**	6618.662043	**-0.317**
**CDK2AP1**	17654.9712	**-0.254**
**CDK10**	1171.946976	**-0.254**
**CDK12**	2850.469724	**-0.239**
**CDK9**	2128.767777	**-0.207**
**CDK5**	597.4168291	**-0.159**
**CDK19**	871.6929315	**-0.149**
**CDK5RAP2**	2192.176754	**-0.143**
**CDK8**	1027.590743	**-0.070**
**CDK5RAP1**	629.5394949	**-0.061**
**CDK13**	2321.256303	**0.065**
**CDK4**	3857.34302	**0.095**
**CDK7**	1295.525634	**0.232**
**CDK14**	3006.119183	**0.327**
**CDK18**	29.27011385	**0.405**
**CDK11A**	664.4656778	**0.439**
**CDK2AP2**	1609.455423	**0.756**
**CDK17**	2106.155631	**0.789**
**CDK20**	280.2641336	**0.849**
**CDK6**	4215.75467	**0.851**
**CDK5R1**	182.5135189	**0.971**

B

CDKs	baseMean	log2(P8/P4)
**CDK1**	864.92042	**-2.417**
**CDK2**	1309.3479	**-1.469**
**CDK5RAP3**	1848.4298	**-0.715**
**CDK10**	1085.902	**-0.558**
**CDK11B**	2592.4577	**-0.415**
**CDKL1**	148.88681	**-0.402**
**CDK2AP1**	17381.029	**-0.356**
**CDK11A**	517.57515	**-0.315**
**CDK5R2**	1.491666	**-0.257**
**CDK5RAP1**	615.27473	**-0.210**
**CDK5**	598.6013	**-0.199**
**CDK9**	2203.2784	**-0.148**
**CDK16**	7148.7493	**-0.130**
**CDK5RAP2**	2313.4319	**-0.041**
**CDK12**	3102.2201	**-0.035**
**CDK2AP2**	1238.0307	**-0.001**
**CDK4**	3807.1458	**0.012**
**CDK20**	208.24041	**0.064**
**CDK15**	611.80386	**0.095**
**CDK13**	2438.1369	**0.143**
**CDK5R1**	132.39873	**0.150**
**CDK7**	1294.5284	**0.188**
**CDK18**	27.933153	**0.233**
**CDK14**	3083.1923	**0.345**
**CDKAL1**	328.92719	**0.381**
**CDK8**	1234.3665	**0.383**
**CDK19**	1095.6397	**0.436**
**CDK17**	1910.6925	**0.519**
**CDKL5**	658.37766	**0.633**
**CDK6**	3907.5991	**0.635**
**CDKL3**	54.023247	**0.922**
**CDKL2**	2.1456202	**1.338**
**CDKL4**	1.8742296	**1.341**

Supplementary Figure S8. Additional results from transcriptome analysis for P4 vs. P12 DEGs. (A) Genes related to HLA molecules. (B) Genes related to IGF family; (C) Genes related to BMP family; (D) Genes related to TNF family; (E) Genes related to Sirt family; (F) Genes related to HOXB family.

The numbers are the Log2 values of ratios of P12 cells to P4 cells. Negative values indicate that the genes are present in higher amounts in the P4 group, while positive values indicate that the genes are present in higher amounts in the P12 group. The number 1 indicates two-fold increase.

A

HLA mole.	baseMean	log2(P12/P4)
**HLA-F-AS1**	43.77147	**-1.235**
**HLA-DOB**	6.647761	**-0.703**
**HLA-DPA1**	129.0556	**-0.569**
**HLA-DPB1**	122.2067	**-0.173**
**HLA-E**	16659.19	**0.119**
**HLA-DRB1**	1.758995	**0.608**
**HLA-DMA**	97.56198	**0.644**
**HLA-C**	39423.42	**1.051**
**HLA-F**	1141.597	**1.081**
**HLA-V**	6.257282	**1.237**
**HLA-A**	36981.53	**1.321**
**HLA-B**	33961.32	**1.797**
**HLA-L**	143.7689	**1.939**
**HLA-H**	2857.198	**2.685**
**HLA-DMB**	0.82848	**3.260**
**HLA-J**	15.39483	**3.611**
**HLA-G**	89.24683	**3.814**

B

IGF family	baseMean	log2(P12/P4)
**IGF1**	869.1093976	**-5.581**
**IGFLR1**	60.36906922	**-1.906**
**IGFBP4**	135072.1545	**-1.396**
**IGFBP6**	25133.20754	**-0.647**
**IGF1R**	4092.516875	**0.333**
**IGF2BP1**	975.3617673	**0.442**
**IGFBP3**	411339.0225	**0.442**
**IGF2BP2**	4113.9674	**0.774**
**IGF2R**	15376.79686	**0.976**
**IGFBP7-AS1**	4.305984467	**1.018**
**IGFBPL1**	164.863802	**1.066**
**IGFBP1**	13.173016	**1.202**
**IGFBP7**	54106.13298	**1.494**
**IGFBP5**	192195.6806	**1.564**
**IGF2**	1301.942909	**1.685**
**IGFN1**	14.16057657	**2.019**
**IGF2BP3**	394.7769587	**3.035**
**IGF2-AS**	3.029705344	**3.576**
**IGFBP2**	172.6736262	**4.185**

C

BMP family	baseMean	log2(P12/P4)
**BMP6**	2108.286	**-0.627**
**BMP8B**	3.640394	**-0.574**
**BMP8A**	18.27488	**-0.386**
**BMPR2**	6761.8	**-0.287**
**BMPR1A**	2000.509	**-0.114**
**BMP1**	8880.817	**-0.100**
**BMP2K**	901.6841	**0.192**
**BMPR1B**	244.0678	**0.759**
**BMP4**	155.0661	**1.179**
**BMP3**	3.675563	**1.288**
**BMPER**	4072.712	**1.721**
**BMP2**	70.67927	**2.199**

D

TNF family	baseMean	log2(P12/P4)
**TNFRSF18**	2.817383607	**-4.025**
**TNFRSF13C**	3.456440663	**-2.905**
**TNFRSF11A**	35.78336718	**-2.349**
**TNFRSF4**	0.6158154	**-1.538**
**TNFAIP8L2**	1.307279355	**-1.205**
**TNFRSF19**	653.8814426	**-1.118**
**TNFRSF25**	110.1173648	**-1.064**
**TNFAIP8L1**	263.6274254	**-0.993**
**TNFSF12-TNFSF13**	32.25616217	**-0.992**
**TNFSF13B**	120.2761215	**-0.943**
**TNFRSF1A**	9809.22317	**-0.528**
**TNFAIP8**	745.2096742	**-0.407**
**TNFSF12**	1061.483972	**-0.161**
**TNFRSF17**	0.498982471	**0.166**
**TNFRSF14**	451.7659646	**0.267**
**TNFRSF8**	40.66739948	**0.314**
**TNFSF13**	200.1672166	**0.368**
**TNFAIP2**	3741.234598	**0.417**
**TNFSF9**	575.2855043	**0.419**
**TNFRSF12A**	10860.94794	**0.562**
**TNFRSF10D**	1060.690427	**0.562**
**TNFRSF10C**	77.03143272	**0.684**
**TNF**	12.51162954	**0.812**
**TNFRSF9**	8.036096226	**1.260**
**TNFRSF10B**	5336.393181	**1.268**
**TNFRSF14-AS1**	27.38478175	**1.287**
**TNFSF15**	65.9010019	**1.384**
**TNFRSF1B**	509.8729528	**1.571**
**TNFRSF11B**	19315.97724	**1.749**
**TNFRSF6B**	250.5819342	**1.862**
**TNFSF14**	4.37867127	**1.872**
**TNFRSF21**	1048.459011	**2.070**
**TNFAIP3**	5979.853757	**2.158**
**TNFAIP6**	1212.057436	**2.448**
**TNFRSF10A**	185.6879216	**3.777**
**TNFSF8**	2.688279237	**4.205**
**TNFSF18**	2.947932439	**5.099**
**TNFSF4**	893.1020063	**5.473**

E

SIRT family	baseMean	log2(P12/P4)
**SIRT1**	1151.256524	**-0.163**
**SIRT7**	495.5490563	**-0.125**
**SIRT3**	617.401115	**-0.087**
**SIRT5**	356.3647251	**-0.079**
**SIRT6**	913.6728866	**0.037**
**SIRT2**	1896.887045	**0.066**
**SIRT4**	31.89167153	**0.630**

F

HOXB family	baseMean	log2(P12/P4)
**HOXB13**	79.27192014	**-4.333**
**HOXB2**	1850.519974	**0.380**
**HOXB3**	1338.926444	**0.595**
**HOXB4**	501.5388577	**0.568**
**HOXB5**	338.8508101	**1.042**
**HOXB6**	440.8451823	**0.797**
**HOXB7**	563.1165311	**1.341**
**HOXB8**	135.6717927	**3.748**
**HOXB9**	225.5430772	**2.932**

Supplementary Figure S9. Additional results from transcriptome analysis for P4 vs. P12 DEGs. (A) Genes related to collagen molecules. (B) Genes related to metabolism; (C) Genes related to CD markers; (D) Additional genes related to CD markers.

The numbers are the Log2 values of ratios of P12 cells to P4 cells. Negative values indicate that the genes are present in higher amounts in the P4 group, while positive values indicate that the genes are present in higher amounts in the P12 group. The number 1 indicates two-fold increase.

A

Collagens	baseMean	log2(P12/P4)	Collagens	baseMean	log2(P12/P4)
COL22A1	18.16699548	**-5.464**	COL12A1	161294.4	**0.237**
COL9A3	40.62497878	**-5.363**	COL6A1	268983.9	**0.310**
COL14A1	4195.637739	**-4.004**	COL6A2	239605.1	**0.410**
COL21A1	50.17505599	**-3.219**	COL4A5	113.8265	**0.696**
COL5A1-AS1	0.839783824	**-2.119**	COL18A1	3505.806	**0.759**
COL9A2	88.25097468	**-1.740**	COL4A2	72964.59	**0.911**
COL6A6	45.04364395	**-1.595**	COL2A1	1.400271	**1.040**
COL28A1	2.643228221	**-1.594**	COL4A1	64428.23	**1.130**
COL5A3	6343.384109	**-1.420**	COL7A1	1263.731	**1.367**
COL1A1	2862050.39	**-1.199**	COL11A1	13442.38	**1.510**
COL4A6	5.960923388	**-0.990**	COL13A1	1014.179	**1.515**
COL3A1	612010.0978	**-0.881**	COL8A2	2627.548	**1.731**
COL1A2	1383521.724	**-0.832**	COL10A1	35.26074	**2.021**
COL24A1	18.28113159	**-0.731**	COL15A1	3282.041	**2.039**
COL5A1	229666.6541	**-0.443**	COL4A3	11.02153	**2.277**
COL17A1	22.47471159	**-0.415**	COL8A1	14326.77	**2.395**
COL11A2	49.67347745	**-0.239**	COL23A1	0.530382	**2.628**
COL6A3	153382.6586	**0.041**	COL26A1	2.621529	**2.695**
COL16A1	44231.51486	**0.061**	COL4A4	142.2575	**2.844**
COL27A1	1239.143323	**0.163**	COL25A1	19.53168	**3.726**
COL5A2	97022.05758	**0.220**			

B

Metablism genes			
GeneName	log2(P12/P4)	GeneName	log2(P12/P4)
FASN	**-1.534**	MDH1	**0.206**
G6PD	**-1.155**	ACO1	**0.233**
IDH1	**-1.033**	SLC25A11	**0.239**
OGDH	**-0.831**	PKM	**0.271**
IDH2	**-0.647**	GAPDH	**0.394**
ACAA1	**-0.624**	ALDOC	**0.537**
SLC38A2	**-0.553**	ENO1	**0.543**
PDHA1	**-0.405**	GOT1	**0.560**
GOT2	**-0.361**	ALDOA	**0.562**
IDH3B	**-0.330**	GPI	**0.613**
SDHA	**-0.314**	LDHA	**0.699**
SLC25A1	**-0.161**	PGM1	**0.742**
ACO2	**-0.100**	PGAM1	**0.747**
FH	**-0.031**	GFPT1	**0.904**
GLUD1	**-0.013**	GFPT1	**0.904**
SUCLA2	**-0.012**	PGK1	**1.481**
PFKL	**0.176**	PDK1	**1.597**
SLC1A5	**0.194**	HK2	**1.996**
MDH2	**0.206**	SLC2A1 (GLUT1	**3.213**

C

CD Markers	baseMean	log2(P12/P4)	CD Markers	baseMean	log2(P12/P4)
CD24	74.361	**-4.515**	CD200R1	1.477	**0.325**
CD37	0.731	**-2.880**	CD47	3883.016	**0.468**
CD300C	3.335	**-2.381**	CD46	4054.915	**0.476**
CD93	5.272	**-1.770**	CD276	10594.922	**0.557**
CD81-AS1	11.515	**-1.620**	CD2AP	1768.409	**0.687**
CD248	68550.584	**-1.334**	CD36	5299.353	**0.717**
CD160	4.726	**-1.322**	CD302	911.640	**0.732**
CD38	49.629	**-0.930**	CD58	337.553	**1.011**
CD4	10.283	**-0.915**	CD19	1.768	**1.044**
CD74	16.147	**-0.786**	CD9	3190.723	**1.155**
CD96	3.936	**-0.716**	CD70	60.406	**1.340**
CD226	5.230	**-0.658**	CD72	22.843	**1.450**
CD320	1875.124	**-0.565**	CD163L1	79.339	**1.460**

D

CD79A	6.220	**-0.525**	CD40	66.642	**1.678**
CD99	27769.293	**-0.510**	CD80	2.742	**1.821**
CD109	9798.364	**-0.482**	CD274	128.116	**1.869**
CD27	7.266	**-0.446**	CD22	2.281	**1.888**
CD101	8.414	**-0.434**	CD55	3544.863	**1.956**
CD68	5385.799	**-0.432**	CD177	17.316	**2.167**
CD207	0.620	**-0.399**	CD14	136.674	**2.622**
CD44	33709.792	**-0.389**	CD28	3.585	**2.710**
CD34	328.603	**-0.251**	CD82	1140.020	**3.048**
CD59	53760.170	**-0.247**	CD83	87.724	**3.141**
CD81	33227.582	**-0.088**	CD200	130.574	**4.206**
CD63	43238.708	**-0.012**	CD163	53.477	**5.990**

Supplementary Figure S10. Corresponding to Figure 5: results from transcriptome analysis for P4 vs. P8 DEGs. (A) Interleukin mRNA expression (shown in fold change) for P8 vs. P4 hASCs, showing upregulation at P8. (B) Chemokine mRNA expression (shown in fold change) for P8 vs. P4 hASCs, showing upregulation at P8. (C) Integrin mRNA expression for P8 vs. P4 hASCs. (D) Growth factor mRNA expression (TGF, PDGF, and VEGF) for P8 vs. P4 hASCs. (E) Matrix metalloproteinase (MMP) mRNA expression for P8 vs. P4 hASCs.

The numbers are the Log2 values of ratios of P12 cells to P4 cells. Negative values indicate that the genes are present in higher amounts in the P4 group, while positive values indicate that the genes are present in higher amounts in the P12 group. The number 1 indicates two-fold increase.

A

Chemokines					
GeneName	baseMean	log2(P8/P4)	GeneName	baseMean	log2(P8/P4)
**CCL11**	27.307268	**4.980**	**CXCL2**	1885.990883	**4.934**
**CCL3L3**	2.3506537	**4.746**	**CXCL8**	5184.075441	**3.763**
**CCL5**	1391.541	**4.575**	**CXCL3**	429.7851775	**3.328**
**CCL3**	3.3570674	**4.489**	**CXCL11**	42.77240795	**2.142**
**CCL20**	42.763076	**3.569**	**CXCL1**	2490.328263	**1.845**
**CCL7**	25.718934	**2.234**	**CXCL16**	242.3919335	**1.790**
**CCL2**	3247.7936	**2.226**	**CXCL5**	554.2751933	**1.387**
**CCL28**	53.862767	**2.189**	**CXCL10**	101.1402488	**1.202**
**CCL26**	48.619602	**1.681**	**CXCL6**	1572.1913	**0.721**
**CX3CR1**	3.4016833	**1.885**	**CXCL12**	14091.81796	**-2.349**
			**CXCL14**	8.570047589	**-2.666**

B

Cytokines		
GeneName	baseMean	log2(P8/P4)
**IL24**	22.5438	**5.208**
**IL1B**	1724.7066	**4.783**
**IL20RB**	886.16843	**4.484**
**IL1A**	410.21221	**4.217**
**IL6**	10767.57	**3.786**
**IL32**	2447.4953	**3.311**
**IL26**	2.2146394	**3.035**
**IL11**	755.98735	**2.038**
**IL34**	57.657961	**1.604**
**IL17B**	12.123236	**1.449**
**IL15**	106.98297	**1.285**
**IL12A**	24.94199	**1.065**
**IL12A**	24.94199	**1.065**
**IL20**	-3914.228	**-0.210**
**IL1R1**	19556.444	**-0.658**
**IL17D**	92.80179	**-0.947**
**IL33**	484.2651	**-1.423**

C

Integrins		
GeneName	baseMean	log2(P8/P4)
ITGA10	633.4072812	**-2.913**
ITGA7	1143.990523	**-2.563**
ITGA9	74.49356566	**-2.172**
ITGA6	1795.1382	**-1.336**
ITGB1BP2	24.12172822	**-1.302**
ITGA8	513.1237266	**-1.195**
ITGA9-AS1	11.73507402	**-1.156**
ITGB3BP	231.7241597	**-0.996**
ITGAE	279.4912276	**-0.671**
ITGB1BP1	2042.304913	**-0.313**
ITGB1	172386.5645	**-0.162**
ITGB5	24092.539	**-0.136**
ITGA3	2506.707543	**0.129**
ITGA5	48071.30203	**0.264**
ITGA4	6691.113382	**0.330**
ITGA2	910.9906176	**0.346**
ITGA1	2565.508841	**0.597**
ITGB3	700.307449	**0.760**
ITGBL1	25898.49686	**0.940**
ITGB8	1065.606965	**1.177**
ITGA11	40755.38067	**1.836**
ITGB2	9.709580575	**1.917**

D

GeneName	baseMean	log2(P8/P4)
TGFB3	1090.357848	**-2.097**
TGFBR3	2721.716799	**-0.853**
TGFB1	7841.832536	**-0.488**
TGFB1I1	6120.548596	**-0.212**
TGFBI	369079.1314	**-0.095**
TGFBRAP1	1323.867685	**0.444**
TGFBR3L	66.62571074	**1.077**
TGFBR1	6267.961718	**1.227**
TGFB2	324.0595582	**3.019**
PDGFD	1239.98333	**-0.795**
PDGFRA	19866.81299	**-0.442**
PDGFRB	25818.21028	**0.229**
PDGFC	2409.816128	**0.578**
PDGFA	584.5553859	**1.938**
VEGFB	2945.252126	**0.521**
VEGFC	5767.382571	**0.838**
VEGFA	12736.36066	**2.978**

E

MMP family		
GeneName	baseMean	log2(P8/P4)
MMP16	604.94654	**-1.533**
MMP24	271.11627	**-1.274**
MMP3	742.92811	**-1.237**
MMP15	132.43375	**-1.123**
MMP11	136.35719	**-0.544**
MMP24OS	5224.9024	**-0.368**
MMP19	1833.5683	**-0.356**
MMP14	53104.542	**0.187**
MMP17	1463.3863	**0.232**
MMP2	113012.66	**0.689**
MMP1	467.22114	**0.816**
MMP24-AS1-EDI	61.500276	**0.897**
MMP25-AS1	71.926243	**0.915**
MMP23B	463.88045	**1.754**
MMP9	14.467134	**2.843**
MMP28	5.5421294	**2.987**
MMP8	4.9968554	**3.674**

Supplementary Figure S11. Additional results from transcriptome analysis for P4 vs. P8 DEGs). (A) Pericyte-related genes for P4 vs. P8 group. (B) Astrocyte-related genes for P4 vs. P8 group. (C) Genes related to IGF family; (D) Genes related to HLA molecules; (E) Microglia-related genes (highly expressed in P4 group or similarly expressed for P4 vs. P8 group). (F) Additional microglia-related genes (significantly upregulated in P8 group) for P4 vs. P8 group.

The numbers are the Log2 values of ratios of P12 cells to P4 cells. Negative values indicate that the genes are present in higher amounts in the P4 group, while positive values indicate that the genes are present in higher amounts in the P12 group. The number 1 indicates two-fold increase.

A

Pericyte genes	baseMean	log2(P8/P4
MCAM (CD146)	873.9260078	**-1.163**
KCNJ8	779.1072067	**-1.057**
ENG (CD105)	41156.06522	**-1.050**
TBX18	1881.090216	**-0.594**
ZIC1	683.1527067	**-0.587**
FOXF2	1040.353649	**-0.546**
VTN	115.0754843	**-0.435**
THY1 (CD90)	42730.91239	**-0.418**
ACTA2	19165.13889	**-0.277**
CNN1 (Calponin)	3896.575462	**-0.232**
ANPEP (CD13)	29006.30738	**-0.186**
ABCC9	2088.470909	**0.035**
TAGLN	132897.7755	**0.094**
PDGFRB	25818.21028	**0.229**
NT5E (CD73)	24614.90167	**0.560**
FOXC1	1448.645716	**1.047**

B

Astrocyte genes	baseMean	log2(P8/P4)
S100B	0.8278594	**-3.103**
KCNJ10	2.3269112	**-1.828**
GFAP	14.007791	**-1.411**
NFIA	1381.1178	**-0.835**
RYR3	21.324956	**-0.701**
VIM	247854.46	**-0.649**
WIF1	0.1193208	**-0.583**
CD44	33621.445	**-0.456**
SOX9	554.51307	**-0.312**
NFIX	29611.134	**-0.283**
SLC1A2	44.799152	**0.110**
SLC1A3	339.3898	**0.193**
NFIB	3120.127	**0.246**
TNC	75310.774	**0.445**
GJA1	13521.622	**0.980**
ALDH1L1	0.5035779	**1.435**
HEPACAM	0.3943449	**2.169**

C

IGF family	baseMean	log2(P8/P4)
IGFN1	3.1147434	**-3.438**
IGF1	950.29354	**-2.828**
IGFBP4	138000.82	**-1.398**
IGFLR1	72.843573	**-0.932**
IGFBP6	32914.736	**0.138**
IGF1R	4109.8911	**0.299**
IGF2BP1	940.45655	**0.306**
IGF2BP3	100.30526	**0.373**
IGF2R	13661.302	**0.672**
IGF2BP2	4229.401	**0.800**
IGFBP7	41457.545	**0.895**
IGFBP3	560598.41	**1.106**
IGFBP5	171968.46	**1.311**
IGF2	1151.01	**1.401**
IGFBP2	42.736512	**1.865**

D

HLA family	baseMean	log2(P8/P4)
HLA-F-AS1	46.508011	**-0.554**
HLA-E	19274.416	**0.460**
HLA-G	25.908068	**1.726**
HLA-F	1663.3242	**1.789**
HLA-A	49359.42	**1.840**
HLA-C	62319.115	**1.908**
HLA-L	149.72705	**1.981**
HLA-H	2167.05	**2.178**
HLA-B	48721.491	**2.404**
HLA-V	12.611372	**2.497**
HLA-J	33.145076	**4.750**

E

Microglia genes	baseMean	log2(P8/P4)	Microglia genes	baseMean	log2(P8/P4)
SPI1	0.8467581	**-3.128**	TLR3	302.6554057	**0.200**
ITM2A	60.124383	**-2.063**	GPR34	1.940852482	**0.329**
TYROBP	1.3654746	**-1.989**	ITM2B	20646.43105	**0.539**
GAS6-AS1	7.2532602	**-1.828**	CD74 (HLA-DR)	25.99002444	**0.616**
ITGAM	0.2285244	**-1.284**	CD200R1	1.846806044	**0.625**
MERTK	4.8751891	**-1.092**	RUNX1	7207.822949	**0.682**
OLFML3	11923.391	**-0.997**	SIRPA (CD172a)	9762.472758	**0.950**
CSF1R	27.002647	**-0.712**	AIF1	0.000	**NA**
PROS1	1166.4418	**-0.678**	P2RY14	6.701	**-4.650**
SELPLG	211.69131	**-0.415**	P2RY11	1.270	**-3.720**
TMEM119	12249.937	**-0.334**	P2RY4	1.558	**-3.156**
ENTPD1	616.30073	**-0.330**	P2RY6	170.318	**-1.536**
CD68	5812.4687	**-0.228**	P2RY2	12.924	**-0.883**
PTPRC	9.9719904	**-0.158**	P2RY12	6.403	**-0.729**
RGS10	631.9165	**0.146**	P2RY1	0.749	**2.125**

F

Microglia genes	baseMean	log2(P8/P4
TLR4	1425.179	**0.373**
CSF1	8806.659	**0.570**
CTSD	18969.61	**0.637**
GAS6	26293.38	**0.923**
CD274	88.93981	**1.094**
IL34	57.65796	**1.604**
SIGLEC11	0.275384	**1.626**
TLR2	5.372007	**1.764**
CX3CR1	3.401683	**1.885**
CD14	112.1942	**2.277**
CSF3	50.5019	**2.943**
CD200	67.63061	**3.155**
CD163	18.056	**4.356**
CSF2	7.801062	**6.479**
SIGLEC15	26.81242	**-2.294**
SIGLEC16	2.572308	**0.755**
SIGLEC10	0.377671	**-0.567**
SIGLEC9	0.250838	**0.140**

Supplementary Figure S12. Additional results from transcriptome analysis for P4 vs. P8 DEGs). (A) Collagen-related genes for P4 vs. P8 group. (B) Laminin-related genes for P4 vs. P8 group. (C) Genes related to metabolism (no significant change); (D) Genes related to HLA molecules; (E) Genes related to CD markers for P4 vs. P8 group. (F) Additional genes related to CD markers for P4 vs. P8 group.

The numbers are the Log2 values of ratios of P12 cells to P4 cells. Negative values indicate that the genes are present in higher amounts in the P4 group, while positive values indicate that the genes are present in higher amounts in the P12 group. The number 1 indicates two-fold increase.

A

Collagen genes	baseMean	log2(P8/P4)
COL22A1	19.02668966	**-4.290**
COL28A1	2.15654904	**-3.670**
COL17A1	14.47717403	**-3.182**
COL6A6	39.05414512	**-2.885**
COL21A1	61.78972475	**-1.975**
COL9A3	53.37389936	**-1.716**
COL14A1	5330.296083	**-1.612**
COLEC12	3251.779381	**-1.496**
COL9A2	95.30564992	**-1.446**
COLGALT1	16081.32484	**-0.490**
COL5A3	8683.017178	**-0.254**
COLGALT2	276.7657438	**0.253**
COL27A1	1348.309875	**0.331**
COL18A1	3090.377486	**0.360**
COL1A2	2078111.224	**0.387**
COL1A1	4702004.515	**0.389**
COL6A1	285002.3357	**0.403**
COL6A2	248978.9651	**0.445**
COL5A1	330811.9863	**0.532**
COL12A1	186271.7967	**0.559**
COL6A3	198067.6591	**0.647**
COL16A1	58461.76716	**0.717**
COL3A1	1105710.498	**0.803**
COL5A2	133287.175	**0.948**
COL4A2	77603.23522	**1.003**
COLEC10	19.99988239	**1.269**
COL4A1	71583.32838	**1.315**
COL11A1	12369.87606	**1.315**
COL8A2	2251.718871	**1.389**
COL4A4	67.26282092	**1.491**
COL7A1	1380.109014	**1.497**
COL10A1	27.49548904	**1.529**
COL1A2-AS1	12.24391896	**1.610**
COL8A1	15809.3176	**2.534**
COL15A1	5607.191027	**2.922**
COL25A1	11.7449788	**2.936**

B

Laminins		
**ECM gene**	**baseMean**	**log2(P8/P4)**
LAMA2	10409.852	**-0.348**
LAMB4	1.9424711	**-0.240**
LAMA5	1562.7417	**-0.124**
LAMA1	684.37834	**0.056**
LAMB2P1	3.9517584	**0.062**
LAMA4	24891.186	**0.111**
LAMC2	1015.8622	**0.262**
LAMB2	45049.492	**0.301**
LAMC1	117365.62	**0.375**
LAMB3	390.67252	**0.426**
LAMB1	30549.548	**0.427**
LAMC3	0.8789969	**0.575**
LAMA3	159.53939	**1.364**
FN1	3336999.1	**1.073**
VTN	115.07548	**-0.435**
CSPG4	4073.0969	**-2.099**
CSPG5	1.6029233	**-0.551**

C

Metabolism			
**GeneName**	**log2(P8/P4)**	**GeneName**	**log2(P8/P4)**
FASN	**-1.713**	PKM	**0.008**
OGDH	**-1.104**	SUCLA2	**0.018**
G6PD	**-0.980**	PFKL	**0.260**
IDH2	**-0.747**	ALDOC	**0.311**
SLC38A2	**-0.544**	GAPDH	**0.372**
IDH1	**-0.534**	GPI	**0.419**
IDH3B	**-0.502**	LDHA	**0.502**
PDHA1	**-0.489**	ALDOA	**0.555**
SDHA	**-0.465**	PGM1	**0.580**
SLC25A1	**-0.458**	GFPT1	**0.589**
GOT2	**-0.396**	PGAM1	**0.751**
ACO1	**-0.385**	ENO1	**0.769**
MDH2	**-0.317**	GOT1	**1.039**
ACAA1	**-0.276**	SLC1A5	**1.262**
ACO2	**-0.226**	PGK1	**1.505**
GLUD1	**-0.198**	HK2	**1.711**
MDH1	**-0.146**	PDK1	**2.089**
SLC25A11	**-0.055**	SLC2A1 (GLUT1	**3.090**

D

CD Marker	baseMean	log2(P8/P4)	CD Marker	baseMean	log2(P8/P4)
CD24	78.000524	**-4.670**	CD27	8.594487665	**0.031**
CD37	0.8846086	**-2.208**	CD81	35543.51401	**0.056**
CD1D	0.8738455	**-2.070**	CD27-AS1	375.023374	**0.075**
CD160	5.0522087	**-1.802**	CD47	3483.289107	**0.139**
CD22	0.6067068	**-1.558**	CD7	7.32564829	**0.146**
CD93	5.9327982	**-1.201**	CD46	3672.215982	**0.179**
CD4	9.9702786	**-1.031**	CD164	15683.86198	**0.231**
CD36	2959.4987	**-0.848**	CD226	7.311187496	**0.254**
CD248	82408.995	**-0.638**	CD2AP	1583.141664	**0.379**
CD59	48905.449	**-0.625**	CD70	42.09056305	**0.425**
CD300C	4.6817323	**-0.622**	CD302	830.211304	**0.463**
CD101	8.1601247	**-0.572**	CD83	22.10610543	**0.481**
CD81-AS1	14.948405	**-0.541**	CD58	275.9994606	**0.507**
CD109	9943.3047	**-0.489**	CD74	25.99002444	**0.616**
CD44	33621.445	**-0.456**	CD200R1	1.846806044	**0.625**
CD9	1748.9165	**-0.432**	CD96	6.120009505	**0.696**
CD40	28.185198	**-0.407**	CD33	1.101263924	**0.930**

E

CD320	2006.7097	**-0.403**	CD72	18.9656551	**1.037**
CD79A	6.4168097	**-0.400**	CD274	88.93980647	**1.094**
CD3EAP	744.93362	**-0.392**	CD55	2308.998904	**1.123**
CD151	23550.369	**-0.248**	CD34	588.6512124	**1.169**
CD68	5812.4687	**-0.228**	CD163L1	81.10102244	**1.472**
CD63	41303.909	**-0.203**	CD38	129.0682795	**1.538**
CD2BP2	2912.9015	**-0.195**	CD177	12.65214833	**1.563**
CD19	1.1106145	**-0.160**	CD82	531.3420725	**1.702**
CD276	8370.6867	**-0.125**	CD79B	1.162489724	**1.759**
CD99	32663.45	**-0.043**	CD28	2.504790373	**2.078**
			CD14	112.194181	**2.277**
			CD200	67.63061132	**3.155**
			CD80	7.499752035	**3.502**
			CD163	18.05600314	**4.356**

Supplementary Figure S13. Heatmaps for DEGs of P4 vs. P8 only in Venn Diagram.

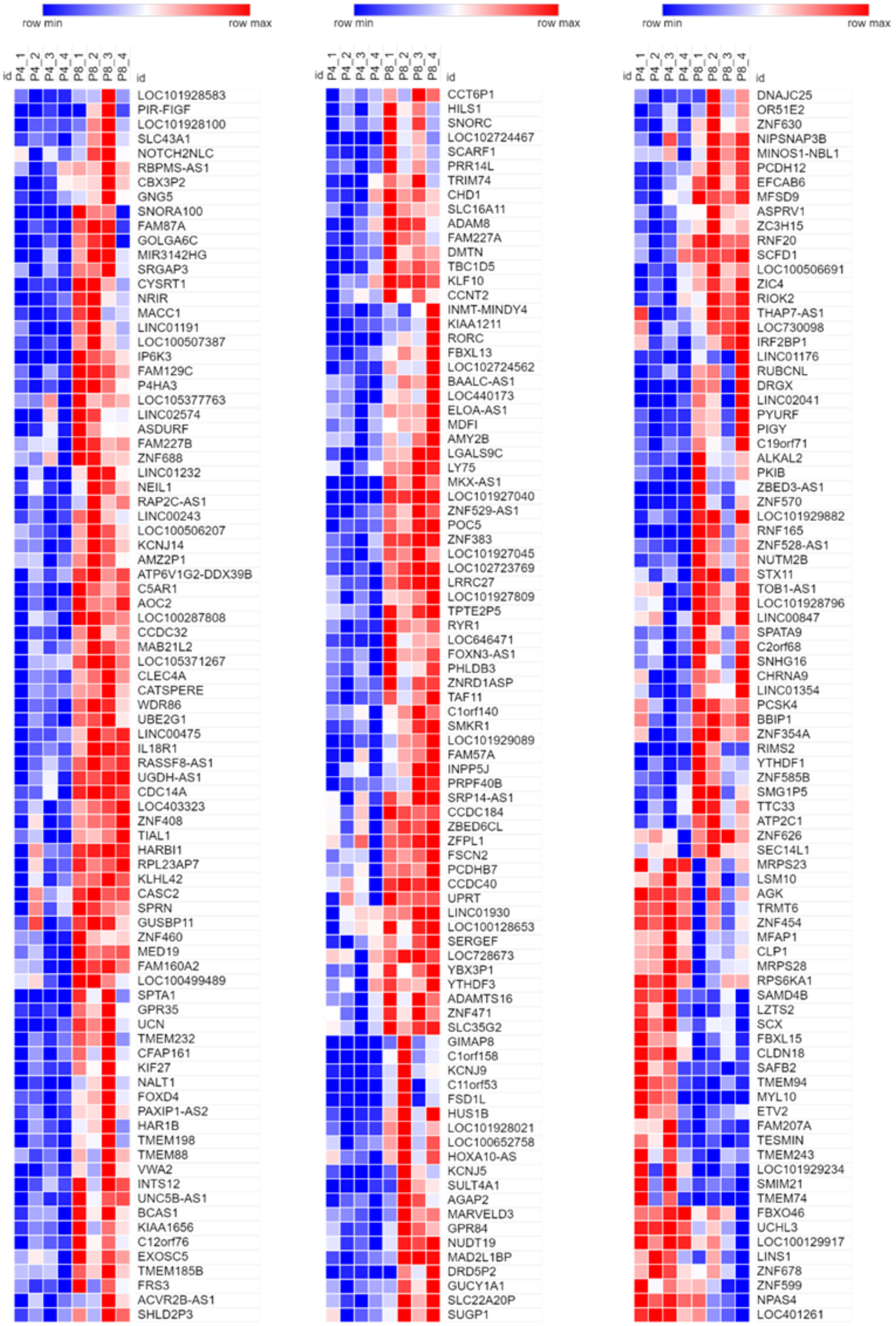

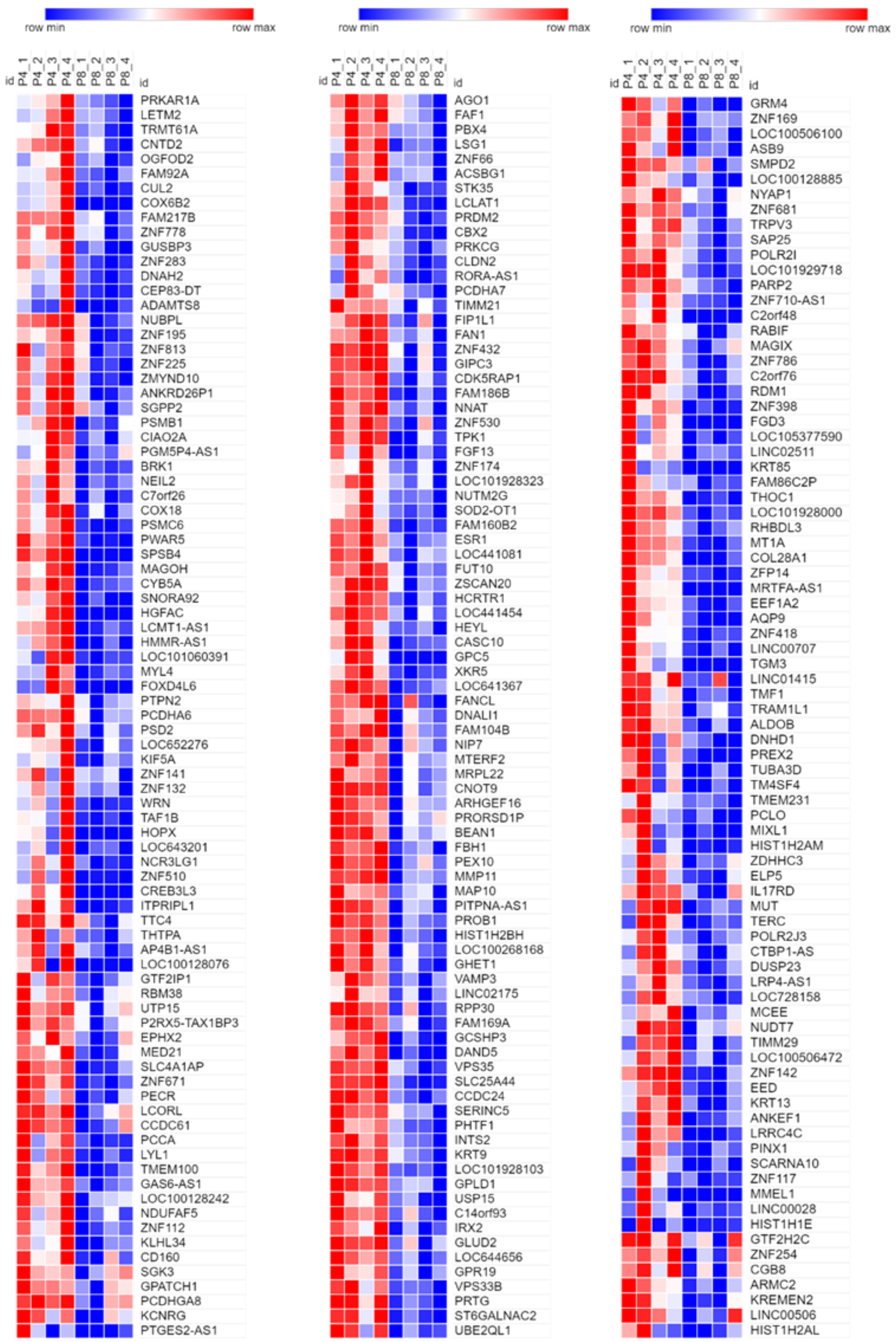

row min
row max
id P4_1 P4_2 P4_3 P4_4 P8_1 P8_2 P8_3 P8_4 id
PRKAR1A
LETM2
TRMT61A
CNTD2
OGFOD2
FAM92A
CUL2
COX6B2
FAM217B
ZNF778
GUSBP3
ZNF283
DNAH2
CEP83-DT
ADAMTS8
NUBPL
ZNF195
ZNF813
ZNF225
ZMYND10
ANKRD26P1
SGPP2
PSMB1
CIAO2A
PGM5P4-AS1
BRK1
NEIL2
C7orf26
COX18
PSMC6
PWAR5
SPSB4
MAGOH
CYB5A
SNORA92
HGFAC
LCMT1-AS1
HMMR-AS1
LOC101060391
MYL4
FOXD4L6
PTPN2
PCDHA6
PSD2
LOC652276
KIF5A
ZNF141
ZNF132
WRN
TAF1B
HOPX
LOC643201
NCR3LG1
ZNF510
CREB3L3
ITPRIPL1
TTC4
THTPA
AP4B1-AS1
LOC100128076
GTF2IP1
RBM38
UTP15
P2RX5-TAX1BP3
EPHX2
MED21
SLC4A1AP
ZNF671
PECR
LCORL
CCDC61
PCCA
LYL1
TMEM100
GAS6-AS1
LOC100128242
NDUFAF5
ZNF112
KLHL34
CD160
SGK3
GPATCH1
PCDHGA8
KCNRG
PTGES2-AS1
row min
row max
id P4_1 P4_2 P4_3 P4_4 P8_1 P8_2 P8_3 P8_4 id
AGO1
FAF1
PBX4
LSG1
ZNF66
ACSBG1
STK35
LCLAT1
PRDM2
CBX2
PRKCG
CLDN2
RORA-AS1
PCDHA7
TIMM21
FIP1L1
FAN1
ZNF432
GIPC3
CDK5RAP1
FAM186B
NNAT
ZNF530
TPK1
FGF13
ZNF174
LOC101928323
NUTM2G
SOD2-OT1
FAM160B2
ESR1
LOC441081
FUT10
ZSCAN20
HCRTR1
LOC441454
HEYL
CASC10
GPC5
XKR5
LOC641367
FANCL
DNALI1
FAM104B
NIP7
MTERF2
MRPL22
CNOT9
ARHGEF16
PRORSD1P
BEAN1
FBH1
PEX10
MMP11
MAP10
PITPNA-AS1
PROB1
HIST1H2BH
LOC100268168
GHET1
VAMP3
LINC02175
RPP30
FAM169A
GCSHP3
DAND5
VPS35
SLC25A44
CCDC24
SERINC5
PHTF1
INTS2
KRT9
LOC101928103
GPLD1
USP15
C14orf93
IRX2
GLUD2
LOC644656
GPR19
VPS33B
PRTG
ST6GALNAC2
UBE2QL1
row min
row max
id P4_1 P4_2 P4_3 P4_4 P8_1 P8_2 P8_3 P8_4 id
GRM4
ZNF169
LOC100506100
ASB9
SMPD2
LOC100128885
NYAP1
ZNF681
TRPV3
SAP25
POLR2I
LOC101929718
PARP2
ZNF710-AS1
C2orf48
RABIF
MAGIX
ZNF786
C2orf76
RDM1
ZNF398
FGD3
LOC105377590
LINC02511
KRT85
FAM86C2P
THOC1
LOC101928000
RHBDL3
MT1A
COL28A1
ZFP14
MRTFA-AS1
EEF1A2
AQP9
ZNF418
LINC00707
TGM3
LINC01415
TMF1
TRAM1L1
ALDOB
DNHD1
PREX2
TUBA3D
TM4SF4
TMEM231
PCLO
MIXL1
HIST1H2AM
ZDHHC3
ELP5
IL17RD
MUT
TERC
POLR2J3
CTBP1-AS
DUSP23
LRP4-AS1
LOC728158
MCEE
NUDT7
TIMM29
LOC100506472
ZNF142
EED
KRT13
ANKEF1
LRRC4C
PINX1
SCARNA10
ZNF117
MMEL1
LINC00028
HIST1H1E
GTF2H2C
ZNF254
CGB8
ARMC2
KREMEN2
LINC00506
HIST1H2AL

Supplementary Figure S14. Heatmaps for DEGs of P4 vs. P12 only in Venn Diagram.

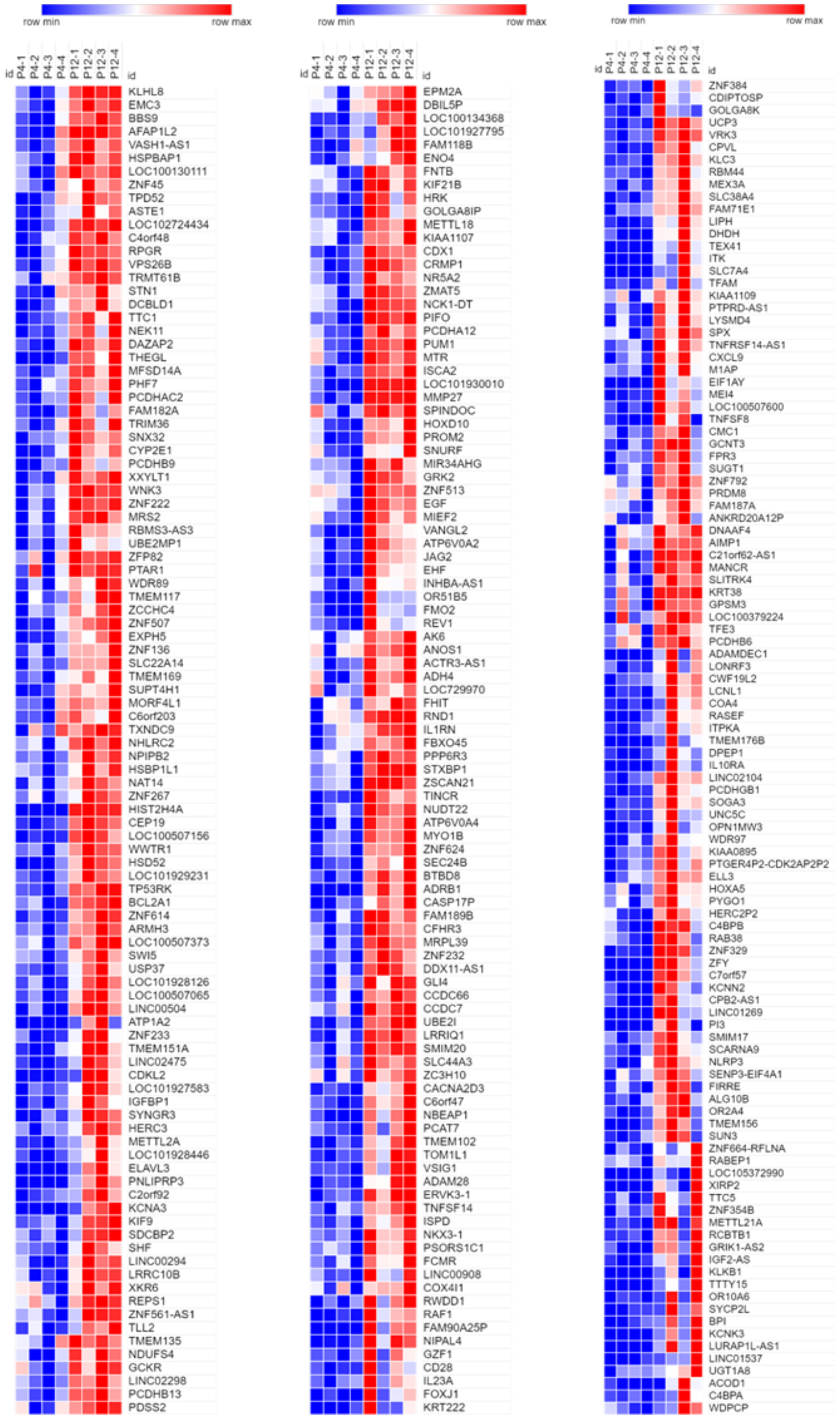

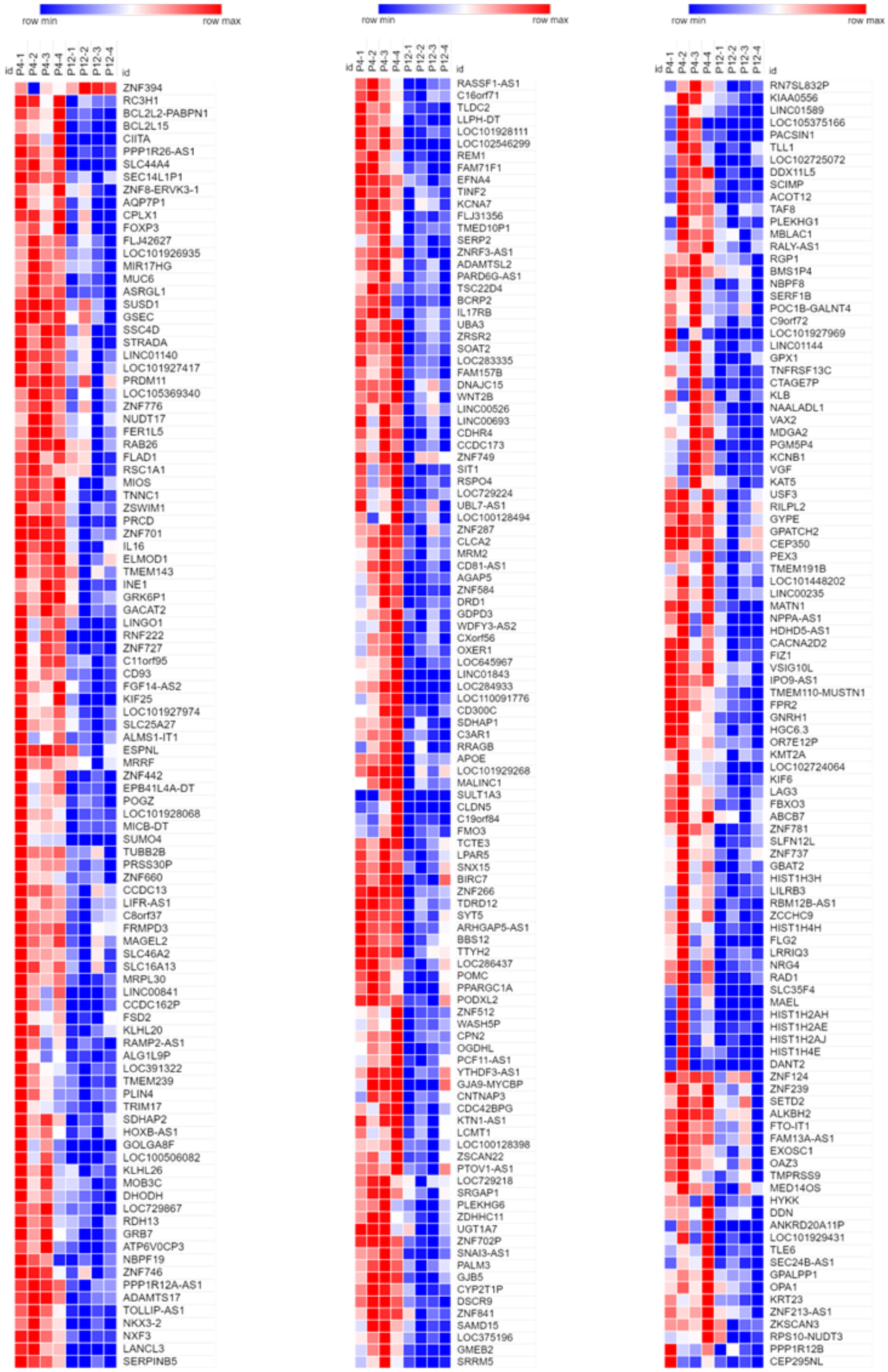

row min
row max
id
P4-1
P4-2
P4-3
P4-4
P12-1
P12-2
P12-3
P12-4
ZNF394
RC3H1
BCL2L2-PABPN1
BCL2L15
CIITA
PPP1R26-AS1
SLC44A4
SEC14L1P1
ZNF8-ERVK3-1
AQP7P1
CPLX1
FOXP3
FLJ42627
LOC101926935
MIR17HG
MUC6
ASRGL1
SUSD1
GSEC
SSC4D
STRADA
LINC01140
LOC101927417
PRDM11
LOC105369340
ZNF776
NUDT17
FER1L5
RAB26
FLAD1
RSC1A1
MIOS
TNNC1
ZSWIM1
PRCD
ZNF701
IL16
ELMOD1
TMEM143
INE1
GRK6P1
GACAT2
LINGO1
RNF222
ZNF727
C11orf95
CD93
FGF14-AS2
KIF25
LOC101927974
SLC25A27
ALMS1-IT1
ESPNL
MRRF
ZNF442
EPB41L4A-DT
POGZ
LOC101928068
MICB-DT
SUMO4
TUBB2B
PRSS30P
ZNF660
CCDC13
LIFR-AS1
C8orf37
FRMPD3
MAGEL2
SLC46A2
SLC16A13
MRPL30
LINC00841
CCDC162P
FSD2
KLHL20
RAMP2-AS1
ALG1L9P
LOC391322
TMEM239
PLIN4
TRIM17
SDHAP2
HOXB-AS1
GOLGA8F
LOC100506082
KLHL26
MOB3C
DHODH
LOC729867
RDH13
GRB7
ATP6V0CP3
NBPF19
ZNF746
PPP1R12A-AS1
ADAMTS17
TOLLIP-AS1
NKX3-2
NXF3
LANCL3
SERPINB5
row min
row max
id
P4-1
P4-2
P4-3
P4-4
P12-1
P12-2
P12-3
P12-4
RASSF1-AS1
C16orf71
TLDC2
LLPH-DT
LOC101928111
LOC102546299
REM1
FAM71F1
EFNA4
TINF2
KCNA7
FLJ31356
TMED10P1
SERP2
ZNRF3-AS1
ADAMTSL2
PARD6G-AS1
TSC22D4
BCRP2
IL17RB
UBA3
ZRSR2
SOAT2
LOC283335
FAM157B
DNAJC15
WNT2B
LINC00526
LINC00693
CDHR4
CCDC173
ZNF749
SIT1
RSPO4
LOC729224
UBL7-AS1
LOC100128494
ZNF287
CLCA2
MRM2
CD81-AS1
AGAP5
ZNF584
DRD1
GDPD3
WDFY3-AS2
CXorf56
OXER1
LOC645967
LINC01843
LOC284933
LOC110091776
CD300C
SDHAP1
C3AR1
RRAGB
APOE
LOC101929268
MALINC1
SULT1A3
CLDN5
C19orf84
FMO3
TCTE3
LPAR5
SNX15
BIRC7
ZNF266
TDRD12
SYT5
ARHGAP5-AS1
BBS12
TTYH2
LOC286437
POMC
PPARGC1A
PODXL2
ZNF512
WASH5P
CPN2
OGDHL
PCF11-AS1
YTHDF3-AS1
GJA9-MYCBP
CNTNAP3
CDC42BPG
KTN1-AS1
LCMT1
LOC100128398
ZSCAN22
PTOV1-AS1
LOC729218
SRGAP1
PLEKHG6
ZDHHC11
UGT1A7
ZNF702P
SNAI3-AS1
PALM3
GJB5
CYP2T1P
DSCR9
ZNF841
SAMD15
LOC375196
GMEB2
SRRM5
row min
row max
id
P4-1
P4-2
P4-3
P4-4
P12-1
P12-2
P12-3
P12-4
RN7SL832P
KIAA0556
LINC01589
LOC105375166
PACSIN1
TLL1
LOC102725072
DDX11L5
SCIMP
ACOT12
TAF8
PLEKHG1
MBLAC1
RALY-AS1
RGP1
BMS1P4
NBPF8
SERF1B
POC1B-GALNT4
C9orf72
LOC101927969
LINC01144
GPX1
TNFRSF13C
CTAGE7P
KLB
NAALADL1
VAX2
MDGA2
PGM5P4
KCNB1
VGF
KAT5
USF3
RILPL2
GYPE
GPATCH2
CEP350
PEX3
TMEM191B
LOC101448202
LINC00235
MATN1
NPPA-AS1
HDHD5-AS1
CACNA2D2
FIZ1
VSIG10L
IPO9-AS1
TMEM110-MUSTN1
FPR2
GNRH1
HGC6.3
OR7E12P
KMT2A
LOC102724064
KIF6
LAG3
FBXO3
ABCB7
ZNF781
SLFN12L
ZNF737
GBAT2
HIST1H3H
LILRB3
RBM12B-AS1
ZCCHC9
HIST1H4H
FLG2
LRRIQ3
NRG4
RAD1
SLC35F4
MAEL
HIST1H2AH
HIST1H2AE
HIST1H2AJ
HIST1H4E
DANT2
ZNF124
ZNF239
SETD2
ALKBH2
FTO-IT1
FAM13A-AS1
EXOSC1
OAZ3
TMPRSS9
MED14OS
HYKK
DDN
ANKRD20A11P
LOC101929431
TLE6
SEC24B-AS1
GPALPP1
OPA1
KRT23
ZNF213-AS1
ZKSCAN3
RPS10-NUDT3
PPP1R12B
CEP295NL

Supplementary Figure S15. Schematic of proteomics analysis work flow for hASCs at P4, P8, and P12.

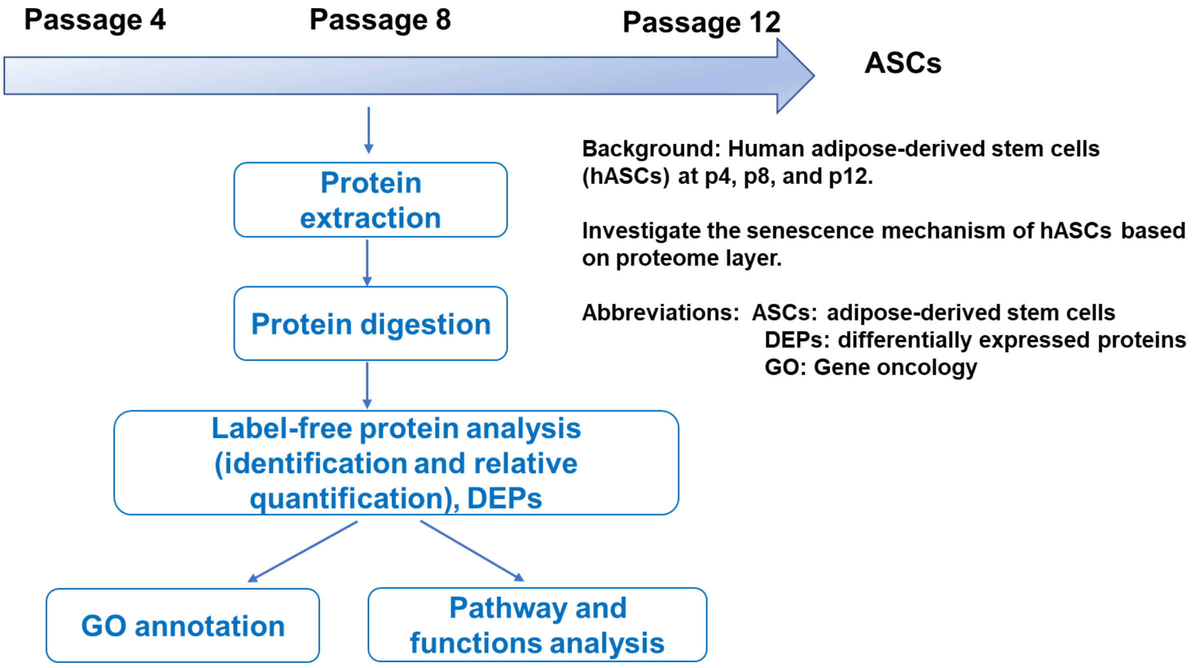

Supplementary Figure S16. GO analysis of DEPs (P8/P4 hASCs with 90 DEPs)

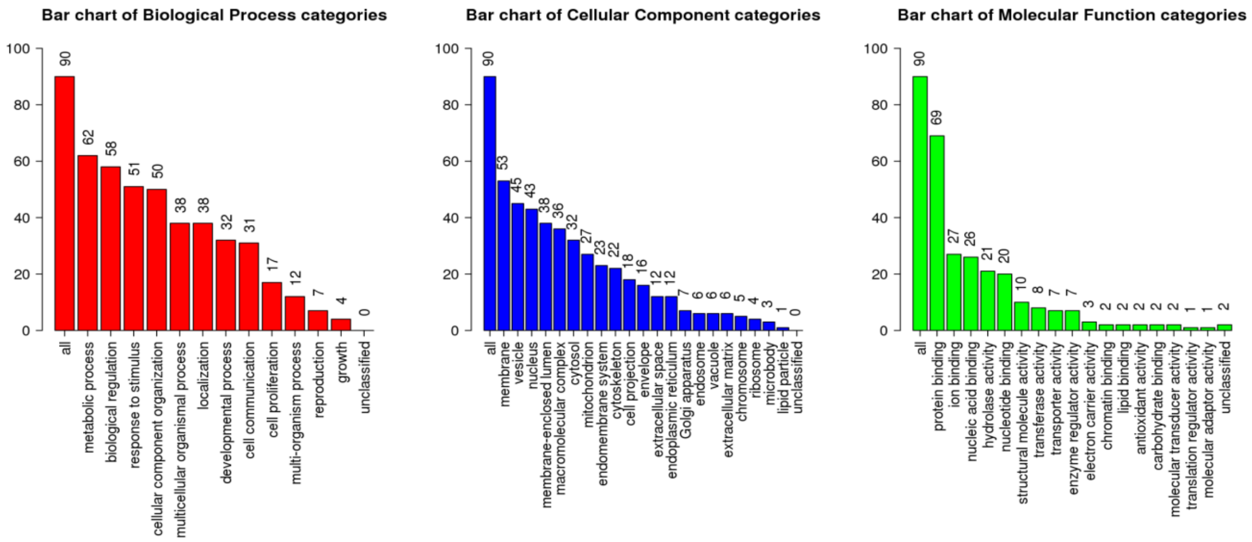

Supplementary Figure S17. Pathway enrichment analysis of DEPs (P8/P4)

A.

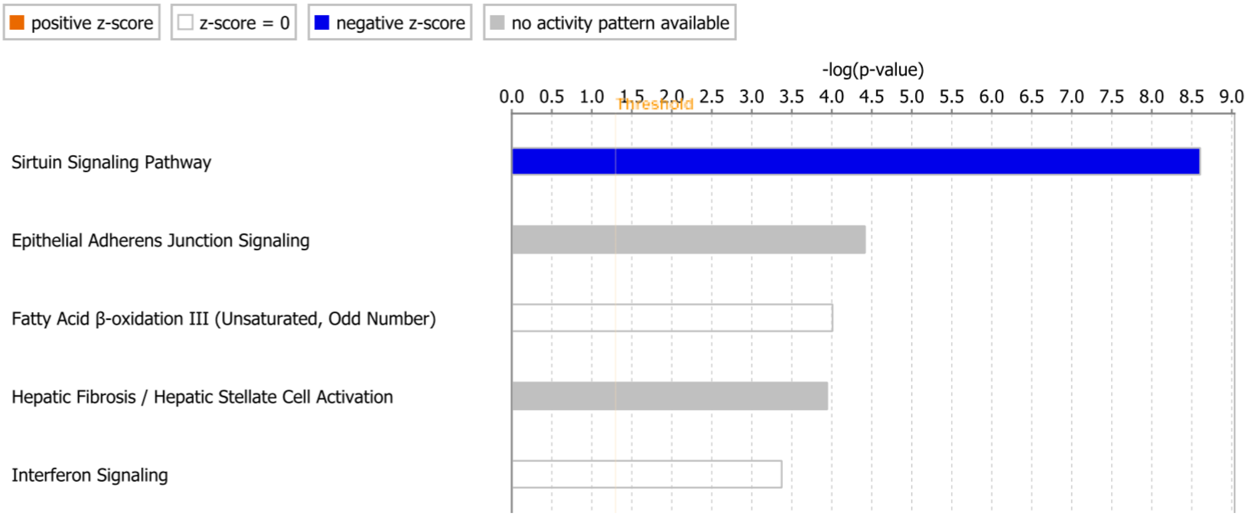

B.

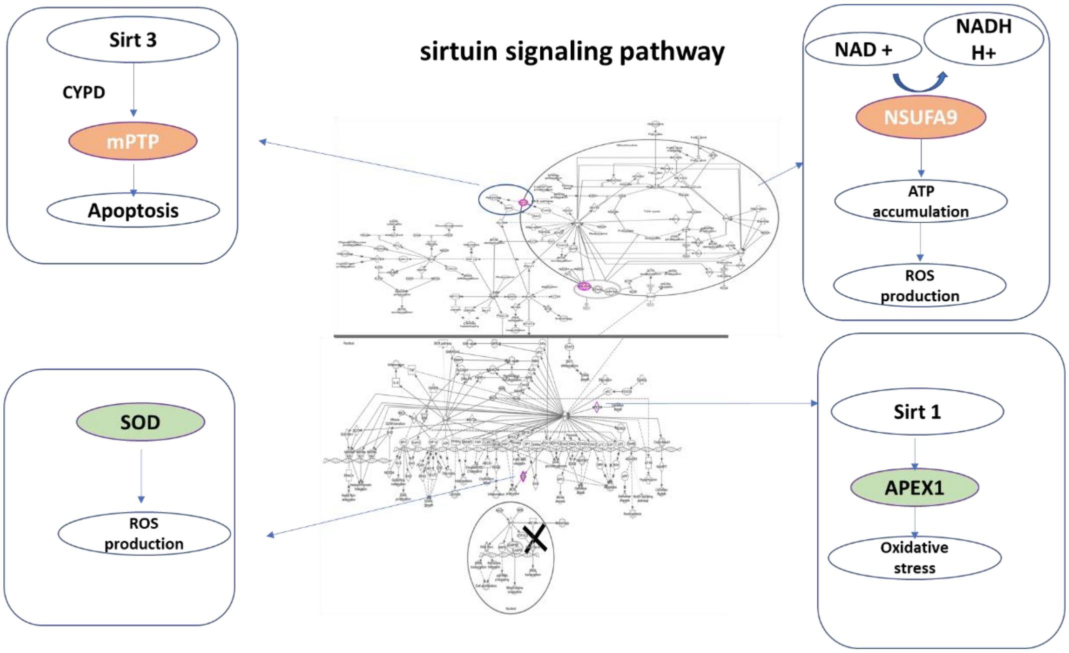

C.

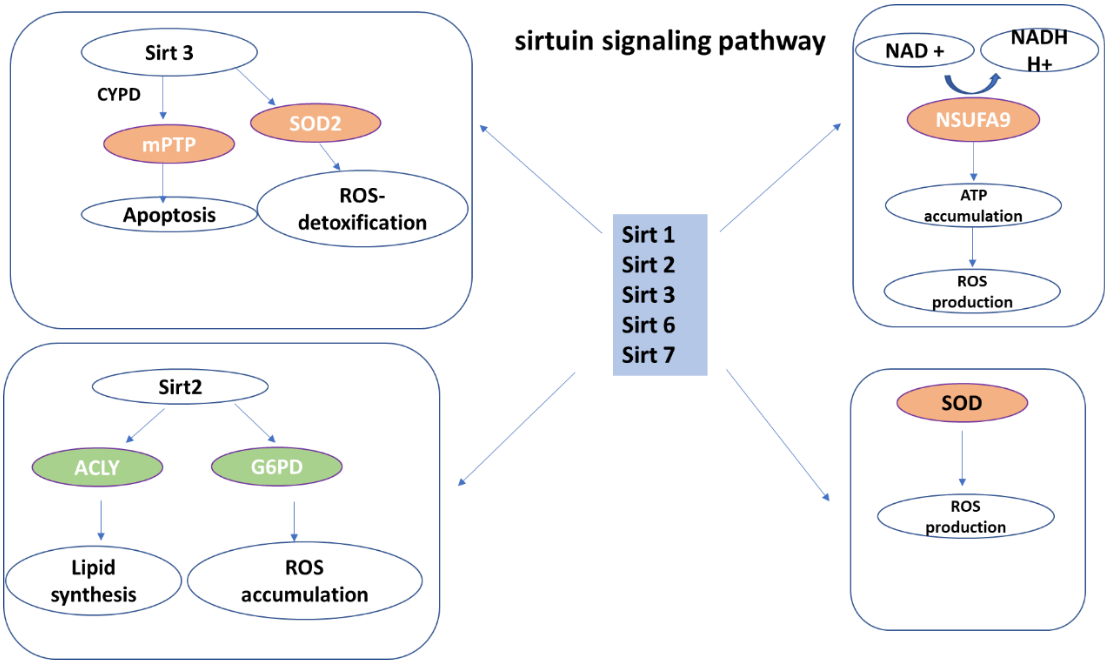

Supplementary Figure S18. GO analysis of DEPs overlapped between P8/P4 and P12/P4 hASCs.

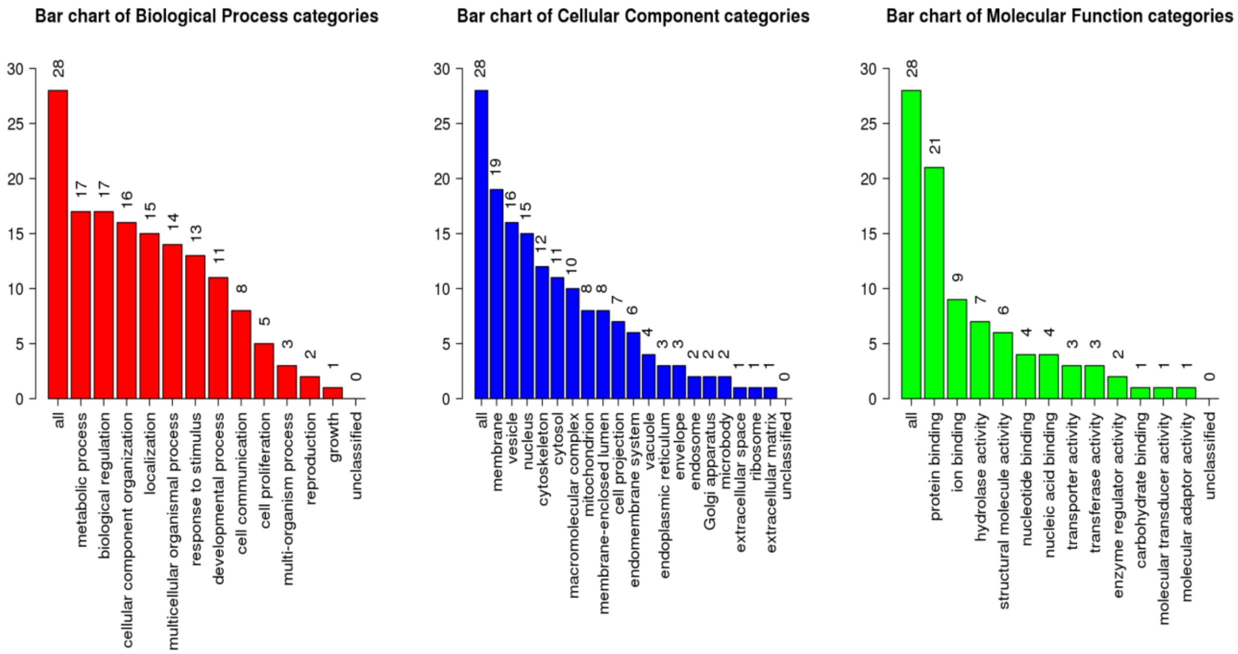

Supplementary materials for RNA-Seq data analysis

Supplementary Spreadsheet 1. "P4_vs_P12_Deseq2_Plus" for RNA-Seq data analysis.

DEGs for P4 and P12 comparison.

Supplementary Spreadsheet 2. "P4_vs_P8_Deseq2_Plus" for RNA-Seq data analysis.

DEGs for P4 and P8 comparison.

Supplementary Tables

Supplementary Table S1. The donor information for the derived hASCs used in this study.

ID	Years old	BMI	race
Donor1	43	24.8	caucasian
Donor2	40	21.1	caucasian
Donor3	39	<25	caucasian

Supplementary Table S2. Primer sequences for target genes in RT-PCR analysis.

Gene	Forward primer 5' to 3'	Reverse primer 5' to 3'
**Oct-4**	CAGCAGATCAGCCACATCGCC	TGAGAAAGGAGACCCAGCAGCC
**Nanog**	CCTGTGATTTGTGGGCCTG	GACAGTCTCCGTGTGAGGCAT
**SOX2**	GTATCAGGAGTTGTCAAGGCAGAG	TCCTAGTCTTAAAGAGGCAGCAAAC
**P53**	CTGGACGACAGGCAGACTTT	GCACAAACACGAACCTCAAA
**P15 (CDKN2B)**	GCTGTTTCATCAGCAGCCTAA	TTCCACAATGGAGCTAGAAGCA
**P21**	GAGACTCTCAGGGTCGAAAACG	GGATTAGGGCTTCCTCTTGGAG
**NFKB**	GACGAGCTCCGAGACAGTGAC	GAGGCACCACTGGTCAGAGAC
**mTOR**	GCCTGGATGGCAACTACAGAA	CCAGTTCAGCAAGGGGTCATA
**PIK3CA**	TTGGAGAACTTGGCCTTCATCT	ACCCAATTAGGTCTGAGGACTGAA
**AKT1**	TATCGTGTGGCAGCACGTGTA	GCCGTGAACTCCTCATCAAAAT
**PTEN**	GGCTGCTGTTGGCTTATCAGT	AAACGCGGTAATTTTCAGAGC
**SIRT1**	TCGCAACTATACCCAGAACATAGACA	CTGTTGCAAAGGAACCATGACA
**SIRT3**	GCATCCCTGCCTCAAAGC	CGTCAGCCCGAATGTCCTC
**CD38**	TTCCCGCAGTTTTTCTTTGAA	GCACCTCTCAGCTGCCTACTG
**CD73**	GCCTGGGAGCTTACGATTTTG	TAGTGCCCTGGTACTGGTCG
**NAMPT**	CTCCATTCTGAGCACCAAACG	ATGCCATTTGCTTTTGTCTGG
**PDK1**	AAACAGGGGAGCTTTGTCTGG	CTGCCCATTCACATCCCTCTA
**PKM2**	AAAAATGGATGCCCAGAGGAC	GAGTCGGCTTCAATGGAACAA
**LDHA**	CCTTGAGCCAGGTGGATGTTT	CACTGGATCCCAGGATGTGAC
**G6PD (GL6PD)**	CTACCCGAGCCCAGCTACATT	TTCTGTTGGGCTGGAGTGAGT

**6PGD (6PGLD)**	CCATGCCCTGTTTTACCACTG	AGGTGTGAGCCCCGAAGTAAT
**TALDO1**	CTGTCATCAACCTGGGAAGGA	GGGCGAAGGAGAAGAGTAACG
**TKTL1**	ACCTTGGGATTCTGTGTGCTG	CCTAACAAGCTTTCGCTGCTG
**TNFα**	TGGCCAATGGCGTGGAGCTG	GTAGGAGACGGCGATGCGGC
**IL-6**	GAACTCCTTCTCCACAAGCG	TTTTCTGCCAGTGCCTCTTT
**IL-1β**	CCACAGACCTTCCAGGAGAATG	GTGCAGTTCAGTGATCGTACAGG
**IL-12β**	CCAAGGGGTGACGTGCGGAG	GGTGGGTCAGGTTTGATGATGTCCC
**IL-10**	AAGCCTGACCACGCTTTCTA	ATGAAGTGGTTGGGGAATGA
**TGF- β**	CCTACATTTGGAGCCTGGAC	TGTCCTTAAATACAGCCCCC
**CD163**	*CCAGTCCCAAACACTGTCCT*	*ATGCCAGTGAGCTTCCCGTTCAGC*
**ALP**	CAGTCTGCTGTGCCCCTGC	GTAGTTCTGCTCGTGGACGCC
**BMP2**	TTCCCCGTGACCAGACTTTTGG	GCCACTTCCACCACGAATCCAT
**Osteo-calcin**	GGCAGCGAGGTAGTGAAGAGAC	GAAAGCCGATGTGGTCAGCCAA
**Osteo-pontin**	AGCGGAAAGCCAATGATGAGAGC	ACTTTTGGGGTCTACAACCAGCAT
**Runx2**	CCAACCCACGAATGCACTATC	TAGTGAGTGGTGGCGGACATAC
**CEBPA**	AGCCTTGTTTGTACTGTATG	AAAATGGTGGTTTAGCAGAG
**FABP4**	ACGAGAGGATGATAAACTGGTGG	GCGAACTTCAGTCCAGGTCAAC
**LPL**	CTGACCAAGGATAGTGGGATATAG	GGTAACTGAGCGAGACTGTGTCT
**PPARG**	AGCCTGCGAAAGCCTTTTGGTG	GGCTTCACATTCAGCAAACCTGG
**TFEB**	CCTGGTGGAGATTCCCTGTCT	CAGGACCAGTTGCCTCAGATG
**BECN1**	ACTGTGTTGCTGCTCCATGCT	AACGGCAGCTCCTTAGATTTGT
**LAMP1**	TCACACGTAGGACGCATGAAG	GAAGCGCTCCAGACACTCATC
**MFN1**	AAATGCTCAAAGGGTGCTCCT	GATGCATTATCTGGCGTTGCT
**MFN2**	GATGCCTGTCACCAAGGTGTT	TGCTTTTTGGGAGAGGTGTTG
**FIS1**	CTGGTGCGGAGCAAGTACAAT	CACGGCCAGGTAGAAGACGTA
**DNM1L**	CGATGCACTTTTCTCCAGCAC	TTCCGTTGTTTCTTGCCTCTG
**GAPDH**	TCACTGCCACCCAGAAGACTG	GGATGACCTTGCCCACAGC
**TUB1a (TUBA1A)**	TGAGGAGGTTGGTGTGGATTC	AAAAGCAGCACCTTTGTGACG

Supplementary Table S3. DEPs between P8 and P4 hASCs. For a total of 90 DEPs, 44 were up regulated and 46 were down regulated.

Protein name	Gene	Molecular Weight	Fold Change (p8 vs p4)
Protein AHNAK2	AHNAK2	617 kDa	8
Keratin, type I cytoskeletal 14	KRT14	52 kDa	6.6
Enoyl-CoA delta isomerase 2, mitochondrial	ECI2	44 kDa	5.2
Desmin	DES	54 kDa	5.2
Delta(3,5)-Delta(2,4)-dienoyl-CoA isomerase, mitochondrial	ECH1	36 kDa	3.8
Fibrillin-1	FBN1	312 kDa	3.3
Keratin, type II cytoskeletal 8	KRT8	54 kDa	3.3
Collagen alpha-3(VI) chain	COL6A3	344 kDa	3.3
Paraspeckle component 1	PSPC1	59 kDa	3.2
Nuclear mitotic apparatus protein 1	NUMA1	238 kDa	3.1
NADH dehydrogenase [ubiquinone] 1 alpha subcomplex subunit 12	NDUFA12	17 kDa	3
Probable 28S rRNA (cytosine(4447)-C(5))-methyltransferase	NOP2	89 kDa	2.9
Acyl-coenzyme A thioesterase 9, mitochondrial	ACOT9	50 kDa	2.8
Lysosomal protective protein	CTSA	54 kDa	2.7
NAD-dependent malic enzyme, mitochondrial	ME2	65 kDa	2.7
Ribosome production factor 2 homolog	RPF2	36 kDa	2.7
Early endosome antigen 1	EEA1	162 kDa	2.7
Pyrroline-5-carboxylate reductase 1, mitochondrial	PYCR1	33 kDa	2.7
Ferrochelatase, mitochondrial	FECH	48 kDa	2.6
Lon protease homolog, mitochondrial	LONP1	106 kDa	2.6
Serine/arginine repetitive matrix protein 2	SRRM2	300 kDa	2.5
Mitochondrial import inner membrane translocase subunit Tim13	TIMM13	11 kDa	2.5
Monofunctional C1-tetrahydrofolate synthase, mitochondrial	MTHFD1L	106 kDa	2.5
Heat shock protein beta-7	HSPB7	19 kDa	2.5
Ras-related protein R-Ras2	RRAS2	23 kDa	2.4
Mitochondrial import receptor subunit TOM40 homolog	TOMM40	38 kDa	2.4
Semaphorin-7A	SEMA7A	75 kDa	2.4
Transmembrane emp24 domain-containing protein 5	TMED5	26 kDa	2.4
U1 small nuclear ribonucleoprotein C	SNRPC	17 kDa	2.4
NADH dehydrogenase [ubiquinone] flavoprotein 1, mitochondrial	NDUFV1	51 kDa	2.3

**Adenosine deaminase**	**ADA**	**41 kDa**	**2.3**
**Heterogeneous nuclear ribonucleoprotein F**	**HNRNPF**	**46 kDa**	**2.2**
**NADH dehydrogenase [ubiquinone] 1 alpha subcomplex subunit 9, mitochondrial**	**NDUFA9**	**43 kDa**	**2.2**
**40S ribosomal protein S27-like**	**RPS27L**	**9 kDa**	**2.2**
**Collagen alpha-2(VI) chain**	**COL6A2**	**109 kDa**	**2.2**
**Flotillin-1**	**FLOT1**	**47 kDa**	**2.2**
**Voltage-dependent anion-selective channel protein 3**	**VDAC3**	**31 kDa**	**2.2**
**Polycystin-2**	**PKD2**	**110 kDa**	**2.1**
**Epidermal growth factor receptor**	**EGFR**	**134 kDa**	**2.1**
**Isochorismatase domain-containing protein 2**	**ISOC2**	**22 kDa**	**2.1**
**ADP/ATP translocase 2**	**SLC25A5**	**33 kDa**	**2.1**
**Mitochondrial import inner membrane translocase subunit TIM50**	**TIMM50**	**40 kDa**	**2**
**Enoyl-CoA delta isomerase 1, mitochondrial**	**ECI1**	**33 kDa**	**2**
**NADH dehydrogenase [ubiquinone] 1 alpha subcomplex subunit 8**	**NDUFA8**	**20 kDa**	**2**
**Acid ceramidase**	**ASAH1**	**45 kDa**	**-2**
**Protein S100-A4**	**S100A4**	**12 kDa**	**-2**
**60S ribosomal protein L26**	**RPL26**	**17 kDa**	**-2**
**Exportin-1**	**XPO1**	**123 kDa**	**-2**
**Glutamine--fructose-6-phosphate aminotransferase [isomerizing] 1**	**GFPT1**	**79 kDa**	**-2**
**Isocitrate dehydrogenase [NADP] cytoplasmic**	**IDH1**	**47 kDa**	**-2**
**Prostaglandin G/H synthase 1**	**PTGS1**	**69 kDa**	**-2**
**Bcl-2-associated transcription factor 1**	**BCLAF1**	**106 kDa**	**-2**
**DNA-(apurinic or apyrimidinic site) lyase**	**APEX1**	**36 kDa**	**-2**
**Peptidyl-tRNA hydrolase 2, mitochondrial**	**PTRH2**	**19 kDa**	**-2**
**Interferon-induced protein with tetratricopeptide repeats 3**	**IFIT3**	**56 kDa**	**-2**
**ATP-dependent DNA helicase Q1**	**RECQL**	**73 kDa**	**-2**
**60 kDa SS-A/Ro ribonucleoprotein**	**TROVE2**	**61 kDa**	**-2**
**Peptidase inhibitor 16**	**PI16**	**49 kDa**	**-2.5**
**Aldose reductase**	**AKR1B1**	**36 kDa**	**-2.5**
**High mobility group protein B1**	**HMGB1**	**25 kDa**	**-2.5**
**Microtubule-associated protein RP/EB family member 1**	**MAPRE1**	**30 kDa**	**-2.5**

ATP-dependent 6-phosphofructokinase, platelet type	PFKP	86 kDa	-2.5
EH domain-containing protein 1	EHD1	61 kDa	-2.5
Malignant T-cell-amplified sequence 1	MCTS1	21 kDa	-2.5
Ubiquitin carboxyl-terminal hydrolase 5	USP5	96 kDa	-2.5
Cysteine and glycine-rich protein 2	CSRP2	21 kDa	-2.5
Drebrin-like protein	DBNL	48 kDa	-2.5
Actin-related protein 2/3 complex subunit 3	ARPC3	21 kDa	-2.5
ADP/ATP translocase 1	SLC25A4	33 kDa	-2.5
Bifunctional purine biosynthesis protein PURH	ATIC	65 kDa	-2.5
Hepatoma-derived growth factor	HDGF	27 kDa	-2.5
Sorbin and SH3 domain-containing protein 2	SORBS2	124 kDa	-2.5
Interferon-induced GTP-binding protein Mx2	MX2	82 kDa	-2.5
Puromycin-sensitive aminopeptidase	NPEPPS	103 kDa	-2.5
Protein YIF1B	YIF1B	34 kDa	-2.5
Interferon-induced protein with tetratricopeptide repeats 1	IFIT1	55 kDa	-2.5
A-kinase anchor protein 12	AKAP12	191 kDa	-2.5
1,4-alpha-glucan-branching enzyme	GBE1	80 kDa	-2.5
NAD(P)H dehydrogenase [quinone] 1	NQO1	31 kDa	-2.5
Calponin-1	CNN1	33 kDa	-3.3
Myosin-10	MYH10	229 kDa	-3.3
Phosphoglucomutase-1	PGM1	61 kDa	-3.3
Purine nucleoside phosphorylase	PNP	32 kDa	-3.3
Integrin alpha-1	ITGA1	131 kDa	-3.3
Kinesin-1 heavy chain	KIF5B	110 kDa	-3.3
Dihydropyrimidinase-related protein 3	DPYSL3	62 kDa	-3.3
Myosin light chain 1/3, skeletal muscle isoform	MYL1	21 kDa	-5
Interferon-induced GTP-binding protein Mx1	MX1	76 kDa	-5
Protein-glutamine gamma-glutamyltransferase 2	TGM2	77 kDa	-10
Myosin-7	MYH7	223 kDa	-14.3

Supplementary Table S4. Upstream analysis of DEPs (P8/P4 hASCs)

Upstream Regulator	Molecule Type	Predicted Activation State	Activation z-score	p-value of overlap	Target molecules in dataset
MAPK1	kinase	Activated	2.236	0.0157	EHD1,IFIT1,IFIT3,MX2,MYH7
SB203580	chemical - kinase inhibitor	Activated	2.213	0.0413	IFIT1,IFIT3,MX1,NQO1,TGM2
fluticasone propionate	chemical drug	Activated	2	0.00578	IFIT1,MX1,PTGS1,TGM2
mir-122	microRNA	Activated	2	0.000531	KIF5B,MAPRE1,NPEPPS,TGM2
BTK	kinase	Activated	2	0.00012	CTSA,IFIT1,IFIT3,MX1,MX2
oblimersen	biologic drug	Inhibited	-2	7.91E-05	IFIT1,IFIT3,MX1,MX2
TLR7	transmembrane receptor	Inhibited	-2	0.00366	IFIT1,IFIT3,MX1,MX2
CREB1	transcription regulator	Inhibited	-2	0.00916	ARPC3,CNN1,CSRP2,MYH10,NDUFV1,RRAS2,SEMA7A
lipopolysaccharide	chemical drug	Inhibited	-2.033	0.00129	ADA,APEX1,EHD1,FBN1,HDGF,HMGB1,IDH1,IFIT1,IFIT3,MCTS1,MX1,MX2,MYH7,NQO1,PFKP,PTGS1,TGM2
Interferon alpha	group	Inhibited	-2.082	0.0378	EGFR,IFIT1,IFIT3,MX1,MX2
IFNA2	cytokine	Inhibited	-2.18	0.00314	IFIT1,IFIT3,MX1,MX2,TGM2
IL1B	cytokine	Inhibited	-2.551	0.00174	AKR1B1,HMGB1,IFIT1,IFIT3,ITGA1,MX1,NQO1,PFKP,PTGS1,SLC25A4,TGM2
tretinoin	chemical - endogenous mammalian	Inhibited	-2.636	1.73E-06	ADA,AKAP12,APEX1,COL6A3,DPYSL3,ECI2,EGFR,EHD1,HDGF,HNRNPF,IFIT1,IFIT3,ITGA1,KRT14,MYH7,NDUFV1,PNP,PTGS1,RPF2,SEMA7A,TGM2

Supplementary Table S5. DEPs between P12 and P4 hASCs. For a total of 318 DEPs, 86 were up regulated, and 232 were down regulated.

Protein name	Gene name	Molecular Weight	Fold Change by p12 vs p4
Dipeptidyl peptidase 4	DPP4	88 kDa	23
Integrin alpha-2	ITGA2	129 kDa	23
Calponin-1	CNN1	33 kDa	22
Keratin, type I cytoskeletal 14	KRT14	52 kDa	18
Lactadherin	MFGE8	43 kDa	11
Voltage-dependent calcium channel subunit alpha-2/delta-1	CACNA2D 1	125 kDa	9
Delta-sarcoglycan	SGCD	32 kDa	8.3
Synaptopodin-2	SYNPO2	118 kDa	6.6
Raftlin	RFTN1	63 kDa	6.3
Nexilin	NEXN	81 kDa	5.5
Flotillin-1	FLOT1	47 kDa	5.5
Plasma membrane calcium-transporting ATPase 4	ATP2B4	138 kDa	5.4
Ras-related protein R-Ras2	RRAS2	23 kDa	5.3
V-type proton ATPase 116 kDa subunit a isoform 1	ATP6V0A1	96 kDa	5.2
Sorbin and SH3 domain-containing protein 2	SORBS2	124 kDa	5.1
ADP-ribosyl cyclase/cyclic ADP-ribose hydrolase 2	BST1	36 kDa	4.8
Melanotransferrin	MELTF	80 kDa	4.7
Myosin regulatory light polypeptide 9	MYL9	20 kDa	4.5
Cysteine and glycine-rich protein 2	CSRP2	21 kDa	4.4
Myosin-10	MYH10	229 kDa	4.4
Myosin phosphatase Rho-interacting protein	MPRIP	117 kDa	4.4
Intercellular adhesion molecule 1	ICAM1	58 kDa	4.1
Actin, aortic smooth muscle	ACTA2	42 kDa	4.1
Superoxide dismutase [Mn], mitochondrial	SOD2	25 kDa	4
Solute carrier family 2, facilitated glucose transporter member 1	SLC2A1	54 kDa	4
Lysosomal protective protein	CTSA	54 kDa	3.8
Ankycorbin	RAI14	110 kDa	3.8
Large neutral amino acids transporter small subunit 1	SLC7A5	55 kDa	3.8
Aldehyde dehydrogenase X, mitochondrial	ALDH1B1	57 kDa	3.7
Matrix-remodeling-associated protein 7	MXRA7	21 kDa	3.7

Cell migration-inducing and hyaluronan-binding protein	CEMIP	153 kDa	3.7
Plasma membrane calcium-transporting ATPase 1	ATP2B1	135 kDa	3.4
Endoplasmic reticulum junction formation protein lunapark	LNPK	48 kDa	3.2
HLA class I histocompatibility antigen, B-44 alpha chain	HLA-B	40 kDa	3.2
BAG family molecular chaperone regulator 2	BAG2	24 kDa	3.1
ADP/ATP translocase 1	SLC25A4	33 kDa	3.1
Glutaredoxin-1	GLRX	12 kDa	3
Integrin beta-1	ITGB1	88 kDa	2.9
Selenoprotein M	SELENOM	16 kDa	2.9
Stromal interaction molecule 1	STIM1	77 kDa	2.9
Gamma-glutamyl hydrolase	GGH	36 kDa	2.9
Acid ceramidase	ASAH1	45 kDa	2.9
Mitochondrial import inner membrane translocase subunit Tim13	TIMM13	11 kDa	2.8
Peroxisomal acyl-coenzyme A oxidase 1	ACOX1	74 kDa	2.8
Guanine nucleotide-binding protein subunit beta-4	GNB4	38 kDa	2.7
Delta(3,5)-Delta(2,4)-dienoyl-CoA isomerase, mitochondrial	ECH1	36 kDa	2.7
Utrophin	UTRN	394 kDa	2.7
Retinol dehydrogenase 10	RDH10	38 kDa	2.7
Microtubule-associated protein 1A	MAP1A	305 kDa	2.6
DnaJ homolog subfamily C member 5	DNAJC5	22 kDa	2.6
Polycystin-2	PKD2	110 kDa	2.6
Multidrug resistance-associated protein 4	ABCC4	150 kDa	2.6
Perilipin-2	PLIN2	48 kDa	2.6
GRAM domain-containing protein 2B	GRAMD2B	48 kDa	2.6
Cysteine and glycine-rich protein 1	CSRP1	21 kDa	2.5
N-acetylglucosamine-6-sulfatase	GNS	62 kDa	2.5
Copine-3	CPNE3	60 kDa	2.5
Polypeptide N-acetylgalactosaminyltransferase 2	GALNT2	65 kDa	2.5
Annexin A7	ANXA7	53 kDa	2.5
Enoyl-CoA delta isomerase 2, mitochondrial	ECI2	44 kDa	2.5

Heterogeneous nuclear ribonucleoprotein H3	HNRNPH3	37 kDa	0.5
Septin-7	SEPT7	51 kDa	0.5
Heterogeneous nuclear ribonucleoprotein A1	HNRNPA1	39 kDa	0.5
Procollagen galactosyltransferase 1	COLGALT1	72 kDa	0.5
Eukaryotic translation initiation factor 4 gamma 1	EIF4G1	175 kDa	0.5
Peptidyl-prolyl cis-trans isomerase FKBP3	FKBP3	25 kDa	0.5
Collagen alpha-1(XII) chain	COL12A1	333 kDa	0.5
Peptidyl-prolyl cis-trans isomerase FKBP7	FKBP7	26 kDa	0.5
ADP-ribosylation factor-like protein 1	ARL1	20 kDa	0.5
40S ribosomal protein S28	RPS28	8 kDa	0.5
Elongation factor 2	EEF2	95 kDa	0.5
Nuclear transport factor 2	NUTF2	14 kDa	0.5
Heterogeneous nuclear ribonucleoprotein U	HNRNPU	91 kDa	0.5
E3 ubiquitin/ISG15 ligase TRIM25	TRIM25	71 kDa	0.5
Proteasome subunit beta type-1	PSMB1	26 kDa	0.5
Coiled-coil domain-containing protein 47	CCDC47	56 kDa	0.5
Vigilin	HDLBP	141 kDa	0.5
Far upstream element-binding protein 1	FUBP1	68 kDa	0.5
Splicing factor 3A subunit 3	SF3A3	59 kDa	0.5
26S proteasome non-ATPase regulatory subunit 4	PSMD4	41 kDa	0.5
ATP-dependent RNA helicase A	DHX9	141 kDa	0.5
RNA-binding protein Raly	RALY	32 kDa	0.5
Serine hydroxymethyltransferase, mitochondrial	SHMT2	56 kDa	0.5
Guanine nucleotide-binding protein subunit alpha-13	GNA13	44 kDa	0.5
Cytoplasmic dynein 1 heavy chain 1	DYNC1H1	532 kDa	0.5
60S ribosomal protein L36	RPL36	12 kDa	0.5
Elongation factor 1-alpha 1	EEF1A1	50 kDa	0.5
RNA-binding protein 3	RBM3	17 kDa	0.5
26S proteasome regulatory subunit 7	PSMC2	49 kDa	0.5

Leucyl-cystinyl aminopeptidase	LNPEP	117 kDa	2.4
Reticulon-3	RTN3	113 kDa	2.4
Neudesin	NENF	19 kDa	2.4
Multidrug resistance-associated protein 1	ABCC1	172 kDa	2.4
Solute carrier family 12 member 4	SLC12A4	121 kDa	2.3
Dynamin-like 120 kDa protein, mitochondrial	OPA1	112 kDa	2.3
Tropomyosin alpha-3 chain	TPM3	33 kDa	2.3
PDZ and LIM domain protein 5	PDLIM5	64 kDa	2.3
Plasminogen activator inhibitor 1	SERPINE1	45 kDa	2.2
Lysosome membrane protein 2	SCARB2	54 kDa	2.2
MICOS complex subunit MIC19	CHCHD3	26 kDa	2.2
Connective tissue growth factor	CTGF	38 kDa	2.2
V-type proton ATPase subunit d 1	ATP6V0D1	40 kDa	2.2
Procollagen-lysine,2-oxoglutarate 5-dioxygenase 2	PLOD2	85 kDa	2.2
Lysosomal acid glucosylceramidase	GBA	60 kDa	2.2
Protein O-mannosyl-transferase TMTC3	TMTC3	104 kDa	2.1
ATP synthase subunit gamma, mitochondrial	ATP5F1C	33 kDa	2.1
Guanine nucleotide-binding protein G(q) subunit alpha	GNAQ	42 kDa	2.1
Thy-1 membrane glycoprotein	THY1	18 kDa	2.1
Calpain-1 catalytic subunit	CAPN1	82 kDa	2.1
Glia-derived nexin	SERPINE2	44 kDa	2.1
Lysyl oxidase homolog 2	LOXL2	87 kDa	2.1
Inorganic pyrophosphatase	PPA1	33 kDa	2.1
CD166 antigen	ALCAM	65 kDa	2.1
Tropomyosin beta chain	TPM2	33 kDa	2
NADH dehydrogenase [ubiquinone] 1 alpha subcomplex subunit 9, mitochondrial	NDUFA9	43 kDa	2
Moesin	MSN	68 kDa	0.5
Nascent polypeptide-associated complex subunit alpha, muscle-specific form	NACA	205 kDa	0.5
Poly(U)-binding-splicing factor PUF60	PUF60	60 kDa	0.5
Interleukin enhancer-binding factor 3	ILF3	95 kDa	0.5
Nucleolar protein 58	NOP58	60 kDa	0.5
Oligoribonuclease, mitochondrial	REXO2	27 kDa	0.5
Caprin-1	CAPRIN1	78 kDa	0.5
Ribosomal L1 domain-containing protein 1	RSL1D1	55 kDa	0.5
RNA-binding protein FUS	FUS	53 kDa	0.5
Insulin-like growth factor 2 mRNA-binding protein 2	IGF2BP2	66 kDa	0.5
Keratin, type II cytoskeletal 8	KRT8	54 kDa	0.5
Neutral amino acid transporter B(0)	SLC1A5	57 kDa	0.5
Alpha-enolase	ENO1	47 kDa	0.5
Collagen alpha-1(I) chain	COL1A1	139 kDa	0.5
Sorting nexin-9	SNX9	67 kDa	0.5
26S proteasome regulatory subunit 6A	PSMC3	49 kDa	0.5
Plectin	PLEC	532 kDa	0.4
Spliceosome RNA helicase DDX39B	DDX39B	49 kDa	0.4
40S ribosomal protein S10	RPS10	19 kDa	0.4
Glucose-6-phosphate isomerase	GPI	63 kDa	0.4
Rab GDP dissociation inhibitor beta	GDI2	51 kDa	0.4
Dynein light chain 1, cytoplasmic	DYNLL1	10 kDa	0.4
Proteasome subunit alpha type-7	PSMA7	28 kDa	0.4
Glutamine--fructose-6-phosphate aminotransferase [isomerizing] 1	GFPT1	79 kDa	0.4
Non-POU domain-containing octamer-binding protein	NONO	54 kDa	0.4
Poly(rC)-binding protein 2	PCBP2	39 kDa	0.4
Creatine kinase B-type	CKB	43 kDa	0.4
Alpha-centractin	ACTR1A	43 kDa	0.4
Emerin	EMD	29 kDa	0.4
26S proteasome non-ATPase regulatory subunit 2	PSMD2	100 kDa	0.4
Heterogeneous nuclear ribonucleoprotein R	HNRNPR	71 kDa	0.4
Eukaryotic translation initiation factor 5A-1	EIF5A	17 kDa	0.4
Probable ATP-dependent RNA helicase DDX17	DDX17	80 kDa	0.4
Actin-related protein 2/3 complex subunit 3	ARPC3	21 kDa	0.4
Histone H2B type 1-B	HIST1H2BB	14 kDa	0.4

Serine/arginine-rich splicing factor 9	SRSF9	26 kDa	0.4
Histone H4	HIST1H4A	11 kDa	0.4
Myristoylated alanine-rich C-kinase substrate	MARCKS	32 kDa	0.4
Proteasome subunit beta type-4	PSMB4	29 kDa	0.4
Heterogeneous nuclear ribonucleoprotein L	HNRNPL	64 kDa	0.4
Drebrin-like protein	DBNL	48 kDa	0.4
Serine/threonine-protein phosphatase PP1-alpha catalytic subunit	PPP1CA	38 kDa	0.4
Transforming growth factor beta-1-induced transcript 1 protein	TGFB1I1	50 kDa	0.4
Splicing factor U2AF 65 kDa subunit	U2AF2	54 kDa	0.4
Microtubule-associated protein RP/EB family member 1	MAPRE1	30 kDa	0.4
U1 small nuclear ribonucleoprotein 70 kDa	SNRNP70	52 kDa	0.4
Integrin-linked protein kinase	ILK	51 kDa	0.4
Signal recognition particle subunit SRP72	SRP72	75 kDa	0.4
U1 small nuclear ribonucleoprotein A	SNRPA	31 kDa	0.4
26S proteasome non-ATPase regulatory subunit 14	PSMD14	35 kDa	0.4
Nesprin-1	SYNE1	1011 kDa	0.4
Protein NOXP20	FAM114A1	61 kDa	0.4
Eukaryotic translation initiation factor 3 subunit F	EIF3F	38 kDa	0.4
Alpha-parvin	PARVA	42 kDa	0.4
TAR DNA-binding protein 43	TARDBP	45 kDa	0.4
Nucleoside diphosphate kinase B	NME2	17 kDa	0.4
Phenylalanine--tRNA ligase alpha subunit	FARSA	58 kDa	0.4
Eukaryotic translation initiation factor 3 subunit D	EIF3D	64 kDa	0.4
Far upstream element-binding protein 3	FUBP3	62 kDa	0.4
Sorting nexin-3	SNX3	19 kDa	0.4
X-ray repair cross-complementing protein 6	XRCC6	70 kDa	0.4
Protein transport protein Sec24D	SEC24D	113 kDa	0.4
THO complex subunit 4	ALYREF	27 kDa	0.4
Pre-mRNA-processing-splicing factor 8	PRPF8	274 kDa	0.4
Nuclease-sensitive element-binding protein 1	YBX1	36 kDa	0.4

Fermitin family homolog 2	FERMT2	78 kDa	0.4
Arginine--tRNA ligase, cytoplasmic	RARS	75 kDa	0.4
Importin subunit alpha-4	KPNA3	58 kDa	0.4
Erythrocyte band 7 integral membrane protein	STOM	32 kDa	0.4
Glutathione S-transferase P	GSTP1	23 kDa	0.4
Heat shock protein HSP 90-beta	HSP90AB1	83 kDa	0.4
Tensin-1	TNS1	186 kDa	0.4
Elongin-C	ELOC	12 kDa	0.4
UV excision repair protein RAD23 homolog B	RAD23B	43 kDa	0.4
Ubiquitin-like modifier-activating enzyme 1	UBA1	118 kDa	0.4
E3 UFM1-protein ligase 1	UFL1	90 kDa	0.4
Poly(rC)-binding protein 1	PCBP1	37 kDa	0.4
Plasminogen activator inhibitor 1 RNA-binding protein	SERBP1	45 kDa	0.4
Serpin B6	SERPINB6	43 kDa	0.4
Importin-5	IPO5	124 kDa	0.4
Alpha-adducin	ADD1	81 kDa	0.4
Branched-chain-amino-acid aminotransferase, cytosolic	BCAT1	43 kDa	0.4
Multiple coagulation factor deficiency protein 2	MCFD2	16 kDa	0.4
Splicing factor, proline- and glutamine-rich	SFPQ	76 kDa	0.4
Valine--tRNA ligase	VARS	140 kDa	0.4
40S ribosomal protein S15	RPS15	17 kDa	0.4
Calcium-binding mitochondrial carrier protein SCaMC-1	SLC25A24	53 kDa	0.4
Eukaryotic peptide chain release factor subunit 1	ETF1	49 kDa	0.4
Serine/threonine-protein phosphatase 2A 65 kDa regulatory subunit A alpha isoform	PPP2R1A	65 kDa	0.4
ATP-dependent 6-phosphofructokinase, liver type	PFKL	85 kDa	0.4
Interleukin enhancer-binding factor 2	ILF2	43 kDa	0.4
Proteasome subunit alpha type-5	PSMA5	26 kDa	0.4
DNA damage-binding protein 1	DDB1	127 kDa	0.4
Adenylate kinase isoenzyme 1	AK1	22 kDa	0.4
T-complex protein 1 subunit theta	CCT8	60 kDa	0.4

Niban-like protein 1	FAM129 B	84 kDa	0.4
HLA class I histocompatibility antigen, A-3 alpha chain	HLA-A	41 kDa	0.4
Aspartate--tRNA ligase, cytoplasmic	DARS	57 kDa	0.4
60S ribosomal protein L35	RPL35	15 kDa	0.4
GTPase NRas	NRAS	21 kDa	0.4
Proteasome subunit alpha type-1	PSMA1	30 kDa	0.4
Transketolase	TKT	68 kDa	0.4
Nucleoprotein TPR	TPR	267 kDa	0.4
Probable ATP-dependent RNA helicase DDX5	DDX5	69 kDa	0.3
ATP-citrate synthase	ACLY	121 kDa	0.3
Apoptosis inhibitor 5	API5	59 kDa	0.3
T-complex protein 1 subunit gamma	CCT3	61 kDa	0.3
Glutathione S-transferase omega-1	GSTO1	28 kDa	0.3
60S ribosomal protein L26	RPL26	17 kDa	0.3
Golgi resident protein GCP60	ACBD3	61 kDa	0.3
ATP-dependent 6-phosphofructokinase, platelet type	PFKP	86 kDa	0.3
DNA-dependent protein kinase catalytic subunit	PRKDC	469 kDa	0.3
Pyrroline-5-carboxylate reductase 1, mitochondrial	PYCR1	33 kDa	0.3
Eukaryotic translation initiation factor 3 subunit B	EIF3B	92 kDa	0.3
Tyrosine--tRNA ligase, cytoplasmic	YARS	59 kDa	0.3
SUN domain-containing protein 2	SUN2	80 kDa	0.3
Dolichol-phosphate mannosyltransferase subunit 1	DPM1	30 kDa	0.3
Apolipoprotein L2	APOL2	37 kDa	0.3
LIM domain and actin-binding protein 1	LIMA1	85 kDa	0.3
Peroxiredoxin-6	PRDX6	25 kDa	0.3
Nucleolar RNA helicase 2	DDX21	87 kDa	0.3
T-complex protein 1 subunit beta	CCT2	57 kDa	0.3
T-complex protein 1 subunit epsilon	CCT5	60 kDa	0.3
26S proteasome regulatory subunit 6B	PSMC4	47 kDa	0.3
Eukaryotic initiation factor 4A-I	EIF4A1	46 kDa	0.3
Eukaryotic translation initiation factor 5	EIF5	49 kDa	0.3
Histone deacetylase 2	HDAC2	55 kDa	0.3
40S ribosomal protein S30	FAU	7 kDa	0.3
Matrix metalloproteinase-14	MMP14	66 kDa	0.3
ATP-binding cassette sub-family F member 2	ABCF2	71 kDa	0.3
Purine nucleoside phosphorylase	PNP	32 kDa	0.3
Lupus La protein	SSB	47 kDa	0.3
Inactive C-alpha-formylglycine-generating enzyme 2	SUMF2	34 kDa	0.3
Puromycin-sensitive aminopeptidase	NPEPPS	103 kDa	0.3
CDK5 regulatory subunit-associated protein 3	CDK5RAP 3	57 kDa	0.3
NADPH--cytochrome P450 reductase	POR	77 kDa	0.3
Serine/arginine-rich splicing factor 1	SRSF1	28 kDa	0.3
Hsp90 co-chaperone Cdc37	CDC37	44 kDa	0.3
Lysosomal alpha-glucosidase	GAA	105 kDa	0.3
Interferon-induced, double-stranded RNA-activated protein kinase	EIF2AK2	62 kDa	0.3
Lon protease homolog, mitochondrial	LONP1	106 kDa	0.3
Double-stranded RNA-specific adenosine deaminase	ADAR	136 kDa	0.3
ATPase family AAA domain-containing protein 3A	ATAD3A	71 kDa	0.3
26S proteasome regulatory subunit 8	PSMC5	46 kDa	0.3
D-3-phosphoglycerate dehydrogenase	PHGDH	57 kDa	0.3
Transportin-1	TNPO1	102 kDa	0.3
X-ray repair cross-complementing protein 5	XRCC5	83 kDa	0.3
Coatomer subunit delta	ARCN1	57 kDa	0.3
Splicing factor 3A subunit 1	SF3A1	89 kDa	0.3
Serine-threonine kinase receptor-associated protein	STRAP	38 kDa	0.3

Protein SEC13 homolog	SEC13	36 kDa	0.2
Coatomer subunit gamma-1	COPG1	98 kDa	0.2
Thyroid hormone receptor-associated protein 3	THRAP3	109 kDa	0.2
Constitutive coactivator of PPAR-gamma-like protein 1	FAM120A	122 kDa	0.2
Proteasome subunit alpha type-4	PSMA4	29 kDa	0.2
Pentraxin-related protein PTX3	PTX3	42 kDa	0.2
Transforming growth factor-beta-induced protein ig-h3	TGFBI	75 kDa	0.2
HLA class I histocompatibility antigen, Cw-7 alpha chain	HLA-C	41 kDa	0.2
Golgi-associated plant pathogenesis-related protein 1	GLIPR2	17 kDa	0.2
GTP-binding nuclear protein Ran	RAN	24 kDa	0.2
Fascin	FSCN1	55 kDa	0.2
Eukaryotic translation initiation factor 3 subunit C	EIF3C	105 kDa	0.2
Splicing factor 3B subunit 2	SF3B2	100 kDa	0.2
Eukaryotic initiation factor 4A-III	EIF4A3	47 kDa	0.2
Bifunctional glutamate/proline--tRNA ligase	EPRS	171 kDa	0.2
26S proteasome non-ATPase regulatory subunit 13	PSMD13	43 kDa	0.2
T-complex protein 1 subunit delta	CCT4	58 kDa	0.2
Stress-induced-phosphoprotein 1	STIP1	63 kDa	0.2
Translationally-controlled tumor protein	TPT1	20 kDa	0.2
Tryptophan--tRNA ligase, cytoplasmic	WARS	53 kDa	0.2
Glucose-6-phosphate 1-dehydrogenase	G6PD	59 kDa	0.2
Histone H1.2	HIST1H1C	21 kDa	0.2
T-complex protein 1 subunit alpha	TCP1	60 kDa	0.2
RuvB-like 1	RUVBL1	50 kDa	0.2
Coatomer subunit beta	COPB1	107 kDa	0.2
Isoleucine--tRNA ligase, cytoplasmic	IARS	145 kDa	0.2
Serine--tRNA ligase, cytoplasmic	SARS	59 kDa	0.2

Myosin light chain 1/3, skeletal muscle isoform	MYL1	21 kDa	0.2
EH domain-containing protein 4	EHD4	61 kDa	0.2
Band 4.1-like protein 3	EPB41L3	121 kDa	0.2
Asparagine--tRNA ligase, cytoplasmic	NARS	63 kDa	0.2
Ras suppressor protein 1	RSU1	32 kDa	0.2
Coatomer subunit alpha	COPA	138 kDa	0.2
Far upstream element-binding protein 2	KHSRP	73 kDa	0.2
Lamin-B1	LMNB1	66 kDa	0.2
Platelet-derived growth factor receptor beta	PDGFRB	124 kDa	0.2
Eukaryotic translation initiation factor 3 subunit A	EIF3A	167 kDa	0.2
Glycine--tRNA ligase	GARS	83 kDa	0.1
Threonine--tRNA ligase, cytoplasmic	TARS	83 kDa	0.1
Splicing factor 3B subunit 1	SF3B1	146 kDa	0.1
High mobility group protein HMG-I/HMG-Y	HMGA1	12 kDa	0.1
Phosphoserine aminotransferase	PSAT1	40 kDa	0.1
14-3-3 protein eta	YWHAH	28 kDa	0.1
T-complex protein 1 subunit eta	CCT7	59 kDa	0.1
Transcription intermediary factor 1-beta	TRIM28	89 kDa	0.08

Supplementary Table S6. Upstream analysis of DEPs (P12/P4 hASCs)

Upstream Regulator	Molecule Type	Predicted Activation State	Activation z-score	p-value of overlap	Target molecules in dataset
ST1926	chemical drug	Activated	4.69	1.06E-14	ARPC3,CCT2,CCT3,CCT6A,DYNLL1,EEF2,EIF3D,EIF4A1,EIF5A,ENO1,ILF2,NACA,NARS,PCBP2,PSMA4,PSMB1,RPS10,RPS15,SEPT7,SRP72,TPT1,YBX1
CD 437	chemical drug	Activated	4.6	2.01E-15	API5,ARPC3,ATP5F1C,CCT2,CCT3,CCT6A,EEF2,EIF3D,EIF4A1,EIF5A,ENO1,FSCN1,HLA-C,HNRNPA1,ILF2,NACA,NARS,PCBP1,PCBP2,PSMA4,PSMB1,RPS10,RPS15,SEPT7,YBX1
5-fluorouracil	chemical drug	Activated	4.14	1.57E-10	ATP5F1C,CCT3,CCT4,CCT6A,EEF2,EIF3C,EIF4A1,GARS,HNRNPA1,HSP90AB1,IARS,ILF2,NACA,PCBP1,PSMA7,PSMB1,PSMD2,RPL35,RPS28,SERPINB6,SSB,YBX1
valproic acid	chemical drug	Activated	3.534	6.3E-09	ATP2B4,BAG2,FSCN1,HNRNPA1,ICAM1,LIMA1,PDGFRB,PSMA1,PSMA4,PSMA5,PSMA7,PSMB1,PSMB4,PSMC2,PSMC3,PSMC4,PSMC5,PSMD13,PSMD14,PSMD2,PSMD4,PTX3,SERPINE1,SRSF1,TNS1,UTRN,YWHAH
CST5	other	Activated	2.982	8.61E-08	ABCC1,CEMIP,COL12A1,DDX21,DHX9,HNRNPU,MSN,MYL9,PCBP1,PDLIM5,PRPF8,PUF60,RBM3,RSL1D1,RTN3,SRSF1,SRSF9,TARDBP,VARS
sirolimus	chemical drug	Activated	2.878	5.59E-16	ACTA2,ATP2B4,ATP5F1C,COL1A1,CTGF,EEF1A1,EEF2,EIF2AK2,EIF4A1,EIF5,ENO1,FSCN1,FUS,G6PD,GSTP1,HIST1H4A,HLA-B,HNRNPU,HSP90AB1,ICAM1,MMP14,NDUFA9,PDGFRB,PFKP,PLIN2,PSMA4,PSMB1,PSMB4,RAN,RPL35,RPS10,RPS15,SERPINE1,SLC1A5,SLC2A1,TARS,TPM3,TRIM25,UBA1
mir-122	microRNA	Activated	2.714	1.62E-07	ACTR1A,CSRP1,G6PD,MAPRE1,NPEPPS,NUTF2,RBM3,SERPINB6,SLC1A5,WARS,YARS
KLF3	transcription regulator	Activated	2.714	0.0131	CCT3,EIF5A,EPRS,GLIPR2,GSTP1,MSN,PFKP,PSMC5,RUVBL1,SEC24D,TPM3
RICTOR	other	Activated	2.711	1.17E-11	ATP5F1C,ATP6V0A1,ATP6V0D1,FAU,NDUFA9,PPA1,PSMA1,PSMA4,PSMA5,PSMA7,PSMB1,PSMB4,PSMC2,PSMC3,PSMC4,PSMC5,PSMD13,PSMD14,PSMD2,PSMD4,RPL26,RPS10,RPS15
EDN1	cytokine	Activated	2.542	3.28E-06	ACTA2,BAG2,CTGF,ICAM1,ITGA2,ITGB1,MPRIP,MSN,SERPINE1,SF3B2,SLC2A1,TPM2,TPM3,TPT1
cigarette smoke	chemical toxicant	Activated	2.434	0.000513	ACTA2,CCT5,CKB,G6PD,GSTP1,HDAC2,ICAM1,SERPINE1,SLC2A1,SOD2,TCP1,UGDH
fatty acid	chemical - endogenous mammalian	Activated	2.414	0.00566	ACOX1,ATP5F1C,PLIN2,PPA1,SERPINE1,SLC2A1
SRF	transcription regulator	Activated	2.394	0.0061	ACTA2,CAPRIN1,CNN1,CSRP1,CSRP2,CTGF,FERMT2,ITGB1,MYL1,MYL9,SLC2A1,TGFB1I1
sphingosine-1-phosphate	chemical - endogenous mammalian	Activated	2.366	0.0026	ACTA2,CTGF,HDAC2,ICAM1,MMP14,SERPINE1
tretinoin	chemical - endogenous mammalian	Activated	2.327	3.89E-07	ACOX1,ADAR,CCT5,COL1A1,CTGF,DPP4,DYNLL1,ECI2,EEF1A1,EIF2AK2,EIF3B,EIF3C,EIF4A1,EIF4G1,EIF5,ELOC,FUBP3,GARS,GNA13,GSTP1,HLA-A,HLA-B,HLA-C,HMGA1,ICAM1,ILF3,ITGA2,ITGB1,KRT14,LOXL2,MMP14,NME2,PDGFRB,PLIN2,PNP,POR,PSMC4,PTX3,RAD23B,RAI14,RAN,RUVBL1,SERPINE1,SERPINE2,TGFBI,TPR,TPT1,UBA1,XRCC5,XRCC6,YBX1
RELA	transcription regulator	Activated	2.302	0.0146	ACTA2,ALCAM,COL1A1,CTGF,FSCN1,HLA-B,ICAM1,KRT8,PRDX6,PTX3,SERPINE2,SOD2,STIM1
IND S7	chemical - kinase inhibitor	Activated	2.236	6.68E-05	ENO1,HNRNPL,KHSRP,PSMA1,STIP1
MEL S3	chemical - kinase inhibitor	Activated	2.236	7.97E-05	ENO1,HNRNPL,KHSRP,PSMA1,STIP1
4-hydroxytamoxifen	chemical drug	Activated	2.228	0.00786	CTGF,DDX21,DYNLL1,EIF5A,GLRX,SLC25A4,SLC2A1,TNPO1
eicosapentenoic acid	chemical drug	Activated	2.213	0.0148	ECH1,PLIN2,PSMC2,SERPINE1,SLC2A1
MRTFA	transcription regulator	Activated	2.204	0.0161	ACTA2,CNN1,HLA-A,ICAM1,ITGB1,MYL9
ITGB1	transmembrane receptor	Activated	2.184	0.0599	ABCC1,ACTA2,COL1A1,ICAM1,SERPINE1

CDKN2A	transcription regulator	Activated	2.158	0.0145	AK1,CTGF,DDX5,EEF1A1,GNA13,PDGFRB,PSMA5,PTX3,SERPINE1,TRIM28
SMAD2	transcription regulator	Activated	2.116	0.00206	ACTA2,CTGF,ITGB1,SERPINE1,TPM2,TPM3
SFRP1	transmembrane receptor	Activated	2	0.00886	ACLY,ACTA2,COL1A1,SERPINE1
CD3	complex	Inhibited	-2.024	6.71E-09	ATP5F1C,DPP4,EIF4A3,FKBP3,G6PD,GDI2,HDAC2,HNRNPA1,HNRNPR,ICAM1,ILF2,KRT14,LNPEP,LONP1,PCBP2,PLEC,PLIN2,PNP,PPA1,PSMA5,RAD23B,RARS,RBM3,SRSF1,SSB,STIM1,STOM,TARDBP,THY1,TRIM25,XRCC6,YARS
RUNX2	transcription regulator	Inhibited	-2.074	0.0394	ACTA2,COL1A1,MMP14,NEXN,SERPINE1
EGFR	kinase	Inhibited	-2.093	2.44E-07	ACTA2,ATAD3A,CCT5,COL1A1,CTGF,EIF5A,HMGA1,HNRNPA1,ICAM1,ITGA2,MARCKS,MMP14,NUTF2,PSMB1,PSMB4,RAI14,SERBP1,SERPINE1,SERPINE2,SLC2A1,SRSF1,UBA1
tunicamycin	chemical - endogenous non-mammalian	Inhibited	-2.099	0.00101	ACOX1,HDLBP,ICAM1,ILK,LONP1,NARS,PLIN2,SEC24D,SLC2A1,WARS
XBP1	transcription regulator	Inhibited	-2.137	0.00517	ARCN1,COPB1,COPG1,FKBP7,ICAM1,MCFD2,SEC24D,SELENOM,XRCC6
ATF4	transcription regulator	Inhibited	-2.148	2.74E-09	BCAT1,GARS,IARS,NARS,PHGDH,PRKDC,PSAT1,PTX3,PYCR1,SARS,SHMT2,SLC1A5,SLC7A5,SOD2,TKT,WARS
pimagedine	chemical drug	Inhibited	-2.219	2.97E-05	ACTA2,CNN1,CTGF,ICAM1,OPA1,SERPINE1
DDIT3	transcription regulator	Inhibited	-2.236	0.0348	ITGA2,PLEC,SARS,SRSF1,WARS
bortezomib	chemical drug	Inhibited	-2.261	7.03E-05	ABCF2,HDAC2,ICAM1,PSMA1,PSMA5,PSMA7,PSMB4,PSMC4,PSMD13,PSMD14,PSMD2,PSMD4,UFD1
simvastatin	chemical drug	Inhibited	-2.341	0.044	ACTA2,CTGF,HIST1H1C,ICAM1,MYH10,NONO,SERPINE1
STAT6	transcription regulator	Inhibited	-2.619	0.191	ACOX1,COL1A1,FSCN1,GNB4,MMP14,SERPINB6,SERPINE1,THY1
NFE2L1	transcription regulator	Inhibited	-2.646	5.21E-07	GSTP1,PSMA1,PSMA7,PSMB1,PSMC2,PSMC3,PSMD14
beta-estradiol	chemical - endogenous mammalian	Inhibited	-2.71	6.66E-10	ACOX1,ACTA2,ALCAM,ATP2B1,BCAT1,CCT2,CKB,CNN1,COL1A1,CTGF,DDX17,DYNLL1,ECH1,EIF3A,EIF3B,EIF3D,ELOC,ENO1,EPB41L3,FARSA,FLOT1,G6PD,GARS,GDI2,GNS,GSTP1,HDAC2,HDLBP,HLA-A,HNRNPA1,HSP90AB1,IARS,ICAM1,ILF3,ITGA2,KRT8,LIMA1,NARS,NOP58,PDGFRB,PFKL,PLIN2,PNP,PSAT1,PSMA1,PSMB1,PSMC3,PTX3,PYCR1,RAN,RARS,RDH10,SLC12A4,SLC2A1,SLC7A5,SRSF1,STIP1,SYNE1,TARDBP,TARS,TIMM13,TPR,TRIM25,YARS,YWHAH
HSF1	transcription regulator	Inhibited	-2.789	4.79E-05	CCT2,CCT3,CCT4,CCT5,CCT6A,CCT7,CCT8,CSRP2,HSP90AB1,ICAM1,STIP1,TCP1
UCP1	transporter	Inhibited	-2.961	0.000968	EEF1A1,G6PD,GARS,GSTO1,PHGDH,PSAT1,PYCR1,SERPINE1,SHMT2
2-amino-1-methyl-6-phenylimidazo-4-5-b-pyridine	chemical toxicant	Inhibited	-3	1.79E-08	CCT2,CCT4,CCT5,CCT7,CCT8,PSMB1,PSMB4,RAN,UBA1
MYCN	transcription regulator	Inhibited	-3.044	6.71E-14	ABCC1,ABCC4,ABCF2,COL1A1,CTGF,EEF1A1,EEF2,EIF3C,EIF4A1,EIF5A,FAU,HDAC2,HLA-A,HMGA1,HSP90AB1,ITGA2,ITGB1,NACA,NME2,PHGDH,PSMA7,RPL26,RPL35,RPS15,RPS28,SERPINE1,SLC2A1
MYC	transcription regulator	Inhibited	-3.242	4.71E-18	ABCC1,ABCC4,ABCF2,ADD1,ALCAM,ATAD3A,BCAT1,CCT3,COL1A1,CSRP2,DDX21,DDX39B,DDX5,EEF2,EIF3D,EIF4A1,EIF4G1,ENO1,G6PD,GAA,GDI2,GFPT1,GGH,GPI,HDAC2,HLA-A,HLA-B,HMGA1,HNRNPA1,HNRNPU,ICAM1,ITGB1,KRT14,LIMA1,MSN,MYL9,NARS,NME2,NOP58,PDGFRB,PFKL,PFKP,PSAT1,PYCR1,RARS,RPL26,RPL35,RUVBL1,SERPINE1,SERPINE2,SHMT2,SLC1A5,SLC2A1,SLC7A5,SOD2,SRSF1,THY1,TKT,VARS,YBX1
HSF2	transcription regulator	Inhibited	-3.317	6.98E-12	CCT2,CCT3,CCT4,CCT5,CCT6A,CCT7,CCT8,PSMA1,PSMA5,PSMC4,TCP1
NFE2L2	transcription regulator	Inhibited	-4.431	1.08E-19	ABCC1,ABCC4,CCT3,CCT7,COL1A1,CTGF,DDX39B,DYNLL1,EIF3C,G6PD,GSTO1,GSTP1,HSP90AB1,MCFD2,NARS,PCBP1,PHGDH,PLIN2,PSAT1,PSMA1,PSMA4,PSMA5,PSMA7,PSMB1,PSMB4,PSMC3,PSMD13,PSMD14,PSMD4,RAN,RARS,RUVBL1,SERPINE1,SHMT2,SLC2A1,SOD2,STIP1,TKT,UGDH
1,2-dithiol-3-thione	chemical reagent	Inhibited	-4.965	7.18E-19	CCT3,CCT7,DDX39B,DYNLL1,EIF3C,EIF3D,GSTP1,HSP90AB1,MCFD2,NARS,PCBP1,PSAT1,PSMA1,PSMA4,PSMA5,PSMA7,PSMB1,PSMB4,PSMC2,PSMC3,PSMD13,PSMD14,PSMD4,RAN,RARS,RUVBL1,SOD2,STIP1

Supplementary Table S7. DEPs overlapped between P8/P4 and P12/P4 hASCs. A total of 28 DEPs were overlapped with P4, P8 and P12 hASCs, 19 DEPs have the same changing trend between P8/P4 and P12/P4 comparisons.

Protein name	Gene name	Molecular Weight	Fold Change by p8/p4	Fold Change by p12/p4
ADP/ATP translocase 1 OS=Homo sapiens OX=9606 GN=SLC25A4 PE=1 SV=4	ARPC3	21 kDa	-2.5	-2.5
Actin-related protein 2/3 complex subunit 3 OS=Homo sapiens OX=9606 GN=ARPC3 PE=1 SV=3	ASAH1	45 kDa	-2	2.9
Acid ceramidase OS=Homo sapiens OX=9606 GN=ASAH1 PE=1 SV=5	CNN1	33 kDa	-3.3	22
Calponin-1 OS=Homo sapiens OX=9606 GN=CNN1 PE=1 SV=2	CSRP2	21 kDa	-2.5	4.4
Cysteine and glycine-rich protein 2 OS=Homo sapiens OX=9606 GN=CSRP2 PE=1 SV=3	CTSA	54 kDa	2.7	3.8
Drebrin-like protein OS=Homo sapiens OX=9606 GN=DBNL PE=1 SV=1	DBNL	48 kDa	-2.5	-2.5
Delta(3,5)-Delta(2,4)-dienoyl-CoA isomerase, mitochondrial OS=Homo sapiens OX=9606 GN=ECH1 PE=1 SV=2	ECH1	36 kDa	3.8	2.7
Enoyl-CoA delta isomerase 2, mitochondrial OS=Homo sapiens OX=9606 GN=ECI2 PE=1 SV=4	ECI2	44 kDa	5.2	2.5
Flotillin-1 OS=Homo sapiens OX=9606 GN=FLOT1 PE=1 SV=3	FLOT1	47 kDa	2.2	5.5
Glutamine--fructose-6-phosphate aminotransferase [isomerizing] 1 OS=Homo sapiens OX=9606 GN=GFPT1 PE=1 SV=3	GFPT1	79 kDa	-2	-2.5
Keratin, type I cytoskeletal 14 OS=Homo sapiens OX=9606 GN=KRT14 PE=1 SV=4	KRT14	52 kDa	6.6	18
Keratin, type II cytoskeletal 8 OS=Homo sapiens OX=9606 GN=KRT8 PE=1 SV=7	KRT8	54 kDa	3.3	-2
Lon protease homolog, mitochondrial OS=Homo sapiens OX=9606 GN=LONP1 PE=1 SV=2	LONP1	106 kDa	2.6	-3.3
Microtubule-associated protein RP/EB family member 1 OS=Homo sapiens OX=9606 GN=MAPRE1 PE=1 SV=3	MAPRE1	30 kDa	-2.5	-2.5
Myosin-10 OS=Homo sapiens OX=9606 GN=MYH10 PE=1 SV=3	MYH10	229 kDa	-3.3	4.4
Myosin light chain 1/3, skeletal muscle isoform OS=Homo sapiens OX=9606 GN=MYL1 PE=2 SV=3	MYL1	21 kDa	-5	-5
NADH dehydrogenase [ubiquinone] 1 alpha subcomplex subunit 9, mitochondrial OS=Homo sapiens OX=9606 GN=NDUFA9 PE=1 SV=2	NDUFA9	43 kDa	2.2	2
Pyrroline-5-carboxylate reductase 1, mitochondrial OS=Homo sapiens OX=9606 GN=PYCR1 PE=1 SV=2	NPEPPS	103 kDa	-2.5	-3.3
ATP-dependent 6-phosphofructokinase, platelet type OS=Homo sapiens OX=9606 GN=PFKP PE=1 SV=2	PFKP	86 kDa	-2.5	-3.3
Polycystin-2 OS=Homo sapiens OX=9606 GN=PKD2 PE=1 SV=3	PKD2	110 kDa	2.1	2.6
Purine nucleoside phosphorylase OS=Homo sapiens OX=9606 GN=PNP PE=1 SV=2	PNP	32 kDa	-3.3	-3.3
Lysosomal protective protein OS=Homo sapiens OX=9606 GN=CTSA PE=1 SV=2	PYCR1	33 kDa	2.7	-3.3
Puromycin-sensitive aminopeptidase OS=Homo sapiens OX=9606 GN=NPEPPS PE=1 SV=2	RPL26	17 kDa	-2	-3.3
60S ribosomal protein L26 OS=Homo sapiens OX=9606 GN=RPL26 PE=1 SV=1	RRAS2	23 kDa	2.4	5.3
Ras-related protein R-Ras2 OS=Homo sapiens OX=9606 GN=RRAS2 PE=1 SV=1	SLC25A4	33 kDa	-2.5	3.1
Sorbin and SH3 domain-containing protein 2 OS=Homo sapiens OX=9606 GN=SORBS2 PE=1 SV=3	SORBS2	124 kDa	-2.5	5.1
Mitochondrial import inner membrane translocase subunit Tim13 OS=Homo sapiens OX=9606 GN=TIMM13 PE=1 SV=1	TIMM13	11 kDa	2.5	2.8
Ubiquitin carboxyl-terminal hydrolase 5 OS=Homo sapiens OX=9606 GN=USP5 PE=1 SV=2	USP5	96 kDa	-2.5	-3.3
